# Clinical Outcomes of Magnetic Seizure Therapy vs Electroconvulsive Therapy for Major Depressive Episode: A Randomized Clinical Trial

**DOI:** 10.1001/jamapsychiatry.2023.4599

**Published:** 2023-12-06

**Authors:** Zhi-De Deng, Bruce Luber, Shawn M. McClintock, Richard D. Weiner, Mustafa M. Husain, Sarah H. Lisanby

**Affiliations:** 1 Noninvasive Neuromodulation Unit, Experimental Therapeutics Branch, Intramural Research Program, National Institute of Mental Health, Bethesda, Maryland; 2 Department of Psychiatry and Behavioral Sciences, Duke University School of Medicine, Durham, North Carolina; 3 Department of Psychiatry, University of Texas Southwestern Medical Center, Dallas

## Abstract

**Importance:**

Electroconvulsive therapy (ECT) is highly effective and rapid in treating depression, but it carries a risk of significant cognitive adverse effects. Magnetic seizure therapy (MST), an investigational antidepressant treatment, may maintain the robust antidepressant efficacy of ECT while substantially reducing adverse effects due to its enhanced focality and weaker stimulation strength; however, previous clinical trials of MST were limited by small sample sizes.

**Objective:**

To compare the antidepressant efficacy of MST vs ultrabrief pulse right unilateral (RUL) ECT.

**Design, Setting, and Participants:**

A between-participants, double-blinded, randomized clinical trial was conducted at 3 academic hospitals from June 2007 to August 2012. Adults aged 18 to 90 years who were referred for treatment with ECT, had a major depressive episode in the context of major depressive disorder or bipolar disorder, and had a baseline 24-item Hamilton Depression Rating Scale (HDRS-24) total score of 18 or higher were included. Participants were randomly assigned 1:1 to treatment with MST or ultrabrief pulse RUL ECT. After the treatment course, patients were naturalistically followed up for up to 6 months to examine the durability of clinical effects.

**Interventions:**

Treatment with MST, applied at 100 Hz at 100% of the maximum device power for 10 seconds, or ultrabrief pulse RUL ECT, applied at 6 times seizure threshold.

**Main Outcomes and Measures:**

The primary outcome was change from baseline in HDRS-24 total score, with patients followed up for up to 6 months. A reduction of at least 50% in the HDRS-24 score indicated response, and at least a 60% decrease in the HDRS-24 score and a total score of 8 or less indicated remission.

**Results:**

Of the 73 participants (41 [56.2%] female; mean [SD] age, 48 [14.1] years), 35 were randomized to MST and 38 to ECT. Among them, 53 (72.6%) were classified as completers (29 in the MST group and 24 in the ECT group). Both MST and ECT demonstrated clinically meaningful antidepressant effects. In the intent-to-treat sample, 18 participants (51.4%) in the MST group and 16 (42.1%) in the ECT group met response criteria; 13 (37.1%) in the MST group and 10 (26.3%) in the ECT group met remission criteria. Among completers, 17 of 29 (58.6%) in the MST group and 15 of 24 (62.5%) in the ECT group met response criteria; 13 of 29 (44.8%) in the MST group and 10 of 24 (41.7%) in the ECT group met remission criteria. There was no significant difference between MST and ECT for either response or remission rates. However, the mean (SD) number of treatments needed to achieve remission was 9.0 (3.1) with MST and 6.7 (3.3) with ECT, a difference of 2.3 treatments (*t*_71.0_ = 3.1; *P* = .003). Both MST and ECT showed a sustained benefit over a 6-month follow-up period, again with no significant difference between them. Compared with MST, ECT had significantly longer time to orientation after treatment (threshold level: *F*_1,56_ = 10.0; *P* = .003) and greater severity of subjective adverse effects, particularly in the physical and cognitive domains.

**Conclusions and Relevance:**

This randomized clinical trial found that the efficacy of MST was indistinguishable from that of ultrabrief pulse RUL ECT, the safest form of ECT currently available. These results support the continued development of MST and provide evidence for advantages relative to state-of-the-art ECT.

**Trial Registration:**

ClinicalTrials.gov Identifier: NCT00488748

## Key Points

**Question:**

Is magnetic seizure therapy (MST) as effective as electroconvulsive therapy (ECT) in patients with major depressive episode?

**Findings:**

In this randomized clinical trial including 73 patients, assessments of antidepressant response showed significantly decreased Hamilton Depression Rating Scale scores with treatment with either MST or ECT, with no significant difference in response or remission rates. The speed of response was similar between MST and ECT, although ECT showed faster time to remission; the time to orientation after treatment was significantly faster with MST than ECT.

**Meaning:**

The findings suggest that MST confers antidepressant benefit while maximizing cognitive safety.

## Teaser

This randomized clinical trial investigates whether magnetic seizure therapy is as efficacious as ultrabrief pulse right unilateral electroconvulsive therapy in treating patients with major depressive episode.

## Introduction

**Figure 1.**
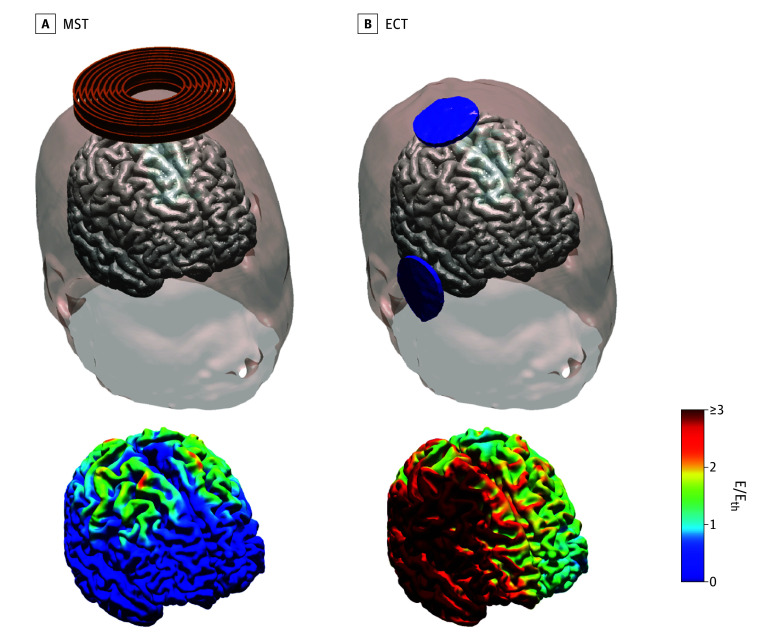
Head Modeling of the Electric Field Induced in the Brain With the Magnetic Seizure Therapy (MST) and Right Unilateral Electroconvulsive Therapy (ECT) Conditions Used in This Trial MST (A) induces more focal electric fields in the brain than ECT (B). E indicates induced electric field magnitude; E_th_, electric field threshold for neuronal activation.

Major depressive disorder (MDD), a leading cause of global disability, is associated with significant morbidity and mortality.^1^ While electroconvulsive therapy (ECT) is highly effective, especially for patients with psychosis, treatment resistance, or acute suicide risk, it carries the risk of adverse neurocognitive effects. Magnetic seizure therapy (MST), designed with the goal of matching the efficacy of ECT while maximizing cognitive safety, involves seizure induction with transcranial magnetic stimulation (TMS) while the patient is under anesthesia.^2,3,4^ Electromagnetic induction via MST offers the ability to induce seizures with more focal and less intense stimulation than is possible with ECT (Figure 1).^5^ Four case series of patients with major depressive episodes (MDEs) found that MST decreased depression, produced rapid reorientation, and spared global cognitive abilities.^2,6,7,8^ Within-participant studies in patients with MDD found quicker reorientation and preserved global cognitive function with MST compared with ECT.^4,9^ Similarly, preclinical models have found better cognitive outcomes with MST compared with ECT.^10,11,12^

An open-label study of MST^13^ reported antidepressant benefits and few cognitive adverse effects, consistent with 2 earlier open-label studies.^14,15^ A systematic review of 4 randomized clinical trials (RCTs) that compared ECT and MST,^16,17,18,19^ comprising a total of 86 patients with unipolar or bipolar depression, suggested that MST may have similar effect size compared with ECT.^20^ However, these studies were limited by small sample sizes and suboptimal dosing. In particular, earlier investigations assessed the efficacy of MST with right unilateral (RUL) ECT administered at 3 times seizure threshold (ST).^16,17^ Moderate-dosage RUL ECT has been shown to be less effective than high-dose (6 times ST) RUL ECT or bitemporal ECT.^21^ The current study compared high-dose RUL ECT and MST in patients with an MDE in a multicenter, double-blind RCT, with the hypothesis that MST and ECT would have similar antidepressant efficacy.

## Methods

### Participants

This study was conducted under an investigational device exemption from the US Food and Drug Administration and was approved by the institutional review boards of the New York State Psychiatric Institute, Duke University Medical Center, and University of Texas Southwestern Medical Center. The trial protocol can be found in Supplement 1. Participants provided written informed consent. The study follows the Consolidated Standards of Reporting Trials (CONSORT) reporting guideline.

**Figure 2.**
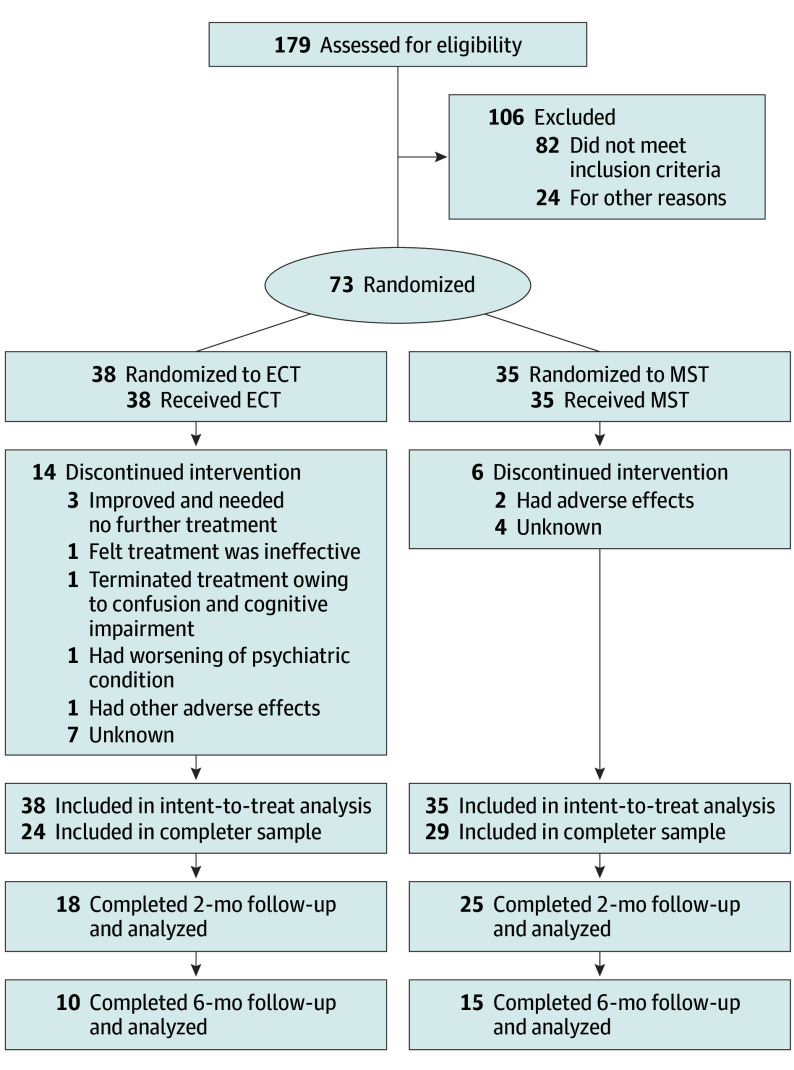
CONSORT Flow Diagram ECT indicates electroconvulsive therapy; MST, magnetic seizure therapy.

Eligible participants were men and women aged 18 to 90 years who were referred for treatment with ECT, had an MDE in the context of MDD or bipolar disorder based on the Structured Clinical Interview for *DSM-IV-TR*, and had a baseline 24-item Hamilton Depression Rating Scale^22^ (HDRS-24) score of 18 or higher (Figure 2). Exclusion criteria were a history of neurological disorders, head trauma, contraindications to TMS, current unstable or serious medical illnesses, pregnancy or breastfeeding, history of ECT in the prior 6 months or failure to previously respond to ECT, or a Mini-Mental State Examination^23^ total raw score lower than 24. Ethnicity and race data were collected using a self-report questionnaire (New York State Psychiatric Institute Research Tracking Module). Options for ethnicity included (1) Hispanic or Latino and (2) not Hispanic or Latino. Options for race included (1) American Indian or Alaska Native, (2) Asian, (3) Black or African American, (4) Native Hawaiian or Pacific Islander, (5) White, (6) mix of 2 or more races, and (7) some other race.

### Study Design

This was a 3-center, between-participants, double-blind RCT conducted from June 2007 to August 2012 that compared the antidepressant efficacy and neurocognitive effects of MST and ultrabrief pulse RUL ECT. Because of the lack of published randomized comparisons between ECT and MST at the time this study was designed, sample size was estimated based on ECT studies finding differences in clinical outcomes across different ECT conditions.^21^ In a previous RCT comparing ECT modalities at different stimulus intensities, a sample of 20 participants per group was sufficient to detect a difference in response rate as well as recovery of orientation and memory functions.^21^ In phase 1, participants were assigned 1:1 to MST or ECT with simple randomization by a computer-generated random number sequence, stratified by study site. Allocation concealment used sequentially numbered, opaque, sealed envelopes, with treatment allocation after baseline. Clinical and neuropsychology raters were blind to allocation. After the completion of phase 1, patients were naturalistically followed up for up to 6 months to examine durability of clinical effects.

### Medication

Antidepressant medications, except for lorazepam as needed up to 3 mg/d, were tapered and washed out for at least 3 days prior to the start of the treatment. The taper schedule was tailored to each patient according to their individual medications and clinical status. As is standard practice in the study site ECT service and as recommended by the American Psychiatric Association task force report on ECT,^24^ benzodiazepines were withheld for 10 hours prior to the treatment.

### Clinical Ratings

Ratings were conducted by blinded raters at baseline, the morning of each treatment session, and 24 to 72 hours after the last treatment. The primary outcome measure was the HDRS-24 total score. The clinician-rated Inventory of Depressive Symptomatology^25^ (IDS) was a secondary outcome measure. Other ratings included the self-reported IDS, Clinical Global Impressions–Improvement,^26^ and Global Assessment of Functioning.^27^ Follow-up ratings were conducted twice per month for the first 2 months, and once per month thereafter.

### Subjective Adverse Effects Evaluation

In the afternoon of each treatment day, patients were interviewed regarding subjective adverse effects using the Columbia ECT Subjective Side Effects Schedule.^28^ Patients reported on the presence (scored as absent = 0; present = 1) and severity (scored as no perceived adverse effect = 0; mild = 1; moderate = 2; severe = 3) of various adverse effects. The mean severity score was computed including instances in which patients reported no perceived adverse effects (scored as 0).^28^ Three categories of subjective effects were assessed, including physical adverse effects, cognitive adverse effects, and mood-related effects. The physical adverse effects included headache, nausea, dry mouth, muscle pain, and other aches or pains. The cognitive adverse effects included confusion and memory problems. The mood-related effects, presented as positive effects rather than adverse effects, included reports of enjoying breakfast, enjoying lunch, and feeling more active.

### Neurocognitive Testing

Global cognitive function was assessed with the Mini-Mental State Examination at baseline and within 72 hours after the last treatment session. Raw scores were converted to demographic-adjusted scaled scores. Time to orientation was assessed at each treatment after the return of spontaneous respiration. Participants were asked to open their eyes on command and correctly answer questions of name, age, date of birth, date, and name of building, until all questions were answered correctly or 60 minutes had elapsed. We also examined the effects of ECT and MST on recall of autobiographical information with the Autobiographical Memory Test. The Autobiographical Memory Test evaluates the ability to recall specific autobiographical memories within a time constraint (60 seconds), prompted by cue words of positive or negative emotional valence (eg, happy, angry, successful, lonely). Scores were tabulated for total memories recalled, total categorical memories, and total specific memories. We also collected comprehensive neurocognitive information (such as executive function, cognitive failure, and verbal learning), and those results will be reported elsewhere.

### Anesthesia

Treatments with MST and ECT were performed under the same general anesthesia, with atropine (0.4 mg) or glycopyrrolate (0.2 mg) depending on the preference of the anesthesiology team at the study sites; etomidate (0.15-0.2 mg/kg) or, in a few instances, methohexital (approximately 1 mg/kg); and succinylcholine (0.75-1.0 mg/kg). Patients were oxygenated until spontaneous respirations returned.

### ECT and MST

Ultrabrief RUL ECT was given with either a spECTrum 5000Q (MECTA LLC; 0.3-millisecond pulse width; 800-mA pulse amplitude) or a Thymatron System IV (Somatics LLC; 0.25-millisecond pulse width; 900-mA pulse amplitude). MST was delivered with a Magstim Theta device (Magstim Co Ltd) using a double-layer round coil (44-mm inner diameter; 120-mm outer diameter) positioned on the vertex.

ST was titrated at the first session. For ECT, dose titration followed published guidelines. Subsequent ECT treatments were given at 6 times ST. For MST, stimulus frequency was fixed at 100 Hz, pulse intensity was set to maximum stimulator output, and train duration was set to 10 seconds. For MST titration, the first step was given at a 5-second train duration; if there was no seizure, the duration was increased to 10 seconds. Subsequent MST treatments were given with a 10-second duration, the maximum duration for the device, for a total of 1000 pulses.

Patients were prepared in the same fashion (ie, earplugs placed, skin prepared for ECT electrode placement) regardless of randomization. The auditory stimulus of a recorded MST session was played while ECT was delivered to avoid revealing treatment allocation to individuals in neighboring rooms. A reduction of at least 50% in the HDRS-24 score was considered response. Patients received treatment 3 times per week until they achieved remission (≥60% decrease in HDRS-24 score and a total score ≤8) or until they reached a plateau in response (≤3-point decrease in HDRS-24 between a given treatment and the 2 successive treatments starting from treatment 8). If there was less than a 25% decrease in HDRS-24 score from baseline by the eighth treatment, treatment was discontinued and the patient was given routine clinical care. Completers were defined as having received at least 8 treatments or having achieved remission prior to the eighth treatment. Relapse was defined as an HDRS-24 total score of 16 or higher and at least a 10-point increase in HDRS-24 score maintained across 2 visits at least 1 week apart, the emergence of psychotic or suicidal symptoms, or requiring inpatient hospitalization.

### Statistical Analysis

An intent-to-treat (ITT) analysis was performed using the HDRS-24 score as the primary outcome measure. Effects of the treatment session on HDRS-24 score and time to orientation were analyzed using linear mixed models. A repeated effect of treatment session was used to model the covariance of the residuals with a compound symmetry structure. Patients were specified as a random factor. In addition to the main effects of treatment group (ECT vs MST) and treatment session, we assessed the group-by-session interaction. Other continuous demographic and outcome variables were analyzed with *t* tests; categorical variables were analyzed with χ^2^ analyses. For all analyses, statistical significance was defined as a 2-sided *P* value of less than .05. Finally, Kaplan-Meier analysis was used to compare time to remission between the ECT and MST groups. Statistical analysis was performed using R version 4.3.1 (R Foundation).

## Results

A total of 179 patients were assessed for eligibility, and 106 were excluded. Among the 73 enrolled patients (41 [56.2%] female; mean [SD] age, 48 [14.1] years), 35 were randomized to MST and 38 to ECT (Figure 2). Sensitivity analysis showed that this sample size would allow for the detection of an effect size of Cohen *d* = 0.67 with power of 0.8 (2-tailed α = .05). This effect size is comparable with those reported in prior studies contrasting the effects of varying ECT dosages and electrode placements.^21,29^ The 2 treatment groups in this study were balanced in demographic characteristics (age, sex, race and ethnicity proportions, years of education) and depression severity (current depressive episode, number of psychotropic medications, baseline depression score) (Table). The study cohort was severely ill; the mean (SD) duration of the current MDE was 2.4 (3.3) years, 15 patients (20.5%) had a comorbid *DSM-IV-TR* Axis I disorder, and most (64 patients [87.7%]) were resistant to psychotropic medications.

**Table.** Comparison of Baseline Patient Demographic Characteristics, Treatment Information, and Outcomes Between the ECT and MST Groups

Characteristic	All patients (N =73)	ECT (n = 38)	MST (n = 35)	Test statistic	*P* value
Age, mean (SD), y	48.0 (14.1)	48.2 (12.8)	47.7 (15.6)	*t*_65.9_ = 0.12	.90
Sex, No. (%)					
Female	41 (56.2)	19 (50.0)	22 (62.9)	χ^2^_1_ = 0.76	.38
Male	32 (43.8)	19 (50.0)	13 (37.1)		
Race and ethnicity, No. (%)^a^					
Asian	<5	<5	<5	χ^2^_1_ = 0.60	.44
Black or African American	<5	<5	<5	χ^2^_1_ < 0.001	>.99
Hispanic or Latino	<5	<5	<5	χ^2^_1_ = 0.19	.67
Native Hawaiian or Pacific Islander	<5	<5	<5	χ^2^_1_ = 0.002	.97
Not reported	<5	<5	<5	χ^2^_1_ = 0.60	.44
White	62 (84.9)	34 (89.5)	28 (80.0)	χ^2^_1_ = 0.65	.42
Education, mean (SD), y	15.8 (2.8)	15.5 (2.5)	16.3 (3.2)	*t*_64.5_ = −1.19	.24
Bipolar disorder, No. (%)	10 (13.7)	6 (15.8)	4 (11.4)	χ^2^_1_ = 0.04	.84
Current depressive episode, mean (SD), wk	124.2 (169.1)	114.5 (129.4)	135.2 (208.3)	*t*_32.6_ = −0.39	.70
No. of psychotropic medications, mean (SD)	7.6 (4.5)	7.8 (4.5)	7.3 (4.5)	*t*_62.0_ = 0.41	.68
Anesthesia or muscle relaxant dose, mean (SD), mg					
Etomidate	15.1 (5.4)	15.3 (5.9)	14.9 (5.0)	*t*_472.9_ = 0.78	.44
Succinylcholine	53.6 (31.7)	72.1 (34.7)	38.3 (18.1)	*t*_363.7_ = 14.0	<.001
Seizure threshold, mean (SD)^b^	NA	33.6 (14.8)	556 (162)	NA	NA
No. of treatments, mean (SD)	7.8 (3.4)	6.7 (3.3)	9.0 (3.1)	*t*_71.0_ = −3.1	.003
HDRS-24 score, mean (SD)					
Baseline	31 (7.1)	31.8 (7.8)	30.1 (6.4)	*t*_70.0_ = 1.06	.29
After acute course	14.9 (8.9)	14.2 (9.0)	15.5 (8.8)	*t*_52.5_ = −0.54	.59
ITT sample, No. (%)					
Responders	34 (46.6)	16 (42.1)	18 (51.4)	χ^2^_1_ = 0.32	.57
Remitters	23 (31.5)	10 (26.3)	13 (37.1)	χ^2^_1_ = 0.55	.46
Completers, No. (%)	53 (72.6)	24 (63.2)	29 (82.9)	χ^2^_1_ = 2.63	.10
Responders, No./total No. (%)	32/53 (60.4)	15/24 (62.5)	17/29 (58.6)	χ^2^_1_ < 0.001	>.99
Remitters, No./total No. (%)	23/53 (43.4)	10/24 (41.7)	13/29 (44.8)	χ^2^_1_ < 0.001	>.99
Change in score following treatment, mean (SD)					
MMSE	–0.2 (3.4)	0.16 (4.3)	–0.56 (2.0)	*t*_41.7_ = 0.85	.40
AMT					
Total memories	–0.4 (2.5)	–1.4 (3.1)	0.6 (1.1)	*t*_22.3_ = −2.8	.01
Categorical memories	0.08 (2.9)	0.16 (3.1)	0 (2.7)	*t*_35.5_ = 0.17	.87
Specific memories	–0.39 (3.1)	–1.5 (3.4)	0.68 (2.5)	*t*_32.6_ = −2.2	.03

Abbreviations: AMT, Autobiographical Memory Test; ECT, electroconvulsive therapy; HDRS-24, 24-item Hamilton Depression Rating Scale; ITT, intent-to-treat; MMSE, Mini-Mental State Examination; MST, magnetic seizure therapy; NA, not applicable.

^a^ Ethnicity and race data were collected using a self-report questionnaire (New York State Psychiatric Institute Research Tracking Module). Options for ethnicity included (1) Hispanic or Latino and (2) not Hispanic or Latino. Options for race included (1) American Indian or Alaska Native, (2) Asian, (3) Black or African American, (4) Native Hawaiian or Pacific Islander, (5) White, (6) mix of 2 or more races, and (7) some other race. Due to small sample sizes in most race and ethnicity categories, reporting is omitted to prevent identifiability.

^b^ Units of measure are millicoulombs for ECT and number of pulses for MST.

### Antidepressant Efficacy

Of the 73 patients who began treatment, 53 (72.6%) were classified as completers (29 in the MST group and 24 in the ECT group). Both MST and ECT demonstrated clinically meaningful antidepressant effects. In the ITT sample, 34 patients (46.6%), including 18 (51.4%) in the MST group and 16 (42.1%) in the ECT group, met response criteria (≥50% reduction in HDRS-24 score); 23 patients (31.5%), including 13 (37.1%) in the MST group and 10 (26.3%) in the ECT group, met remission criteria (≥60% decrease in HDRS-24 score and a total score ≤8). Among completers, 32 patients (60.4%), including 17 of 29 (58.6%) in the MST group and 15 of 24 (62.5%) in the ECT group, met response criteria; 23 patients (43.4%), including 13 of 29 (44.8%) in the MST group and 10 of 24 (41.7%) in the ECT group, met remission criteria. There was no significant difference between MST and ECT for either response or remission rates. This was consistent for the ITT sample (responders: χ^2^_1_ = 0.32; *P* = .57; remitters: χ^2^_1_ = 0.55; *P* = .46) and the 53 completers (responders: χ^2^_1_ < 0.001; *P* > .99; remitters: χ^2^_1_ < 0.001; *P* > .99).

**Figure 3.**
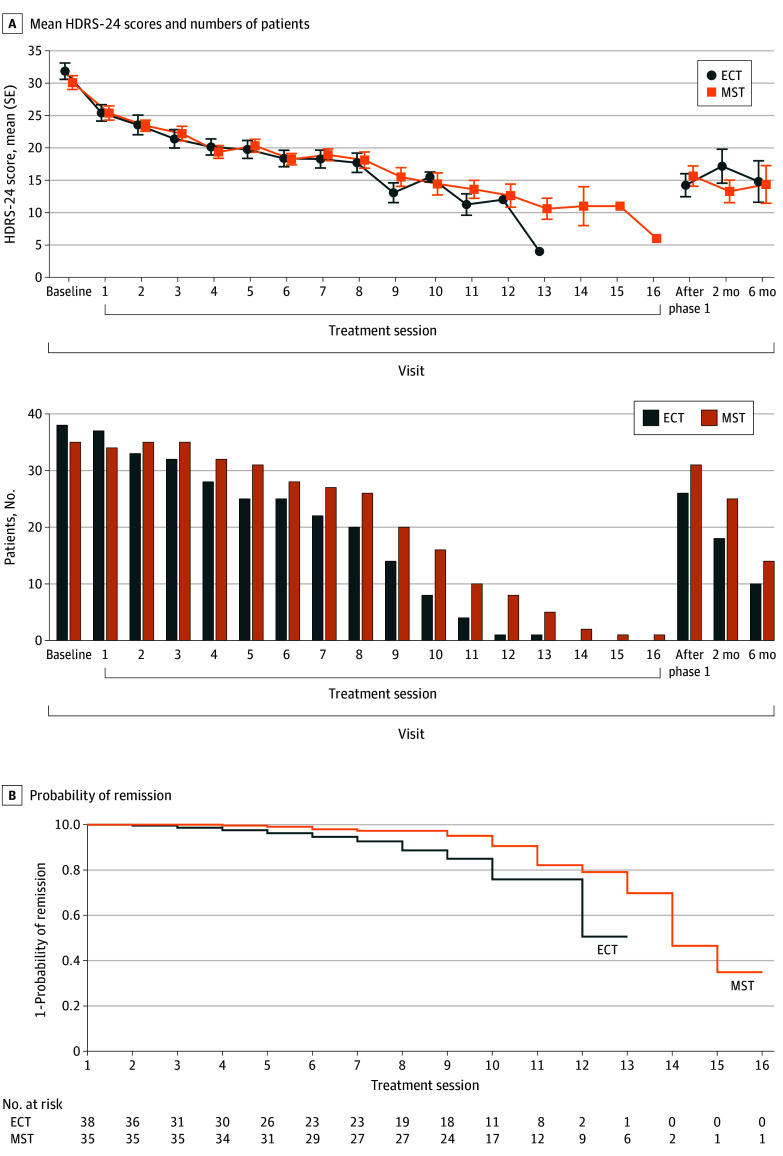
24-Item Hamilton Depression Rating Scale (HDRS-24) Scores and Kaplan-Meier Curves for the Probability of Remission Among the Magnetic Seizure Therapy (MST) and Electroconvulsive Therapy (ECT) Groups A, Mean (SE) HDRS-24 scores and numbers of patients for the MST and ECT groups in the intent-to-treat sample over the treatment course and up to 6 months of naturalistic follow-up. The mixed-effects model of the HDRS-24 showed a significant group-by-session interaction (*t*_558_ = 3.17; *P* = .002). Post hoc linear models showed that there was a significant effect of treatment session for both the ECT (*t*_248_ = −16.2; *P* < .001) and MST (*t*_310_ = −18.7; *P* < .001) groups, although the slope was larger with ECT (β = −1.76; 95% CI, −1.98 to −1.55) than MST (β = −1.36; 95% CI, −1.50 to −1.21). B, Kaplan-Meier curves for the probability of remission as a function of treatment session. ECT had faster time to remission than MST (χ^2^_1_ = 9.5; *P* = .002).

Both the MST and ECT groups showed a significant decrease in the HDRS-24 score from baseline to the end of the treatment course (Figure 3A). The groups showed similar speed of response up to the eighth session. However, when considering the entire response trajectory, the mixed-effects model of the HDRS-24 score showed a significant group-by-session interaction (*t*_558_ = 3.17; *P* = .002). Post hoc linear models showed a significant effect of treatment session for both the ECT group (*t*_248_ = −16.2; *P* < .001) and the MST group (*t*_310_ = −18.7; *P* < .001), although the slope was larger with ECT (β = −1.76; 95% CI, −1.98 to −1.55) than MST (β = −1.36; 95% CI, −1.50 to −1.21). Figure 3B shows the Kaplan-Meier curves for the probability of remission as a function of treatment session. ECT had faster time to remission compared with MST (χ^2^_1_ = 9.5; *P* = .002). Consistent with this, patients in the MST group received a mean (SD) of 9.0 (3.1) treatments, significantly more than the patients in the ECT group (mean [SD], 6.7 [3.3] treatments) (difference in means, 2.3 treatments; *t*_71.0_ = 3.1; *P* = .003).

In the analysis of variance (ANOVA) using Global Assessment of Functioning scores before and after treatment, there was a significant increase after treatment (*F*_1,46_ = 39.9; *P* < .001), with no significant difference between MST and ECT (*F*_1,46_ = 0.03; *P* = .88) or in the group-by-session interaction (*F*_1,46_ = 0.1; *P* = .80). Clinical Global Impressions–Improvement severity decreased from baseline to the end of phase 1 (*F*_1,47_ = 73.2; *P* < .001), with no significant difference between MST and ECT (*F*_1,47_ = 0.2; *P* = .66) or in the group-by-session interaction (*F*_1,47_ = 0.5; *P* = .49). The 2 × 2 mixed-model ANOVA (between-group factor, MST vs ECT; repeated-measures factor, baseline vs end of acute treatment course) using the clinician-rated IDS scores showed a significant main effect of time (*F*_1,47_ = 98.0; *P* < .001) but no significant effect of group (*F*_1,47_ = 0.1; *P* = .77) or group-by-session interaction (*F*_1,47_ = 0.1; *P* = .81). Self-reported IDS scores showed a significant main effect of time (*F*_1,42_ = 54.8; *P* < .001) but no effect of group (*F*_1,42_ = 1.6; *P* = .22) or group-by-session interaction (*F*_1,42_ = 0.0; *P* > .99) (eFigure 1 in Supplement 2).

Antidepressant benefit (as measured by the HDRS-24 score) achieved at the end of the treatment course was maintained at 2-month and 6-month follow-up (Figure 3A). At 2 months, data were available for 43 patients (25 in the MST group and 18 in the ECT group; no significant difference in the proportion of participants available: χ^2^_1_ = 3.4; *P* = .06). A 2 × 3 ANOVA with the addition of the 2-month follow-up repeated measure revealed a significant main effect of time (*F*_2,40_ = 66.5; *P* < .001) but no effect of group (*F*_1,41_ = 0.86; *P* = .36) or group-by-time interaction (*F*_2,40_ = 0.63; *P* = .55). Post hoc analysis showed that the 2-month HRSD-24 total score remained significantly improved relative to baseline (*t*_42_ = 14.8; *P* < .001). There was no significant difference between the rating at treatment end and at 2-month follow-up (*t*_42_ = 0.20; *P* = .84).

A 2 × 4 ANOVA including the 6-month follow-up data (available for 25 patients: 15 for MST and 10 for ECT; no significant difference in the proportion of participants available: χ^2^_1_ = 1.5; *P* = .21) found a significant main effect of time (*F*_2,40_ = 20.2; *P* < .001) but no effect of group (*F*_1,22_ = 0.48; *P* = .50) or group-by-time interaction (*F*_3,20_ = 0.53; *P* = .67). Post hoc analysis showed that the HRSD-24 total score remained significantly improved at 6 months relative to baseline (*t*_24_ = 5.9; *P* < .001). There was no significant difference between the end of treatment and 6-month HDRS-24 total scores (*t*_24_ = 0.61; *P* = .55).

### Seizure Characteristics

The mean (SD) ST was 33.6 (14.8) millicoulombs for ECT and 556 (162) pulses for MST. eFigure 2A in Supplement 2 shows the electroencephalogram (EEG) and motor seizure durations over the first 8 treatments. The mixed-effects model showed that mean motor seizure duration decreased as a function of treatment number (*t*_393_ = −6.38; *P* < .001) and did not differ between ECT and MST (*t*_70_ = −0.46; *P* = .65). Mean frontal EEG seizure duration also decreased as a function of treatment number (*t*_309_ = −6.72; *P* < .001) but did not differ between ECT and MST (*t*_69_ = −1.31; *P* = .20). eFigure 2C in Supplement 2 shows the session-by-session correlation between EEG seizure duration and motor seizure duration. The ratio of EEG to motor seizure duration for ECT was larger than that for MST (*t*_69_ = −3.23; *P* = .002) and did not change with treatment number (*t*_299_ = 0.027; *P* = .98), suggesting that the seizure spread beyond the motor cortex in the case of ECT but was more focal for MST. Finally, eFigure 2D in Supplement 2 shows the session-by-session correlation between EEG seizure duration and time to orientation.

### Global Cognitive Function and Time to Orientation

Global cognitive function remained intact following ECT and MST. There was no significant main effect of treatment modality (*F*_1,59_ = 1.9; *P* = .18) or time (baseline to immediately following phase 1) (*F*_1,59_ = 2.1; *P* = .16) or interaction between group and time (*F*_1,59_ = 0.4; *P* = .56). Participants regained orientation faster following MST than ECT at both threshold (*F*_1,56_ = 10.0; *P* = .003) and suprathreshold (*F*_1,56_ = 62.9; *P* < .001) levels (eFigure 2B in Supplement 2).

### Safety, Subjective Adverse Effects, and Autobiographical Memory

There were 5 serious adverse events, all in the ECT group. These included 3 cases of worsening depression resulting in hospitalization (1 occurred during the blinded phase and was associated with suicidal ideation, 1 occurred at 2-month follow-up and was associated with suicidal ideation, and 1 occurred at 6-month follow-up), 1 case of transient increase in blood pressure during ECT that responded to treatment, and 1 case of prolonged postictal agitation resulting in study termination. There were 4 adverse events in the MST group. These included 1 device malfunction resulting in treatment not being administered (related to a fuse that was subsequently repaired, with no further occurrences of this issue noted), 2 cases of nausea and vomiting after treatment, and 1 case of foot pain diagnosed as tendinitis unrelated to study participation.

**Figure 4.**
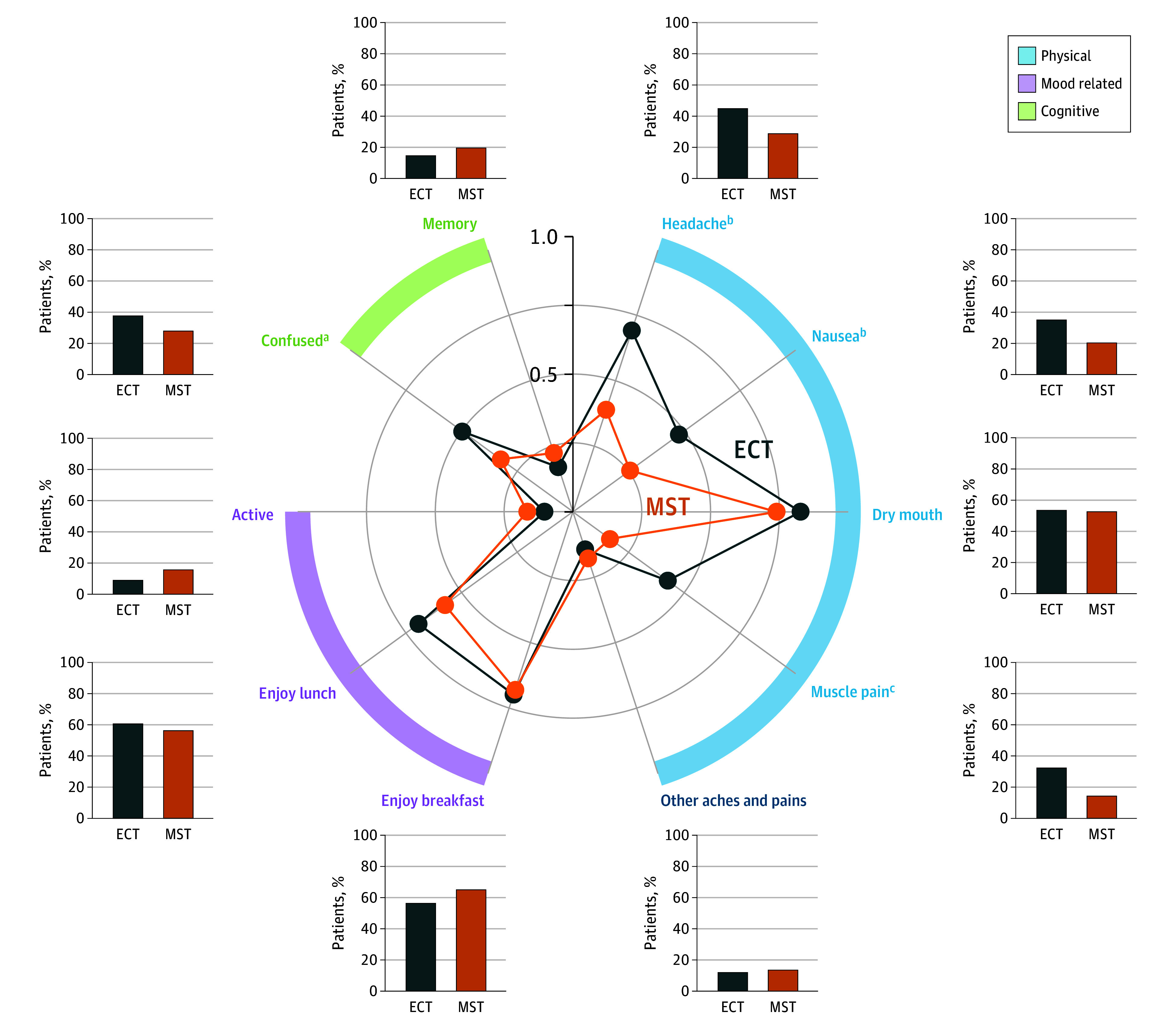
Mean Severity of Subjective Adverse Effects and Percentages of Patients Experiencing Them For physical adverse effects, patients receiving electroconvulsive therapy (ECT) reported higher severity of headache (*t*_229.9_ = 3.1; *P* = .002), nausea (*t*_225.8_ = 2.8; *P* = .006), and muscle pain (*t*_201.9_ = 3.7; *P* < .001) than patients receiving magnetic seizure therapy (MST). In the cognitive domain, patients receiving ECT reported higher severity of confusion or disorientation than those receiving MST (*t*_225.8_ = 2.2; *P* = .03). The mood-related item scores, presented as positive effects rather than adverse effects, were similar between the 2 treatment groups. ^a^*P* < .05. ^b^*P* < .01. ^c^*P* < .001.

Figure 4 shows both the mean severity scores and percentage of patients experiencing each subjective adverse effect symptom. For physical adverse effects, patients receiving ECT reported higher severity of headache (*t*_229.9_ = 3.1; *P* = .002), nausea (*t*_225.8_ = 2.8; *P* = .006), and muscle pain (*t*_201.9_ = 3.7; *P* < .001) compared with patients receiving MST. In the cognitive domain, patients receiving ECT reported higher severity of confusion or disorientation compared with patients receiving MST (*t*_225.8_ = 2.2; *P* = .03). The mood-related item scores were similar between the 2 treatment groups.

Compared with patients receiving ECT, those who received MST showed better recall of autobiographical memories following the treatment course (*t*_22.3_ = 2.8; *P* = .01) and better autobiographical memory specificity (*t*_32.6_ = 2.2; *P* = .03). There was no significant difference between the 2 groups in the change of the number of categorical memories recalled.

## Discussion

In what is, to our knowledge, the largest double-blind RCT to date (N = 73) comparing clinical outcomes of MST and ECT, both groups exhibited similar and robust antidepressant efficacy. This finding aligns with a recent meta-analysis of 4 RCTs (with sample sizes ranging from 6-40), which reported comparable effect sizes between MST and ECT at the end-point assessment and 1-month follow-up.^20^ Our study is the first, to our knowledge, to demonstrate durability of antidepressant effects of MST that were comparable with those of ECT for up to 6 months. It is worth noting that our MST technique diverges from previous RCTs as we used a circular coil (Figure 1) instead of a twin coil. These coils have distinct E-field characteristics,^5^ potentially resulting in different seizure induction efficiencies and responses. Furthermore, the ECT parameters also differ across previous RCTs. Importantly, we used ultrabrief pulse width RUL ECT at 6 times ST, which has been demonstrated to match the efficacy of bitemporal ECT,^21^ providing a strong test of relative efficacy.

Over the first half of the treatment course, MST exerted similar antidepressant action and speed of response compared with ECT. However, the mean number of treatments needed to achieve remission was 2.3 less for ECT than MST. Similar results have been found among the different ECT electrode configurations, with fewer sessions needed to achieve remission with bitemporal relative to RUL ECT.^30^ While the additional MST treatments would have milder physical and cognitive adverse effects, they would still increase the risks associated with general anesthesia. Further research is warranted to examine ways to optimize MST dosage to accelerate speed of remission.

Time to orientation after MST was on average just a few minutes (substantiating prior research^9^), while the average for ECT was significantly longer, almost 20 minutes. As longer time to orientation is a predictor of retrograde amnesia severity,^31^ this study supports the cognitive safety of MST and is consistent with previous reports on MST that found that cognitive adverse effects were negligible.^16,17^ Indeed, patients receiving MST exhibited superior performance in both autobiographical memory recall and specificity, as indicated by the Autobiographical Memory Test. Autobiographical recall is associated with impact on the temporal cortices, particularly the hippocampus^32^; it appears that ECT may have a more adverse impact on the hippocampus compared with MST. Patients also reported significantly fewer subjective adverse effects with MST, specifically having fewer and less severe physical adverse effects, such as headache, nausea, and muscle pain, and less posttreatment confusion or disorientation. The treatment frequency of seizure therapy is presently limited to 2 or 3 times weekly due to cognitive safety concerns. With a more tolerable cognitive safety profile, MST would be amendable to a more accelerated treatment schedule. Just as accelerated TMS has been shown to achieve faster depressive symptom relief with a higher remission rate compared with conventional repetitive TMS,^33^ efficacy could also improve with accelerated seizure therapy. A pilot study showed that accelerated MST was feasible and well tolerated.^34^

### Limitations

This study has several limitations. The study design did not include a placebo condition; however, the patients had a high degree of depression severity and chronicity as well as psychiatric comorbidity—clinical characteristics that tend to be associated with a low placebo response. There were technological limitations on MST dosing. ST titration for ECT was performed in steps with relatively high resolution across a wide range of levels, and, once found, doses at 6 times ST could be achieved. Despite using the most powerful MST device available at the time, titration consisted of an initial step of 5 seconds, followed by a single step up to the maximum dose permitted by the device that was subsequently used over the treatment course. For many patients, this dose was likely below 6 times ST. The progressive decrease in MST seizure duration over time suggests that the MST dose was unable to compensate for treatment-induced anticonvulsant action. Failure to compensate for increasing ST may have limited the antidepressant potential of MST. Despite these limitations, the finding that MST efficacy matched that of 6 times ST ECT is remarkable given that the efficacy of ECT is highly dose dependent. Our study was not powered as an equivalence or noninferiority trial; thus, we cannot definitively conclude that MST is noninferior to ECT. An adequately powered noninferiority trial is currently underway.^35^

## Conclusions

In this trial, MST had similar efficacy relative to ultrabrief pulse RUL ECT at 6 times ST, with sustained benefit up to 6 months. Moreover, the faster time to orientation and improved autobiographical memory recall found with MST suggest that MST may provide substantial antidepressant benefits while maintaining a high level of cognitive safety. Our results indicate a need to further optimize MST dosing to compensate for treatment-induced elevation in ST.

## Notes

Supplement 1. Trial Protocol

Supplement 2. **eFigure 1.** Inventory of Depressive Symptomatology (IDS) **eFigure 2.** Seizure Duration and Time to Orientation

Supplement 3. Data Sharing Statement
