# Supplementary material for: Clinical Outcomes of Magnetic Seizure Therapy vs Electroconvulsive Therapy for Major Depressive Episode: A Randomized Clinical Trial
Source: JAMA Psychiatry. 2023 Dec 6;81(3):240–9. doi: 10.1001/jamapsychiatry.2023.4599 (PMC10701670; doi:10.1001/jamapsychiatry.2023.4599)
Supplement: Supplement 1. — Trial Protocol [file jamapsychiatry-e234599-s001.pdf]

FDA
ORIGINAL INVESTIGATIONAL DEVICE EXEMPTION APPLICATION

DEVICE: MAGSTIM THETA REPETITIVE MAGNETIC STIMULATOR

STUDY TITLE: MAGNETIC SEIZURE THERAPY (MST) FOR THE TREATMENT OF SEVERE MOOD DISORDER

Device Information:
Magstim Theta Repetitive Magnetic Stimulator
Intended use: administration of magnetic seizure therapy

Sponsor: Department of Psychiatry & Behavioral Sciences
Box 3950
Duke University Medical Center
Durham, NC 27710
Phone: 919-684-5616
Fax: 919-681-5489

Manufacturer:
Magstim Company Limited
Whitland, Dyfed SA34 0HR
Wales, United Kingdom
Contact: Dr. Reza Jalinous
Phone: 888-624-7856

Applicant:
Dr. Sarah H. Lisanby
Chair, Department of Psychiatry & Behavioral Sciences
Box 3950
Duke University Medical Center
Durham, NC 27710
Phone: 919-684-5616
Fax: 919-681-5489

Date of Submission: 12/30/10

Submission to:
Food and Drug Administration
Center for Devices and Radiological Health
Document Mail Center, HFZ-401
9200 Corporate Boulevard
Rockville, MD 20850

Glossary of Abbreviations

BL=bilateral electroconvulsive therapy
CMR=cerebral metabolic rate
CU=Columbia University
DUMC=Duke University Medical Center.
DU= Duke University
ECT=electroconvulsive therapy
EEG=electroencephalography
MDE=major depressive episode
MST=magnetic seizure therapy
NYSPI=New York State Psychiatric Institute
rCBF=regional cerebral blood flow
rTMS=repetitive transcranial magnetic stimulation
RUL=right unilateral electroconvulsive therapy
TMS=transcranial magnetic stimulation
UTSW=University of Texas Southwestern

MAGNETIC SEIZURE THERAPY FOR THE TREATMENT OF SEVERE MOOD DISORDER

I. INTRODUCTORY STATEMENT

This original investigator-initiated Investigational Device Exemption (IDE) application is submitted for the purpose of conducting a research study to test the safety and efficacy of using repetitive transcranial magnetic stimulation (rTMS) as an alternative to electroconvulsive therapy (ECT) in the induction of generalized seizures for therapeutic purposes. The study will be conducted in psychiatric patients with a major depressive episode (MDE) referred for convulsive therapy. For the purposes of this application, we will refer to the use of rTMS to intentionally induce seizures as "Magnetic Seizure Therapy" or "MST" to distinguish it from the subconvulsive use of rTMS.

The application is for a two-center study of 75 patients to be conducted at Duke University / Duke University Medical Center (DU/DUMC), Durham, NC and the University of Texas Southwestern, Dallas, Texas. Duke University will replace the CU/NYSPI site which will continue to follow patients previously enrolled. No more patients will be enrolled at the CU/NYSPI site. The device that will be used is the Magstim Theta repetitive magnetic stimulator, manufactured by the Magstim Company Limited. This is an investigator-initiated application. The investigator intends to use the findings of this study to compare the efficacy and safety of MST with conventional ECT in the treatment of depression. Manufacturing information has been supplied by the Magstim Company Limited, and is included in this application.

Electroconvulsive therapy (ECT) is the most effective treatment available for major depression. Yet, cognitive side effects limit its use and a significant number of patients do not respond. Electrical dosage and current paths are critical to the efficacy and side effects of ECT. To ensure efficacy and limit side effects, clinicians need both better control over current density and greater specificity in the brain regions targeted. However, the impedance of the skull and scalp shunt the electrical stimulus, resulting in poor control over the strength and distribution of stimulation and variability both between and within patients.

Repetitive transcranial magnetic stimulation (rTMS) avoids these pitfalls by inducing current noninvasively, using rapidly alternating magnetic fields. The scalp and skull are transparent to magnetic stimulation and the magnetic field can be spatially targeted. Thus, magnetic stimulation offers more precise control over charge density and current paths in neural tissue. Recent technical advances and important new data in animals and human patients support the feasibility of Magnetic Seizure Therapy as a novel convulsive technique. Given its superior control over dosing and spatial distribution, MST offers the promise of fewer side effects and possibly improved efficacy.

The purpose of this between-subject study will be to determine whether seizure induction with MST is effective in treating Severe Mood Disorder, and to compare MST with conventional ECT in terms of efficacy and side effect profile.

Procedures in this IDE are similar to those in our prior IDEs on MST (G000185 and G020028). We hereby grant FDA permission to access information in our IDEs No. G000185 and G020028 in its review of this IDE.

II. REPORT OF PRIOR INVESTIGATIONS

II. A. The Public Health Impact of Depression

Depression is a leading public health problem contributing to substantial morbidity and mortality. Depression is life-shortening due both to suicide and the effect of depression on increasing the mortality associated with various general medical conditions. Major depression affects ~14 million American adults each year[1] and is one of the leading causes of disability/burden of illness[2] worldwide. The WHO estimates that at current rates, by 2020 major depression will be the second most common cause of disability worldwide.[3]

II. B. The Unmet Clinical Need of Treatment Resistant Depression

Treatment resistant depression (TRD) has received increased attention in recent years with the recognition that antidepressants fail to effectively treat a substantial proportion of patients. Effective pharmacological treatments are available, but a sizeable percentage of patients (estimates range from 30-40%) fail to respond to antidepressant medications. Given the high prevalence and morbidity associated with depression, it is particularly disturbing that response rates to the first antidepressant can be as low as 50%.[4] As found in the Sequenced Treatment Alternatives for Depression (STAR*D) study that evaluated outpatient, community based, non-psychotic depressed patients, referred from general clinical and psychiatry clinics, only 28%-33% of patients achieved remission with an initial antidepressant medication.[5] Subsequent antidepressant treatment by either augmentation or switching pharmacotherapeutic agents resulted in remission rates of 30.6%, 13.7%, and 13.0% , for the second, third, and fourth steps respectively.[6] Thus, newer forms of antidepressant treatments are needed to help treat treatment-refractory patients.

II.C. The Continued Role of ECT in the Treatment of Depression

ECT remains the mainstay of treatment for TRD because it is the most effective and rapidly acting treatment for severe depression.[7] Vagus Nerve Stimulation (VNS) was approved for the adjunctive treatment of chronic TRD; however, response rates with VNS (30% after 1 year) fall short of those typically seen with ECT (65-85%). Thus, there remains a compelling clinical need to continue administering ECT.

It is estimated that 1-2 million individuals receive ECT each year worldwide, with utilization increasing. ECT is rapidly effective in reversing suicidality, the greatest source of morbidity and mortality from depression. ECT plays an important role in the treatment of severe depression and both the depressed and manic phases of bipolar disorder, especially for patients who cannot tolerate or who have not responded to psychotropic medications. Because of its exceptionally high response rate (especially in psychotic depression), rapid onset of action, and profound acute beneficial effect on suicidality, ECT is uniquely suited for the treatment of the most severely ill depressed and psychotic patients. ECT also has an emerging role in treatment resistant schizophrenia, demonstrating synergism in combination with neuroleptic medication and it may reduce long-term extrapyramidal side effects. If we had a treatment with the efficacy of ECT but without its cognitive side effects discussed below, its usage could be even higher than the estimated 100,000 patients/year who receive ECT in the US.

II.D. The Need to Improve the Tolerability of Seizure Therapy

Although ECT is the most effective somatic treatment for major depression, its cognitive side effects substantially reduce its tolerability. [7] Retrograde amnesia is the most persistent adverse effect of ECT.[8] Shortly after ECT, most patients have gaps in memory for events that occurred close in time to ECT, but retrograde amnesia may extend several months or years. While retrograde amnesia often improves during the first few months following ECT, for many patients recovery is incomplete, with prolonged amnesia for events that occurred close to the time of treatment. A recent report found, in a large sample of patients treated in community settings, that specific cognitive deficits persisted at least 6 months.[9] This represents the first clear-cut documentation of persistent retrograde amnesia post ECT in a large sample. These results underscore the fact that strategies for reducing the cognitive side effects of ECT are greatly needed and would have a significant public health impact. Reducing the side effects of ECT would represent a special benefit for the elderly, who are especially vulnerable to the amnestic effects of ECT. Indeed, pre-existing cognitive impairment is one of the few predictors of ECT's adverse effects, and is more likely with advancing age.[10]

Modifications in ECT technique have substantially reduced, but they have not resolved, the problem of cognitive side effects. This may be because the use of externally applied electrodes intrinsically limits the capacity to control the intracerebral spatial distribution of the ECT stimulus and its intracerebral current density. Regardless of the modification of ECT technique, the possibility of marked and/or persistent cognitive side effects remains a problem.

The present protocol aims to capitalize on the knowledge acquired in ECT research to evaluate a more focal form of convulsive therapy, MST, that to date appears to have minimal cognitive effects[11] and significant antidepressant properties.[12]

II.E. Rationale for Magnetic Seizure Therapy (MST)

The rationale for MST is reviewed in detail in the attached manuscripts.[11-22] Briefly, the efficacy and side effects of ECT are largely determined by the site of seizure initiation and by the patterns of seizure spread. Neither factor can be adequately controlled with ECT techniques currently in use. Both parameters can be controlled with MST, which has the potential of retaining the efficacy of ECT while having a markedly better side effect profile.

Both MST and ECT induce seizures through electrical stimulation of the brain. In the case of ECT, the electricity is applied directly to the scalp (transcranially), whereas with MST the electricity is indirectly induced in the brain by a magnetic stimulus. With ECT, the high impedance of the skull shunts most (80-95%) of the electrical stimulus away from the brain.[23] A minor portion of the electrical stimulus causes neuronal depolarization, with the shunting producing a nonfocal, widespread, intracerebral current distribution.[24] The topography of shunting varies considerably among individuals, due to variation in skull thickness and anatomy. These individual differences result in regional variability in current density even when electrode placement is consistent. Thus, an externally applied electrical stimulus that must cross the scalp and skull to reach the brain will necessarily result in variable and widespread current distribution. Yet, there is evidence that the anatomic distribution and intensity of current density are critical in determining the efficacy and side effects of ECT.[25-29] MST avoids these limitations by generating magnetic fields that pass through tissue without impedance.[30]

MST offers precise control over the site of seizure initiation and the capacity to limit seizure spread. The electrical field induced by MST is capable of neural depolarization at a depth of about 2 cm below the scalp (i.e., gray-white matter junction), so direct effects of stimulation are limited to the superficial cortex.[31-34] Coil geometry allows the magnetic field to be spatially targeted to specific cortical regions, offering further control over intracerebral current paths. Measurements in nonhuman primates with intracerebral multicontact electrodes support the hypothesis that MST-induced current and the resulting seizure are more focal than that obtained with ECT.[17] This enhanced control represents a means to focus the treatment in targeted cortical structures thought to mediate antidepressant effects and to reduce spread to medial temporal structures implicated in the amnestic side effects of ECT.

Targeting the site of seizure onset is critical because the physiological consequences of seizures, as reflected in the characteristic and marked interictal reductions in cerebral metabolism and blood flow, and in the increase in electrical slow wave activity, are more marked at sites of seizure initiation than seizure propagation.[29, 35, 36] For both ECT and secondarily generalized focal seizures in epilepsy, the surrounding inhibition following seizures is greatest at sites of seizure initiation. Despite inducing a bilaterally generalized seizure, RUL ECT results in material-specific anterograde amnesia and other behavioral patterns compatible with greater dysfunction of the hemisphere ipsilateral to stimulation. Likewise, there is marked asymmetry in the physiological alterations following unilateral ECT, with prominent effects restricted to the side of stimulation.[28]

II. F. Prior Studies with MST

Our work in nonhuman primates demonstrated the safety of MST and electroconvulsive shock (ECS) (using neuroanatomical, neuropathological, and stereological measures), showed that the interventions differed in neurophysiological and neuroanatomical measures that may relate to their differing cognitive profiles, and provided new data on the hippocampal neural plasticity in response to these interventions.[17, 18] The electric field induced by MST is less intense and more confined to superficial cortex than ECS. MST-induced seizures show less robust ictal expression, less postictal suppression, less generalization to hippocampus and deeper brain structures, and results in less robust serum prolactin surge and less immediate post-stimulus bradycardia. These marked physiological differences are consistent with MST having less of an impact than ECS on temporal lobe and diencephalic structures, as hypothesized, and with less impact on parasympathetic outflow that should lower the risk of cardiac complications. We found that ECS, but not MST, induces mossy fiber sprouting (MFS) and cellular proliferation in the dentate gyrus.[37, 38] Lack of physiological and structural changes in the hippocampus with MST may relate to its superior cognitive profile.[11] Indeed, our nonhuman primate model of the amnestic side effects of ECT revealed that monkeys were more accurate and faster following MST as compared to ECS.[19]

To date, 47 patients have received MST worldwide since the first patient was treated in 2000. In the context of proof of concept case study, the 1st patient had a 50% drop in Hamilton Depression Ratings Scale ($HRSD_{24}$) scores following 4 MST sessions.[13] MST was well tolerated with no significant side effects. A second patient with medication resistant depression was treated with a longer MST course (12 treatments) and experienced remission, with an 82% drop in $HRSD_{24}$ and a final $HRSD_{24}$=6.[15]

Our double-masked, randomized, within-subject trial contrasting the acute cognitive side effects of MST with ultrabrief pulse RUL ECT (the form of ECT with the fewest cognitive side effects) found MST was well tolerated with fewer side effects than ECT and faster recovery of orientation (Fig. 1), a measure that predicts the magnitude of long-term retrograde amnesia.[11] Masked neuropsychological assessments revealed advantages of MST relative to ECT. Consistent with the differential impact of MST and ECT on seizure expression and hippocampal synaptic plasticity, the cognitive domains where ECT showed greater impairment than MST were those subserved at least partly by temporal lobe structures (i.e., memory for recent events, new list learning, category fluency). In contrast, MST and ECT did not differ on tasks more heavily dependent on prefrontal lobe function (i.e., memory for temporal order, verbal fluency), consistent with the view that MST would retain effects on prefrontal structures important for efficacy.

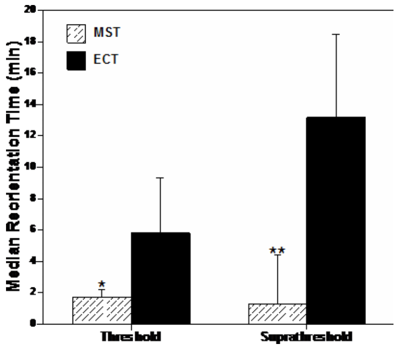

Fig. 1 Reorientation faster following MST than ECT. P's<0.004.

Our randomized, double-masked, 2-center [NYSPI & UTSW] study compared two forms of MST in their antidepressant properties and side effects.[12] Twenty medication resistant patients referred for ECT (age 46.7 ± 9.9 yrs, 40% women, baseline $HRSD_{24}$ =33.5 ± 5.4) with a baseline $HRSD_{24} \geq 18$ and SCID-I diagnosis of MDE (95% unipolar, 15% psychotic subtype) completed a course of MST following medication washout.

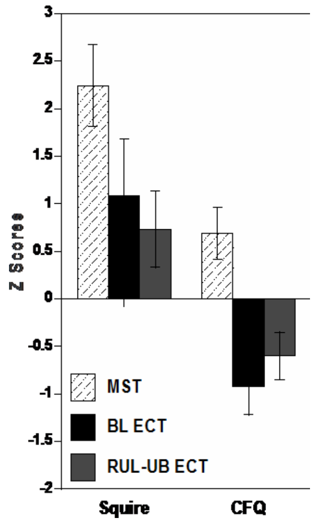

Fig. 2 Better memory profile following MST than ECT.

Safety: Masked neuropsychological batteries were performed at baseline, postMST, and at 2 months postMST. Results were z-transformed relative to the distribution of baseline scores, and compared to a large NIMH funded study of ECT in the community setting (n=347), and to patients receiving state of the art ECT in the context of an NIMH sponsored randomized trial of 4 different forms of ECT (n=77). Measures of subjective memory function (Squire and Cognitive Failures Questionnaire, CFQ) were better following MST than ECT (Fig. 3, p's<0.02). Benefits persisted at 2 months (p's<0.004). MST resulted in less global cognitive impairment, anterograde amnesia, and retrograde amnesia (Autobiographical Memory Inventory, AMI) than the community ECT sample (p's<0.006) and the academic ECT setting (Fig. 4). The advantages of MST relative to ECT in AMI persisted at follow up (p<0.05).

Efficacy: After 9.0 ± 2.8 MSTs, significant clinical improvement was seen on the $HRSD_{24}$ (mean improvement=48.7 ± 28.0%, BDI drop=37.5 ± 35.3%, p's<0.0001; Fig. 5). This is substantial and in the same range

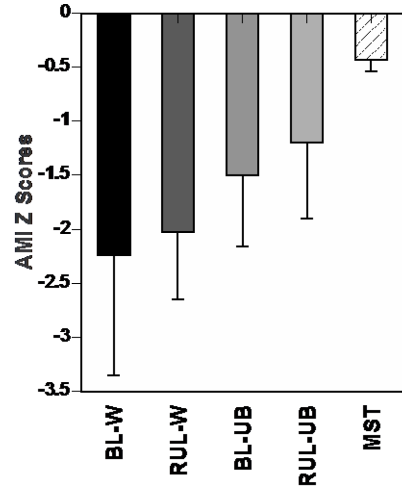

Fig. 3 Less amnesia for autobiographical events less following MST than 4 forms of ECT.

or better than that seen in some drug trials. Speed of improvement was similar to ECT. 60% of patients showed at least a 50% drop in $HRSD_{24}$, and 70% had a partial response ($\geq$30% drop). Using strict criteria ($\geq$60% drop in $HRSD_{24}$ and final $HRSD_{24}$<10), the remission rate with MST was 35%. 45% of patients were substantially improved (at least 50% from baseline) at 2 weeks postMST, providing evidence of sustained benefit. These effects are remarkable considering that the group was highly medication resistant (84%), with a high degree of chronicity (current episode=6 ± 8 yrs), and psychiatric comorbidity (75% had comorbid axis I and 15% had axis II disorders).

Efficacy of MST matched that of ECT in the community sample. But, ECT in the community setting is typically terminated prematurely and does not match the benefits of optimal ECT practice, where the group mean $HRSD_{24}$ post treatment is typically close to 10, as opposed to near 15 postMST. The higher output MST device in the current study should enhance efficacy.

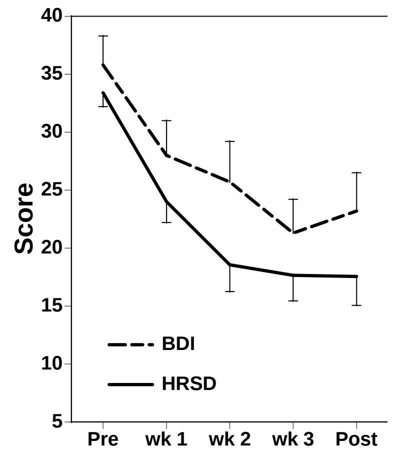

Fig. 4 Significant improvement in depression scores seen as early as first week. p's<0.001.

II. G. Prior Studies with the Magstim Theta

The Magstim Theta MST device was specifically designed to deliver a stimulation range that is expected to enhance the efficacy of MST. By extending the output range to be able to deliver 100 Hz trains at 100% maximal stimulator output for up to 10 seconds, this device is expected to be able to more effectively treat patients with higher seizure thresholds, and to induce seizures possessing topographical qualities associated with enhanced efficacy. Our prior work described above suggested that higher output was necessary to permit stimulation at high enough doses above seizure threshold to ensure antidepressant efficacy.

Following extensive performance testing at the Magstim Company described in section IV, a prototype Theta device was shipped to CU/NYSPI for animal testing in rhesus monkeys. The prototype has succeeded in inducing high dose suprathreshold seizures in a total of 229 seizure induction sessions in 9 monkeys to date (mean age 6.5 ± 2.7 years). There have been no incidents of device output failure or malfunction in these cases. Coil performance during these seizure induction sessions has been within expected parameters, with no cases of excessive heating. Seizure threshold titrations have been successfully performed allowing 30 seconds between successive stimulations. All procedures were well tolerated with no adverse events.

As part of IRB approved protocols, two Theta devices have been installed and tested by our collaborators in the UK (Dr. George Kirov at Cardiff University in Wales, UK, and Dr. Klaus Ebeiemer at the Royal Edinburgh Hospital in Scotland, UK). Drs. Lisanby and Husain traveled to the sites in Wales and Scotland to train the MST research teams and to supervise the first 3 cases. To date, 6 severely depressed patients referred for convulsive therapy have undergone MST administered with the Magstim Theta device. Of the 6 patients, 4 were female and the mean age was 40 ± 12 years. Anesthesia was identical to the standard used at these centers for clinical ECT (etomidate for sedation and succinylcholine for muscle relaxation). Five of the patients received a single MST session, and one patient was treated with MST on 2 separate days, for a total of 7 seizure inductions in the sample of 6 patients.

Seizures were induced in all patients. A representative scalp EEG recording from an MST-induced seizure is presented in Fig. 5. Average seizure duration was 80 ± 36 seconds. Treatments were well tolerated. The speed of reorientation immediately following the MST session was recorded using published methods.[11] The average time to regain full orientation following the MST session was 385 ± 162 seconds. All of these patients also received ECT, either prior to the MST session (5/6 patients) or following MST (1/6 patients) in the past. We therefore selected the ECT treatment closest in time to the MST session and compared recovery time with this matched ECT session. The average recovery time with ECT in these patients was 1148 ± 97 seconds, which is nearly 4-fold longer than recovery following MST. Paired t test revealed that recovery time post ECT was significantly longer than with MST ($t(5)=7.4$, $p<0.0007$). Results from these first cases were accepted for presentation at the 2007 annual meeting of the Society of Biological Psychiatry.[39]

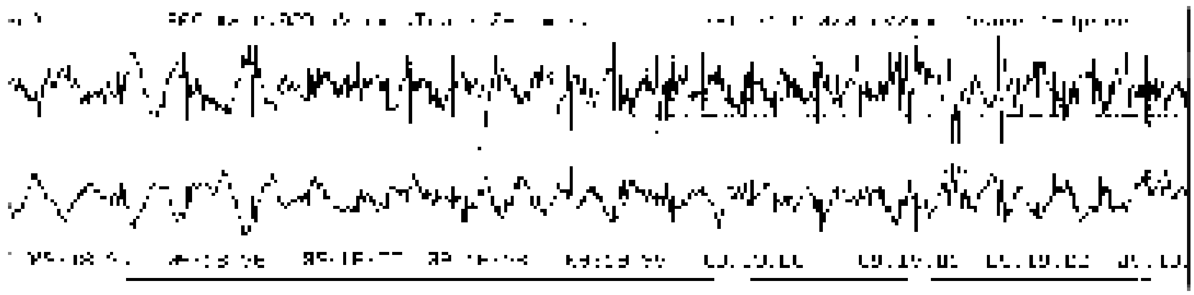

Fig. 5 Scalp EEG recording of an MST-induced seizure using the Magstim Theta device in a patient with depression.

III. INVESTIGATIONAL PLAN

III.A Study Design

This is a 2-center randomized controlled trial of MST in the treatment of depression. Over 3-years, 75 patients will enter the study. Phase 1 is a double-masked, randomized, controlled trial that contrasts the efficacy and safety of MST (vertex MST at maximal output), and ECT (Right Unilateral, 6x the seizure threshold). Eligible patients will be randomized 1:1. The blind will be maintained until the last patient has completed the study. Safety and adverse events will be assessed using standardized scales, as well as pre- and post-treatment testing of cognition. The key aspect of the trial is the Randomized Phase 1 component. Phase 2, the follow up, will provide a descriptive within-subject characterization of longer-term outcomes (i.e., long-term durability of benefit and/or persistence of side effects). The first two patients enrolled at each site will receive open-label MST to facilitate establishing consistency in procedures between sites.

III.B Hypotheses/Specific Aims

The goal of this project is to develop MST as a treatment for severe mood disorder with fewer cognitive side effects than ECT, the most effective treatment for severe depression. Unlike ECT, MST can initiate seizures in superficial cortex, while limiting impact on deeper medial temporal structures thereby resulting in less cognitive side effects. Results support the safety of MST and provide open-label evidence of antidepressant action. This randomized, double-masked, controlled clinical trial of MST in depression will test the antidepressant efficacy of high dose vertex MST.

Aim 1. Contrast the Antidepressant Efficacy of MST versus ECT

Depressed patients will be randomly assigned to receive MST (high dose vertex placement) or ECT (6x threshold ultrabrief pulse right unilateral, RUL). High dose vertex MST was selected as the MST condition because: (1) the high reliability of producing tonic-clonic seizure activity at the vertex placement, and (2) dosage is a major predictor of antidepressant response with ECT, especially for unilateral and ultrabrief pulse ECT. The maximal dosing strategy increases the likelihood of efficacy and permits secondary analyses examining relations among dosage relative to seizure threshold (ST) and clinical outcome. We will test the following hypotheses:

a. *MST and ECT will have similar antidepressant efficacy.* The primary outcome measure will be the Hamilton Rating Scale for Depression ($HRSD_{24}$) in the intent-to-treat sample. Response and remission status will provide secondary outcome measures to evaluate clinical significance.
b. *In the MST group, higher dosage relative to ST will predict greater antidepressant response.* Dosage will have a linear relationship with the primary continuous measures and the secondary categorical outcomes.

Aim 2. Contrast the Cognitive Side Effects of MST and ECT

Our pilot work suggests the cognitive side effects of MST are less marked than ECT. It has been repeatedly shown that the antidepressant effects of ECT are independent of its cognitive side effects, and imaging reveals distinct circuitry subserving the antidepressant and amnestic effects of ECT. It will be important to explore whether that same dissociation holds for MST. The side effects of ECT are sensitive to dose, waveform, site of stimulation,

and time of assessment. In the acute postictal period and for a few days following the course of ECT (subacute period), higher dosage relative to ST is associated with more severe cognitive deficits. We would expect the same to hold for MST. We hypothesize:

a. ECT and MST groups will differ in extent of post-treatment amnesia as reflected in primary measures of anterograde and retrograde amnesia following the acute treatment phase. Multivariate analyses of covariance on post treatment scores standardized to the sample's baseline performance will yield a main effect of condition, attributable to the advantage of MST vs ECT.
b. At follow up, ECT and MST will differ in degree of persisting deficit in measures of retrograde amnesia.

III.C Rationale and Significance

The history of ECT has been marked by a series of technical improvements that have helped to reduce side effects. The first of these was the shift from sine wave to brief pulse ECT. The second was the introduction of threshold titration and the realization that electrode placement and dosage relative to threshold interact in determining efficacy and side effects. The next stage we are currently investigating in the improvement of ECT involves moving beyond the direct application of electricity, due to the inherent limitations posed by the impedance of the scalp and skull. Rapid Transcranial Magnetic Stimulation (rTMS) offers the ability to transcend these limitations since magnetic fields pass through biological tissue as if they were transparent. Magnetic seizure induction offers the potential of enhanced control over location and degree of stimulation, factors that are key in determining the efficacy and side effects of ECT. Focused seizure induction could potentially target prefrontal circuitry involved in mood, and limit spread to medial temporal lobe and other regions thought to be critical to the memory side effects of ECT.

Our recent work (summarized above in section XIII) has demonstrated the feasibility of MST in animals and patients with depression. The results of our first clinical trial with MST indicated that it has a markedly milder acute cognitive side effect profile than ECT and patient acceptance of the treatment was high. Results from this study led to the investigation of a two-site trial assessing the safety and efficacy of a full-course of MST. That study assessed two types of MST, focal, with frontal coil placement using a double-cone coil, and non-focal, with vertex placement using a cap coil. The results indicated that MST has antidepressant efficacy with limited cognitive and adverse side effects. Overall, 60% of the total sample (N=20) responded to MST and 35% remitted, with an average of 9 MST treatments. Further, due to dosage limitations with the prior device, MST with prefrontal placement was unable to reliably produce consistent tonic-clonic seizure activity in all patients due to increased seizure threshold post initial treatment. The finding of increased seizure threshold is consistent with the neurophysiological effects seen with subsequent ECT treatments that provided further evidence for the efficacy of MST. The Magstim Theta now provides reliable seizure induction (see Section VIII.G. for preliminary studies with the Magstim Theta) with an expanded output range that will enable a more definitive test of the antidepressant efficacy of MST. The proposed study will investigate the antidepressant efficacy and side effects of a course of MST delivered with the Magstim Theta compared to ECT.

This work has the potential to reshape ECT by moving beyond the direct application of electricity, with its inherent limitations in focality and precision of stimulation. Consequently, this study addresses an issue of considerable public health concern by

determining whether MST merits further exploration as a means of reducing the cognitive and adverse side effects of ECT in the treatment of severe depression.

III.D Subject Selection

Patients in a major depressive episode in the context of unipolar or bipolar disorder will be invited to participate. Patients are referred by private physicians and by clinical services at DUMC, UTSW, and other psychiatric facilities.

Patients with conditions contraindicating the use of MST will be excluded (see inclusion/exclusion criteria below). There is no evidence that gender and ethnic status are related to response to MST. Given the gender difference in the prevalence of major depression, it is expected that approximately 2/3 of the sample will be female. Expected ethnic breakdown is 80% Caucasian, 10% African-American, 10% Hispanic or other ethnic group.

III.D.1 Screening

To be eligible, patients must have a clinical diagnosis of a major depressive episode (in the context of unipolar or bipolar disorder) and appropriate indications. Patients who meet the inclusion/exclusion criteria and for whom convulsive therapy is indicated will be offered participation and informed consent will be obtained. Medical workup will include history, physical, and neurological exam, and standard preECT lab workup as clinically indicated. In women of childbearing age, a pregnancy blood test will be obtained.

III.D.2 Inclusion/Exclusion Criteria

Inclusion and exclusion criteria are provided in Table 1.

Table 1. Inclusion and Exclusion Criteria

**INCLUSION CRITERION**:	**METHOD OF ASCERTAINMENT**
1. Age 18-90	Self-report
2. Clinical diagnosis of major depressive episode, in the context of unipolar or bipolar disorder	Structured Clinical Interview for Diagnosis (SCID-I)
3. Use of effective method of birth control (including abstinence) for women of child-bearing capacity	Physician evaluation (Transcranial magnetic stimulation Adult Safety Screen (TASS)
4. Willing and capable to provide informed consent	Physician evaluation
5. Convulsive therapy clinically indicated	Physician evaluation
6. Hamilton Rating Scale for Depression ($HRSD_{24}$) ≥ 20	$HRSD_{24}$

**EXCLUSION CRITERION**:	**METHOD OF ASCERTAINMENT**
1. Current unstable or serious medical condition, or any comorbid medical condition that	Physical evaluation (physical and neurological examination, EKG, blood and urine analysis)

substantially increases the risks of ECT (such as acute myocardial infarction, space occupying brain lesion or other cause of increased intracranial pressure, unstable aneurysm or vascular malformation, poorly controlled diabetes mellitus, carcinoma, renal failure, hepatic failure)	
2. Pregnancy	Serum pregnancy test (β-HCG) in women of childbearing capacity
3. History of neurological disorder, epilepsy, stroke, brain surgery, metal in the head, history of known structural brain lesion	Physician evaluation (Transcranial magnetic stimulation Adult Safety Screen (TASS), medical history and neurological examination)
4. Presence of devices that may be affected by MST (e.g., pacemaker, medication pump, cochlear implant, Vagus Nerve Stimulation, Deep Brain Stimulation, implanted brain stimulator)	Physician evaluation (Transcranial magnetic stimulation Adult Safety Screen (TASS), medical history)
5. Breast-feeding	Self report
6. History of head trauma with loss of consciousness for greater than 5 minutes	Physician evaluation (medical history)
7. History of schizophrenia, schizoaffective disorder, or rapid cycling bipolar disorder	Structured Clinical Interview for Diagnosis (SCID-I)
8. History of substance abuse or dependence in past 3 months	Structure Clinical Interview for Diagnosis (SCID-I), urine toxicology screen
9. History of ECT in the past 6 months and/or failure to respond to an adequate trial of ECT lifetime	Physician evaluation (history) and Antidepressant Treatment History Form (ATHF)
10. Patients for whom withdrawal of psychotropic medications would be clinically inadvisable	Physician evaluation (history)
11. Presence of intracardiac lines	Physician evaluation (history)

III.E Medication

Antidepressant medications (except for PRN lorazepam up to 3 mg/day) will be tapered and washed out for at least 3 days prior to the start of treatment. Patients will be monitored on the inpatient unit during the washout period via clinical interviews. The taper schedule will be tailored to each patient according to their individual medications and clinical status. While the half-life of some medications will be longer, the severity of patients referred for ECT necessitates a shorter washout period for clinical reasons. Medications for medical conditions will be administered as clinically indicated and as ordered by the patient's treatment team. As is standard practice in the DUMC and UTSW ECT service, and as recommended by the APA Task Force Report on ECT, benzodiazepines are withheld for 10 hours prior to the treatment.

III.F Timeline of the study

Table 2. Timeline of Major Study Procedures.

	MST/ECT Course	Follow

		(3/wk)						up	
**Assessments**	**Baseline**	**1st**	**2nd**	**	**Next to last**	**Last**	**Post-MST /ECT**	**2 mo**	**6 mo**
Baseline Screening	x								
Neuropsychological Testing	x	x	x	x	x	x	x	x	x
Clinical Ratings	x	x	x	x	x	x	x	x	x
Other Procedures:									
Motor Cortex Excitability	x						x		
EEG	x	x	x	x	x	x	x		
Biochemical studies		x				x			

** represents variable number of MST sessions given 3 per week
Mo=month

Table 2 presents the timeline of major study procedures. Baseline assessments include the determination of eligibility, medical screening, and standard laboratory workup recommended for patients referred to ECT. Neuropsychological batteries will be administered to assess the cognitive effects of the treatments at baseline, following every treatment, at the end of the treatment course, and at 2 month and 6-month follow-up to examine long-term effects. Clinical ratings are performed at baseline, thrice a week during the treatment course, at the end of the course, at 2 month follow-up, and at 6 month follow-up to examine antidepressant effects of convulsive treatment. Neurophysiological studies (motor cortex excitability studies and EEG) and biochemical studies will be performed at baseline, selected time-points during the course, and at the end of the course to collect data on the neurophysiological and neuroendocrinological characteristics of MST and ECT seizures. Treatment will be administered three times per week. There will be no maximum number of sessions; the number of treatments will be based on the treating physician's clinical assessment. It is expected that most patients will receive treatment for two to six weeks (six to 18 sessions).

III.G Anesthesia and Monitoring

MST and ECT will be performed under general anesthesia of the type used in standard clinical ECT, and the same methods that were employed in IDE # G000185 and IDE# G020028. Anesthesia will be administered by anesthesiologists with specific training and experience in ECT. Atropine (0.4 mg i.v.) will be given two minutes prior to anesthesia induction. Etomidate (0.15-0.2 mg/kg) or methohexital (0.5-0.75 mg/kg), and succinylcholine (0.75-1.0 mg/kg) will be used as the intravenous anesthetic agents. Patients will be oxygenated from anesthetic administration until return of spontaneous respirations. Seizure duration will be monitored with two frontal-mastoid EEG channels, as well as motor manifestations using the cuff technique. The cuff technique entails the placement of a blood pressure cuff on a limb. The cuff is then inflated above the systolic blood pressure

immediately prior to the infusion of succinylcholine. This maneuver prevents the limb from being exposed to the paralytic effects of succinylcholine so that a motor seizure will be observed in that limb, and can be timed. The succinylcholine will block the expression of a motor seizure in the rest of the body, thereby reducing risk of injury to the extremities that can result from an unmodified tonic-clonic seizure. Using conservative criteria (≥20 seconds), generalized seizures of adequate duration will be elicited at each treatment.

III.H Magnetic Seizure Therapy (MST)/Electroconvulsive Therapy (ECT)

MST will be performed under general anesthesia (as outlined above) at each respective site's (DUMC and UTSW) ECT suite using a custom MST device (Magstim Theta) capable of stimulation at 100 Hz, 100% intensity, for 10 seconds. ECT will be performed under general anesthesia (as outlined above) at each respective site ECT suite using a Thymatron or MECTA Spectrum 5000Q capable of delivering right-unilateral, ultrabrief pulse stimulation, at six-times the seizure threshold. Each ECT suite is staffed by personnel trained in the acute care of patients undergoing convulsive therapy, and in the prompt recognition and treatment of potential post-ictal complications. Also, each ECT suite is fully equipped to manage potential medical emergencies. This emergency equipment includes oxygen supply, IV line supplies, emergency medications, and a crash cart.

Patients and experimenters will wear earplugs to protect inner and middle ear structures from the potential acoustic trauma of the magnetic coil stimulation artifact and thus prevent the risk of transient auditory threshold shifts.

On the first and last session, seizure threshold (ST) will be determined by the ascending method of limits procedure. Threshold determination entails the repeated application of increasingly powerful stimuli until a seizure is obtained. A single train is given at each step of the titration. The starting parameters for the titration will be 100 Hz, and 100% maximal stimulator output. At each step in the titration, the duration of the train will be lengthened (up to 10 seconds) until a seizure is obtained. At least 20 seconds will be allowed between successive stimuli, as is standard practice with ECT, to monitor for delayed onset seizures.

For those patients assigned to MST, subsequent treatments will be given at maximal stimulator output over vertex. We selected stimulation at maximal stimulator output for the vertex placement to maximize likelihood of antidepressant response. The ECT literature has shown that higher dosages above threshold are required to achieve maximal antidepressant response. This is the first study to test an MST device capable of stimulating at substantially above the seizure threshold. Since there are no prior studies to guide the selection of what dosage above threshold would be optimal with MST, we have decided to stimulate all patients in the vertex condition at the maximal stimulator output. That choice should maximize antidepressant response and will also guide the selection of the optimal dosage for subsequent studies because individual patients will differ in their seizure threshold. Thus, using a fixed dosage of MST will result in patients receiving a range of dosages above their seizure thresholds. Post hoc analyses will test whether dosage above threshold was predictive of response. For those patients assigned to ECT, right unilateral ECT will be administered at 6xST using an ultrabrief stimulus (0.3 ms). Clinical work at NYSPI has shown that this form of ECT provides the best cognitive profile without sacrificing efficacy.

If MST fails to induce a seizure: To date, we have been successful in inducing seizures with vertex placement in all humans and animals that have undergone the procedure. Therefore, we do not expect it to be a common occurrence for MST not to induce a seizure.

However, if a patient fails to have a seizure during any MST session they will be dropped from the randomized portion of the study and offered routine clinical care. Ultimately, the decision as to what is the best next treatment is a clinical one to be made by the patient in consultation with the treatment team.

If a patient has an aborted seizure (< 20 seconds) with either MST or ECT: As can happen as a consequence of treatment induced increases in seizure threshold, the patient will be re-stimulated with a higher dosage in the same anesthetic session. This is the standard procedure for handling aborted seizures with ECT. If, however, the patient is already receiving stimulation at the highest output of the device when they have an aborted seizure (as would be the case in the high dose prefrontal ECT condition), the patient will be re-stimulated with the maximal output of the device during the same anesthetic session. If this also fails, then the patient would be dropped from the active phase of the study and offered routine clinical care with a proven effective treatment. That decision would be made by the patient in consultation with the treatment team.

III.I Clinical Ratings

Ratings will be conducted at baseline, within 24 to 48 hours of each MST/ECT session, 24-72 hours after the last MST/ECT session, and at 2 and 6 month follow up. Baseline and post-MST/ECT ratings will be videotaped, only if the subject consents to videotaping, for inter-rater and inter-site reliability. Although videotaping is not required for participation in this study, it helps to ensure that interviews are accurate and consistent. For remitters, ratings will continue bimonthly for two months, then monthly during Phase 2 through the 6-month follow-up. The primary clinical outcome measure is the $HRSD_{24}$ since this is the primary outcome measure in the ECT literature. The Inventory of Depressive Symptomatology – Clinician-Rated ($IDS\text{-}C_{30}$) will serve as a secondary outcome measure. Other clinical ratings will include the IDS-self report ($IDS\text{-}SR_{30}$), the Clinical and Patient Global Impression - Improvement (CGI-I, PGI-I), the Global Assessment of Functioning (GAF), and the Medical Outcomes Study Short-Form Health Survey (SF-36). The $IDS\text{-}C_{30}$ and the $IDS\text{-}SR_{30}$ provide objective and subjective ratings of antidepressant response using comparable items. The Columbia University ECT Side Effects Scale will be administered before and after the first and last treatment. This scale was sensitive in detecting differences between MST and ECT in acute somatic side effects. Subjective memory complaints will be assessed with the Cognitive Failures Questionnaire. The YMRS will be utilized to screen for treatment emergent hypomania or mania, which can be observed with ECT, but which has not been reported with MST. If treatment needs to be terminated due to hypomania or mania, the last $HRSD_{24}$ assessment prior to the emergence of mania will be used as the final $HRSD_{24}$ observation, and the patient will be classified as a nonresponder and nonremitter. This is expected to be a rare event.

Consistent with the standard of practice in ECT, there is no predetermined minimum or maximum number of treatments. The aim is to continue treatment until the patient achieves maximal improvement. If there is not significant clinical improvement by the 8th session ($\geq$25% drop in $HRSD_{24}$ from baseline at any point within the first 8 treatments), treatment will be stopped. Patients will be dropped from the study will be given routine clinical care with a treatment of known efficacy as clinically indicated by the PI's treatment team. If at any time during the study a patient attempts suicide, there is a clinical emergency, the treatment team judges that the patient should be dropped from the study, or a patient requests to stop treatment, the patient will be dropped from the study and provided clinically

appropriate treatment options. Otherwise, treatment will be continued until there has been a plateau or the subject achieves remission – whichever comes first. A plateau is defined as a decrease of 3 points or fewer in $HRSD_{24}$ score between a given treatment and the two successive treatments; this determination is only made starting with treatment 8. If the patient receives 15 treatments, the Director of the ECT Service will review the case with the clinical staff and document the clinical decision about continuing treatment. If more than 25 treatments need to be given, the Clinical Director is consulted and consent will be re-obtained.

Clinical response is defined as ≥50% drop in $HRSD_{24}$. Remission is defined as ≥60% drop in $HRSD_{24}$ ≤8.

Patients will be followed naturalistically for 6 months to monitor persistence of clinical benefit and of side effects. Ratings will be twice a month for 2 months, and once a month thereafter. To be classified as a relapse during the follow-up phase, patients must have two consecutive scores of $HRSD_{24} \geq 16$ and a ≥10 point increase in $HRSD_{24}$ maintained across 2 visits at least a week apart, or experience emergence of psychosis or suicidal ideation, or require hospitalization. This definition should capture sufficient worsening that would necessitate a change in clinical management. To characterize the nature of the naturalistic treatment received during Phase 2, information on treatment type, dosage, and duration of exposure will be collected at each assessment. We will follow nonremitters who subsequently receive ECT with pre and post $HRSD_{24}$ scores to determine whether ECT following MST results in a significant rate of response.

Regardless of outcome status, patients will be notified of their treatment condition only upon request, and only after testing of all study patients have ended.

III.J Neuropsychological Testing

The neuropsychological testing was modeled after that used in IDE# G000185 and IDE# G020028, to enable comparisons across studies. A technician masked to the treatment condition will administer neuropsychological tests at various time-points to assess the acute, short-term, and long-term neurocognitive effects of the treatments (see Table 2). A short version of the battery will be administered before and after each treatment session, while a longer version will be administered at baseline, within 7 days post-Phase 1, and at 2-month and 6-month follow up. This battery was designed to be comprehensive and sample an extensive range of cognitive functions to have clinical relevance and scientific value given the limited information regarding the effects of high power MST. The short- and long-term batteries were designed to sample those aspects of cognition (a) most vulnerable to ECT (e.g., retrograde and anterograde amnesia), and (b) most reliant on prefrontal lobe function (e.g., executive function tasks) which can be affected by neurostimulation therapies and are related to improvements in mood. Psychomotor tasks are included as controls. The battery includes measures to contrast actual performance with the patient's subjective experience and includes alternate forms. The Treatment Effects Battery (TEB) is a brief task that will be administered at each MST/ECT session, unless clinical circumstances prevent its completion by the patient. This battery samples multiple cognitive domains: 1) attention, using verbal and nonverbal cancellation tasks, 2) retrograde amnesia, using memory for words and shapes presented immediately prior to the treatment, and 3) memory for temporal order, and 4) time to recover orientation following treatment. TEB measures of domains one and two will be administered at baseline. Alternate forms of each task will be used on successive days. We

have substantial published data on the reliability and psychometric properties of the TEB, as well as on the effects of differing types of ECT on this battery.

Table 3. Schedule of Research Procedures

Assessments	Baseline	Treatment #1	Every Tx	Post Tx #6	Last Tx	Post Phase 1	2-Month	6-Month
**Clinical Ratings**								
Structured Clinical Interview for Diagnosis (SCID) – 1 hr	x							
Antidepressant Treatment History Form (ATHF) – 30 min	x							
Hamilton Rating Scale for Depression ($HRSD_{24}$) – 30 min	x	x	x	x	x	x	x	x
Inventory of Depressive Symptomatology ($-SR_{30}$, $-C_{30}$) – 10 min	x			x		x	x	x
Clinical Global Improvement (CGI) – 5 min	x					x	x	x
Patient Global Improvement (PGI) – 5 min	x					x	x	x
Global Assessment of Functioning (GAF) – 5 min	x					x	x	x
Young Mania Rating Scale (YMRS) – 10 min	x					x	x	x
Columbia ECT Subjective Side Effects Scale - 5 min	x	x	x	x	x	x		
**Quality of Life Ratings**								
Medical Outcomes Study Health Survey (SF-36) - 15 min	x					x	x	x
**Neuropsychological Battery - Acute Effects**								
Treatment Effects Battery – 45 min	x	x	x	x	x			
**Neuropsychological Battery - Short and Long-Term Effects 1 ½ days**								
Global Cognitive Function								
Mini Mental Status Exam	x					x	x	x
Wechsler Test of Adult Reading	x							
Anterograde Learning and Memory								
Buschke Selective Reminding Test (6 trials with 1/2 hr delay)	x					x	x	x
Complex Figure Copying (with 30 min delay)	x					x	x	x
Rey Auditory Verbal Learning Test (with 20 min delay)	x					x	x	x
Retrograde Memory								
Autobiographical Memory Interview - Short Form	x					x	x	x
Goldberg Remote Memory Questionnaire	x					x	x	x
Psychomotor								
Simple Reaction Time	x					x	x	x
Choice Reaction Task	x					x	x	x
Grooved Pegboard	x					x	x	x
Trail Making Test A	x					x	x	x
Executive Function								
Trail Making Test B	x					x	x	x
DKEFS Sorting Test	x					x	x	x
Stroop Color Word Test	x					x	x	x
Digit Span (WAIS-III)	x					x	x	x
Controlled Oral Word Association Test	x					x	x	x
Category Fluency	x					x	x	x
N-Back Working Memory	x					x	x	x
Go-No Go	x					x	x	x
Subjective Cognitive Evaluation								
Cognitive Failures Questionnaire	x					x	x	x

**Neurophysiological and Biochemical Studies**						
MST/ECT Seizure Threshold Titration		x				
Motor Cortex Excitability	x					x
EEG	x	x	x	x	x	x
Prolactin/ cortisol/ GABA		x		x	x	

III.K Side Effect Assessment and Patient Attitudes

Patients' attitudes about the treatment will be assessed with the ECT Attitude Interview. The Columbia University ECT Side Effects Scale will be administered before and after each treatment to assess subjective side effects. Subjective memory complaints will be assessed with the Cognitive Failures Questionnaire and subjective improvement will be measured with the Patient Global Improvement (PGI-I).

III.L Neurophysiological Assessments

All neurophysiological assessments will be performed when possible, if not prevented by equipment limitations or clinical urgency. Patients may refuse or elect to discontinue the neurophysiological procedures due to possible side effects or fatigue.

Neurophysiological assessments will be administered to examine the effects of MST and ECT seizure induction on regional patterns of brain activation (via topographical electroencephalography) and motor cortex excitability. Topographical electroencephalography (EEG) will provide data on the impact of focality of seizure induction on patterns of seizure onset and spread, factors thought to be critical to the efficacy and side effects of convulsive therapy. EEG will be conducted at pre-treatment baseline and during the first week post-treatment. In addition, EEG will be conducted during the MST/ECT course at the second and penultimate treatments. These acute studies involve EEG measurement for a 10 minute period prior to treatment, throughout the treatment period, and for 10 minutes in the immediate postictal period. EEG electrodes will be slotted (or made from plastic or other magnet-safe electrodes) to reduce electrode heating as recommended by the literature.

With single and paired-pulse TMS applied to the motor cortex, a battery of TMS motor cortex excitability measures will be performed in the week prior to and the week following the course of MST/ECT. This battery consists of motor threshold, central motor conduction time, paired pulse curve, silent period, and input/output curve. This battery is identical to that used in our prior IDE# G000185 and IDE# G020028. These studies will be conducted in approximately 4-5 hours total on 2 occasions (baseline and post MST/ECT course), with identical procedures at each assessment.

Motor Threshold (MT): MT is defined as the minimum magnetic flux needed to elicit a threshold EMG response (50 μV in peak to peak amplitude) in a target muscle in 5 out of 10 trials using single pulse TMS administered to the contralateral primary motor cortex. MT for the first dorsal interosseus muscle (FDI) will be determined in the left and right hands with optimal sti of the corresponding M1 site. The procedures recommended by the International Federation of Clinical Neurophysiology will be followed. Threshold will be determined in 2% increments of intensity, in a descending fashion.

Silent Period: Silent period refers to the suppression of EMG activity in the voluntarily contracted target muscle following the induction of a MEP. Studies of segmental spinal excitability during the silent period have established the cortical origin of at least the

later part of the evoked EMG silence. This post-excitatory cortically-generated inhibition can sometimes be observed in the absence of preceding facilitation (silent period without preceding MEP) and can be shown to have a distinct cortical origin from the optimal site for activation of a given target muscle. The balance of glutamatergic, dopaminergic, and GABAergic activity seems to play an important role in the duration of the silent period to TMS. The patients will be asked to perform a slightly isometric abduction of the index finger (about 10% of maximum force) starting 5 seconds before single-pulse TMS and maintaining tonic contraction for at least 5 seconds after single-pulse TMS. Stimuli will be delivered not closer than once every 20 seconds to avoid fatigue. Stimulus intensity will be set at 130% of motor threshold at rest. The individual motor responses will be rectified and averaged. The length of the silent period will be determined from the onset of the MEP to the recurrence of at least 50% of EMG background activity.

Input-Output Curve: Single TMS pulses of progressively increasing intensity applied to the motor cortex can be used to generate an input-output curve. The resulting modulation of amplitude of MEPs to increasing intensity of TMS pulses provides a measure of excitatory feedback to cortico-spinal efferent output that appears to be glutamatergically mediated.

Paired-Pulse Curve: Intracortical excitability can be further studied using the paired-pulse TMS technique. A first, subthreshold conditioning stimulus is applied, followed at a variable interval, by a second, test (suprathreshold) stimulus. The effects obtained depend on the intensity of the conditioning stimulus, the interval between the stimuli, and the intensity of the test stimulus. The intensity of the conditioning and test stimuli influence the effects as different circuits are recruited by different intensities of stimulation. The interstimulus interval (ISI) influences the results as the time constant of each activated circuit may differ. At very short ISIs (< 1 ms), it is possible to study neural time constants of the stimulated elements; at ISIs of 1-4 ms, it is possible to investigate interactions between I-wave inputs to cortico-spinal neurons; and at ISIs of 1-20 ms, it is possible to investigate cortico-cortico inhibitory and facilitatory circuits. All these effects appear to be cortically mediated and intracortical inhibition and facilitation appear to be derived from activation of separate circuits. The effects of different illnesses and medications on the inhibitory and facilitatory phases of the paired-pulse curve suggest that GABAergic and dopaminergic mechanisms are involved. Medications that enhance GABAergic activity have been shown to markedly decrease the degree of cortico-cortico facilitation evoked by paired TMS stimuli at ISIs of approximately 8-12 ms. Conversely, dopamine deficiency is associated with reduced cortico-cortical inhibition at short ISIs (<5 ms) and dopaminergic drugs have been shown to enhance cortico-cortical inhibition. Furthermore, studies suggest that an early phase of facilitation in the paired-pulse curve at approximately 3 ms ISI might be related to glutamatergic, excitatory intracortical inhibition.

Paired TMS stimuli will be applied through the same coil used for motor threshold determination using two Magstim 200 stimulators linked through a Magstim Bistim Module. The conditioning stimulus will be set at an intensity 20% below motor threshold. The second stimulus, which is referred to as the test stimulus, will be moderately (10%) suprathreshold, and its intensity will be adjusted to consistently evoke MEPs of approximately 1 mV peak-to-peak amplitude. The test stimulus will be preceded by the conditioning stimulus using 1 of 6 different ISIs in the following steps: 1, 3, 6, 8, 10, 12 ms. At each ISI, 10 MEPs will be recorded, and 60 additional MEPs will be recorded to the test stimulus alone. Therefore, a total of 120 MEPs will be recorded in each experiment, split in 3 blocks of 40 trials with a 5 minute rest period between blocks. Each block will consist of 20 single stimulus trials and 20 paired-pulse trials with two

different ISIs, presented randomly. The amplitude and area of the individual MEPs to the conditioned test stimuli will be measured on all single trials and the average values for the different ISI conditions will be calculated and expressed as percentages of the response to the test stimulus alone.

III.M Biochemical Studies

All biochemical studies will be performed when possible, if not prevented by equipment limitations, clinical limitations, or patient refusal.

For the prolactin, cortisol, and GABA assays, blood will be drawn immediately before and at 3 time-points following the seizure (5, 15, and 30 minutes) at the first MST/ECT session and the last MST/ECT session.

III.N Study Monitor and Monitoring Procedures

The primary study monitor is Dr. Lisanby. She will be assisted in her duties at the UTSW site by Dr. Husain.

Monitoring Procedures:

The purpose of the monitoring procedures is to assure the quality of the study and to aid the monitor in carrying out her duties. The monitoring procedure consists of three components: 1) pre-investigation visits, 2) periodic visits, and 3) review of patient records.

1) Pre–Investigation Visits

Prior to launching the trial, the monitor will have personal contact with each investigator at each of the two sites to establish that he/she:

- i) understands the investigational status of the device and the requirements for its accountability
- ii) understands the nature of the protocol or investigational plan
- iii) understands the requirements for an adequate and well–controlled study
- iv) understands and accepts the obligation to conduct the clinical investigation in accordance with Parts 812, 813, or any other applicable regulation
- v) understands and accepts the obligation to obtain informed consent in accordance with Part 56. (The monitor will review the consent forms at each site to assure that reasonably foreseeable risks are adequately explained)
- vi) understands and accepts the obligation to obtain IRB review and approval of a clinical investigation before the investigation may be initiated and to ensure a continuing review of the study by the IRB in accordance with Part 56 and to keep the sponsor informed of such IRB approval and subsequent IRB actions concerning the study
- vii) has access to an adequate number of suitable subjects to conduct the investigation
- viii) has adequate facilities for conducting the clinical investigation
- ix) has sufficient time from other obligations to carry out the responsibilities to which the investigator is committed by applicable regulations.

These pre-investigation steps are greatly facilitated for the New York State Psychiatric Institute (NYSPI) site because the monitor is also the principal investigator at that site and has already had extensive personal contact with the NSYPI investigators to ensure that they understand the points listed above. Dr. Husain, the lead investigator of the UTSW

site and co-principal investigator on the study, has had personal contact with the investigators at the UTSW site to ensure that they understand the above listed points.

2) Periodic Visits

The sponsor will assure throughout the clinical investigation that the investigator's obligations, as set forth in applicable regulations, are being fulfilled and that the facilities used in the clinical investigation continue to be acceptable. The sponsor will assure that:

i) the facilities continue to be acceptable for purposes of the study
ii) the study protocol or investigational plan is being followed
iii) changes to the protocol have been approved by the IRB and/or reported to the sponsor and the IRB
iv) accurate, complete, and current records are being maintained
v) accurate, complete, and timely reports are being made to the sponsor and IRB
vi) the investigator is carrying out the agreed–upon activities and has not delegated them to other previously unspecified staff.

These steps are greatly facilitated for the Duke University Medical center (DUMC) site because the monitor is also the principal investigator, thus the monitor already has intensive daily contact with all DUMC site investigators to ensure that they continue to comply with the points listed above throughout the investigation. Dr. Husain, the lead investigator of the UTSW site and co-principal investigator on the study, will maintain close personal contact with the investigators at the UTSW site to ensure that they continue to comply with the above listed points above throughout the investigation. Dr. Lisanby and Dr. Husain will maintain close personal contact throughout the conduct of the study through a combination of quarterly telephone conferences and an onsite visit at the midway point in the study.

3) Review of Site Records

Subject Records

The study monitor will review a sample of individual subject records at all sites to ensure that they are accurate and complete. Specifically, the monitor will check that the following items were completed, and the results of the review will be documented in the Monitoring Reports:

Evaluation Materials

- Evaluation materials were examined and approved by a study investigator before the first treatment, and approval was documented
    - If a washout was required, date of last medication was recorded and fell within the required time frame before treatment
- Informed consent has been documented in accordance with Parts 50 and 56
- Consent form signatures are present and clearly visible
- All evaluation materials are present
- Chart contains documentation that the subject met inclusion/exclusion criteria
- Drug test records are present and complete
    - All lab results are signed and dated by an MD
    - On-site urine screens are documented
    - Birth date on lab report is correct
    - Any repeat drug tests are properly documented

        - Progress note has been filed explaining why the test was repeated
        - Lab results are filed for all tests performed
- EKG records display the correct date administered
    - Any discrepancies have been corrected

Treatment Records
- There is documentation that checklists of all procedures to be done during a given treatment were looked over before and after the treatment

General Documentation
- All measures outlined in the protocol were performed at the designated time points:
    - Physiological measures
    - Blood work
    - Neuropsychological measures
    - Clinical ratings
- If measures were not performed at the designated time points, there was documentation of the circumstances surrounding the omitted measures
- All fields are filled out and all necessary information is present and correct:
    - Serial number of stimulation devices and coils
    - Date
    - Subject ID
    - Identification of all personnel who completed a form or administered treatment
- All handwriting is legible

Protocol Deviations
- Any protocol deviations were identified, reconciled, and are fully documented
    - Inclusion/exclusion criteria were met
    - Medication washout time fell within the correct range
- A note to file was included in the chart for each protocol deviation
- All deviations were reported to the study sponsor, and the report was documented
- If there was a deviation from the approved subject enrollment criteria, it was reported to the FDA
- Any event that could be misconstrued as a deviation is properly documented

This chart review will be performed during a periodic visit to UTSW. Additionally, charts will be sent from UTSW to DUMC for review and data entry after each subject completes the protocol.

Device Control
The study monitor will review the device control records at both sites to ensure that these records are complete and accurate. Specifically, the monitor will check shipping records to ensure that:
- Serial numbers are written on all device control records
- Records are complete
    - Documentation makes it clear when each device was shipped to and from the site

Personnel Records

The study monitor will review the personnel records at all sites to ensure that these records are complete and accurate. Specifically, the monitor will check the personnel records to ensure that they contain, for any person who performed a study procedure or filled out a study form:

- A Curriculum Vitae
- A signed investigator agreement
- A signed financial disclosure statement

4) Record of On-site Visits

The monitor will maintain a record of the findings, conclusions, and action taken to correct deficiencies for each on-site visit of an investigator. Such a record may enable FDA to determine that a sponsor's obligations in monitoring the progress of a clinical investigation are being fulfilled. The record will include:

i) the date of the visit
ii) the name and signature of the individual who conducted the visit
iii) the name and address of the investigator visited
iv) a statement of the findings, conclusions, and any actions taken to correct any deficiencies noted during the visit.

III.O Statistical Analyses

This is the first study to compare the antidepressant effects of a course of MST with ECT, thus formal power analysis cannot be preformed. The present study seeks to provide the initial data about the safety and efficacy of a full course of MST as compared to ECT.

The primary specific aims of this study are:

1) *To compare the antidepressant effects of MST and ECT.* This aim will be achieved by performing repeated measures analysis of variance (ANOVA) on the change in clinical ratings with treatment condition as the between-subjects factors, and clinical measure as the within-subjects factor. The primary clinical measure will be change in HRSD score. Dichotomous measures of clinical response (defined as ≥50% drop in HRSD) and remission (defined as ≥60% drop in $HRSD_{24}$ ≤8.) will also be examined.
2) *To compare the cognitive side effects of MST and ECT.* We predict that low dose MST will have fewer cognitive side effects than high-dose MST and ECT due to the focality and limited depth of seizure spread. To test this prediction, repeated measures analysis of variance (ANOVA) will be conducted on change in cognitive scores with treatment condition as the between-subjects factors, and neuropsychological task as the within-subjects factor.

IV.A. DEVICE DESCRIPTION

Magstim Theta (Magnetic Seizure Therapy)
Repetitive Rate Magnetic Stimulator

The following device description was prepared by the Magstim Company Limited. Correspondence with the device manufacturer is included in Appendix D.

IV.A.i. Introduction

The Magstim Theta repetitive magnetic stimulator is a research device that has been developed specifically to evaluate the efficacy of Magnetic Seizure Therapy (MST). The device is based upon the architecture and sub-systems of the generic Magstim Rapid² products. The Magstim Rapid² products are commercially available from The Magstim Company, have been in production for well over 2 years, and have FDA 510(k) clearance in the USA, reference K051864. The Theta system is designed to deliver stimuli with the same intensity as the generic Magstim Rapid² systems set at 100% power, but unlike the generic Rapid² systems it is capable of delivering a train of stimuli at 100Hz for up to 10s.

IV.A.ii. The Magstim Theta

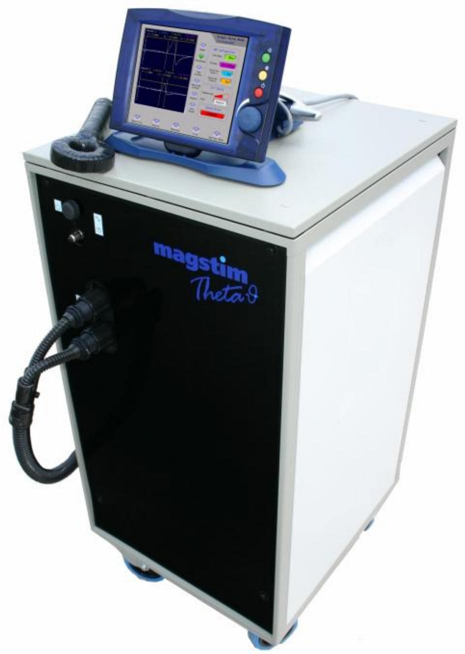

Fig. 6 Magstim Theta

- A 19-inch Cabinet – Contains Stimulator and Power Supplies
- 4 Castors – Diameter > 120 mm
- Coil connection located on the front face
- ON/Standby actuator located on the front face
- User Interface – Magstim 3022-00
- Power Supplies – Magstim 3512-01 (6 off)
- Upgraded wiring as compared with Rapid²
- Robust construction

IV. A.iii. A Summary of the Main Differences between Generic Magstim Rapid² and the Magstim Theta

Device	Standard Rapid²	Super Rapid²	Theta
Energy Storage	252J	252J	252J
No 3kW PSU Modules	1	2	6
Maximum Frequency at 252J	15Hz	25Hz	100Hz
Pulse Width (22µH coil)	400µs	400µs	400µs
Output Waveform	Biphasic	Biphasic	Biphasic
Power Requirements	3kVA	6kVA	18kVA

IV.A.iv. A Summary of the Main Differences between the Magstim Theta Coils and Previously Used Coils

COIL Parameter	'Theta' Coil	Standard 90mm Coil<br><br>Part # : 3193-00	Standard Double 70mm Coil<br>Part # : 3191-00	Safety Implications
Winding Inside Diameter	47mm	67mm	55mm	The differences in geometry affect the field distribution, but have no effect on the safe operation of the coil
Winding Outside Diameter	115mm	126mm	92mm	
# of Turns	18 - 2 layers stacked<br>9 turns each layer	14 turns	18 - 2 coils side by side, 9 turns per coil	
Cross-sectional Area of Winding Wire	10.5mm² (6mm x 1.75mm)	10.5mm² (6mm x 1.75mm)	10.5mm² (6mm x 1.75mm)	No difference
Primary Insulation (on winding wire)	3 layers "SAMICA" interspersed with<br>2 layers of NOMEX 50% overlap	Enamel	Enamel	The SAMICA/NOMEX insulation gives greater dielectric strength than Enamel, giving improved safety
Secondary Insulation	3 layers glass fabric tape, 50% overlap	1 layer glass fabric tape, 50% overlap	1 layer glass fabric tape, 50% overlap	3 layers of glass tape gives superior level of insulation.
Tertiary Insulation	1 layer glass fabric tape, 1	Encapsulation in 1mm thick	Encapsulation in 1mm thick	Glass tape is rated for 180°C and silicone is

	**layer high temp silicone self fusing tape, 1 layer glass fabric tape**	**high impact ABS**	**high impact ABS**	**rated for 260°C which is superior to ABS**
**Nominal Inductance**	**22µH**	**22µH**	**15µH**	**Inductance is the same as the 90mm coil**
**Nominal Resistance**	**<0.03□**	**<0.03□**	**<0.03□**	**No difference**

IV.A.v. Applicable documents

Magstim Rapid^2 Operating Manual P/N 3576-23
Magstim Theta Operating Manual P/N 3880-32

IV.A.vi. Functional Schematic Magstim Theta (MST)

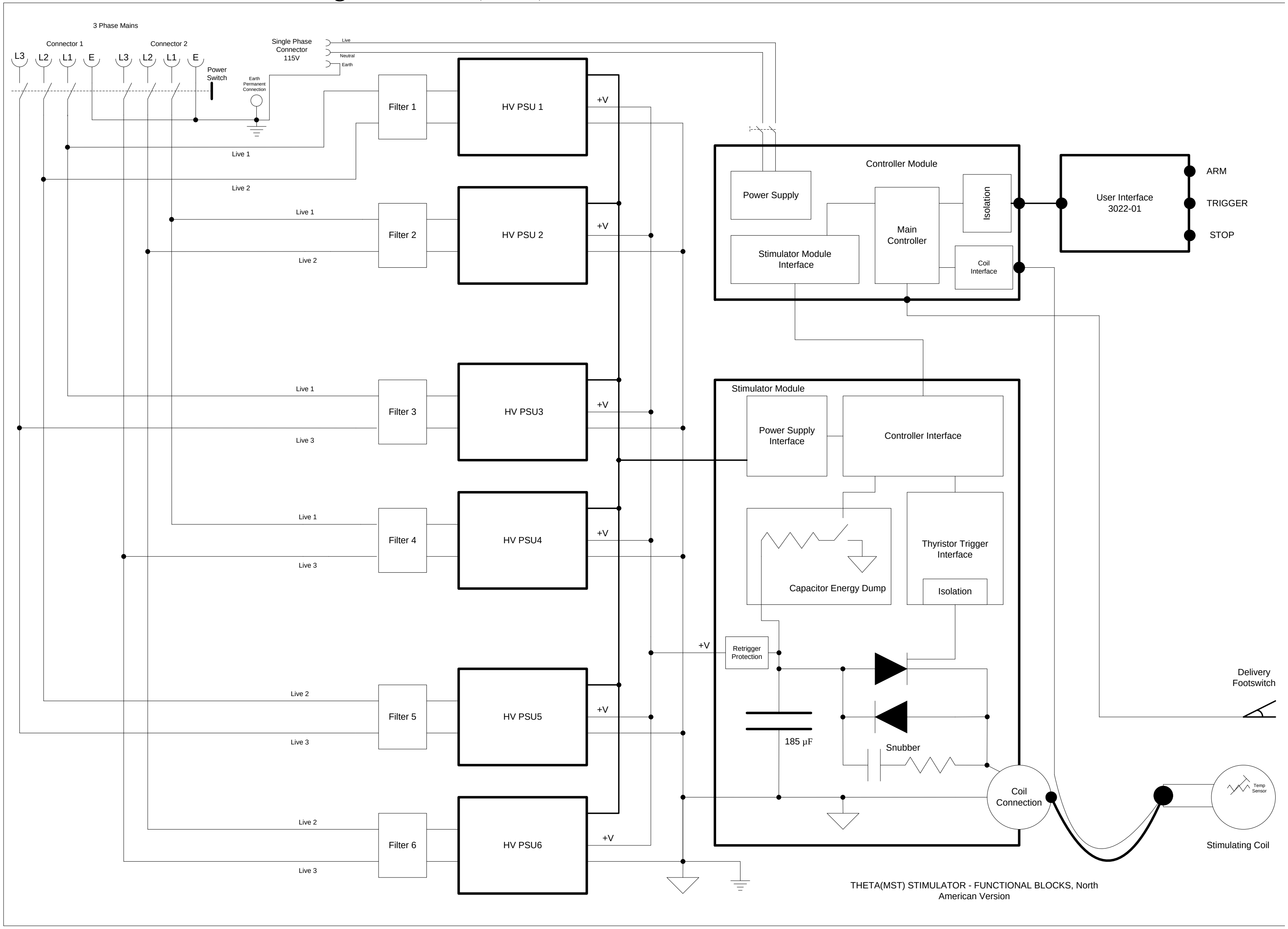

IDE Application - Magstim Theta Repetitive Magnetic Stimulator Page 28

IV.A.vii. COILS

Specialist coils have been developed for use with the Magstim Theta to deliver MST protocols. The Theta coils have a robust construction in order to handle the increased average energy levels associated with the Theta machine. Just as with the generic Rapid² system, each time the system fires approximately 125J of energy is lost. Most of this energy is dissipated as heat within the coil head. A loss of 125J, 100 times a second, equates to a power dissipation of 12.5kW in total and results in the coil head reaching temperatures in excess of 100°C. Safe use of the device has been considered and mitigated as follows:

- A substantial time lag exists between the energy being dissipated inside the coil and the temperature at the surface exceeding 41°C. Tests have shown that the coil temperatures do not exceed 41°C until approximately 50s after the end of the pulse train.

  The temperature at four locations on the surface of a 'Theta' coil (s/n: MP15) was monitored with LM35 temperature sensors, and logged with a Picolog datalogger. The system software will not allow a train to be initiated if the coil temperature is above 30°C, (to ensure that there is sufficient temperature margin to deliver a 10 second train). Consequently, the coil was warmed up by firing single shots until the internal temperature was 29.4°C (just under the 30°C interlock). The graph of figure 7 plots the coil surface temperature from the moment that a full power, ten second train of 100 stimuli per second was initiated, and shows that the surface temperature does not exceed 41°C until approximately 50 seconds after the pulse train ends.

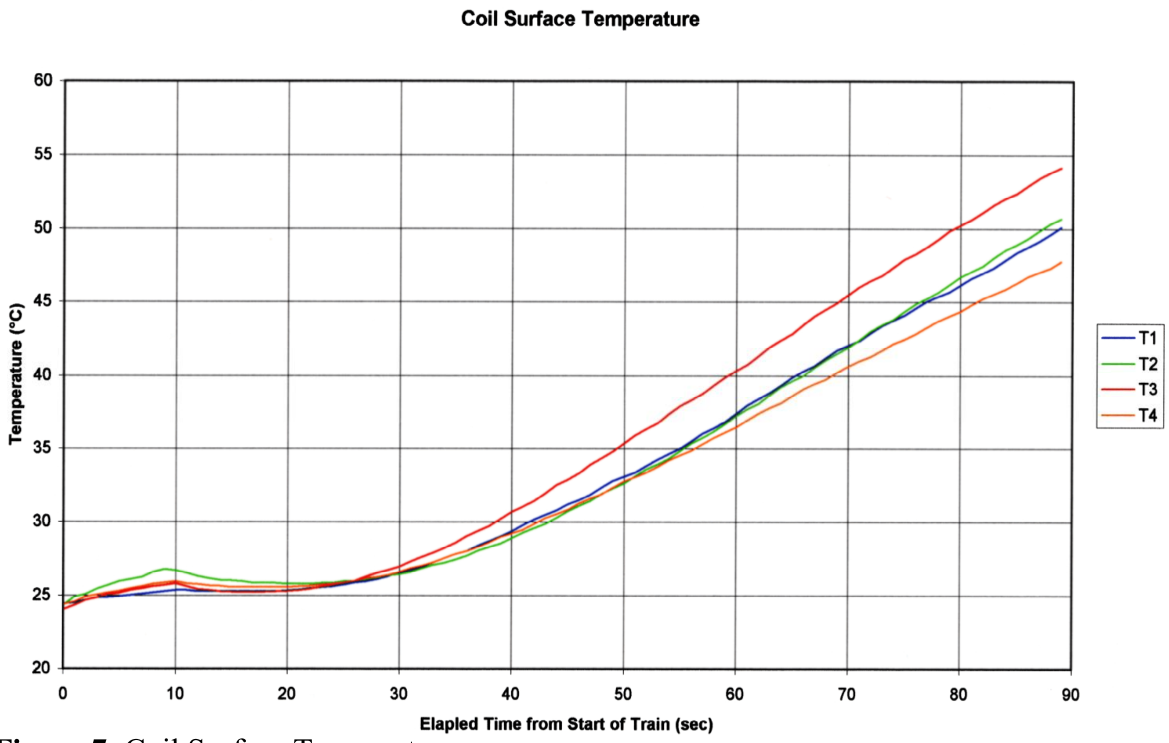

Figure 7: Coil Surface Temperature

The maximum safe temperature of the coil is 155°C . This is temperature rating of the "SAMICA" film that forms part of the insulation around each winding, and is the lowest of the temperature ratings of the various materials that are used in the construction of the coil head. The materials that provide electrical insulation between the coil windings and the patient/operator will withstand higher temperatures (Glass tape:180°C, Silicone tape: 260°C). If the coil temperature exceeds this 155°C , the insulation between the windings will start to degrade. The most likely consequence is that two or more of the coil windings will become shorted to one another. However, the stimulator is able to detect this fault, and will immediately shut down.

- Because of the specialist nature of the equipment, it must be used by trained operatives who are aware of the requirements of immediately removing patient contact with the coil after use.

- The system will not allow initiation of a train if the coil has a starting temperature of above 30°C. This ensures that there is a sufficient temperature margin to allow safe delivery of a 10s train. It also provides 50s leeway for removal of the coil before 41°C is reached.

- The coil is equipped with a temperature sensor, which provides a visual indication of the coil temperature via a numeric display on the system's front panel.

  The coil has a 'PT100' platinum resistance temperature sensor embedded between its windings. The accuracy of the temperature monitoring circuits inside the stimulator are checked as part of the stimulator test procedure. This is done with a test fixture that contains precision (0.1%) resistors, to simulate the temperature at two points (22°C & 44°C). In order to pass, the displayed and reported temperatures must lie within 1°C of the target value. (See appended system test procedure, sections C10 - C40).

  In addition, the coil test procedure includes a check to see that the temperature sensor. gives a value that is close to the ambient temperature, when the coil is nominally at room temperature. (See appended coil test procedure, section C40). The limits on this test are intentionally loose (+/- 6°C), because it is not possible to measure the internal temperature of the coil by any independent means. However, the most likely faults are a short or an open circuit, which this test would pick up. It is highly unlikely that the resistance of the actual sensor will have been changed by just a small amount.

- A temperature-sensitive label is attached to the coil head to provide an additional visual indication that the coil is hot.

- A test sample of the coil has been subjected to 220,000 shots (100% power, 100Hz, 10s train, 220 trains). Following completion of the test, the coil was dissected to determine whether there was any internal degradation. None was found. A second test coil is currently undergoing the same safety test and has so far been subjected to 3,000 shots.

- A shot counter has been included in the design of the Theta coil. This is programmed to prevent the stimulator from firing after the coil has been subjected to 110,000 shots at power levels in excess of 80%.

  The coil limit of 110,000 shots is a compromise between an acceptable lifetime and the time taken to perform verification testing. In order to be confident that the coil could withstand a specified number of shots, we concluded that we would have to test several samples for twice the that number.

  It was felt that a lifetime of 100 trains of 10 seconds duration, at full power, 100 stimuli per second (ie: 100,000 shots) was a reasonable expectation. However, it was also decided that before any coil could leave the company, it must be tested for 10,000 shots, in order to be sure that there are no manufacturing defects. So our goal of 100,000 shots for the end user, plus the requirement to test each coil for 10,000 shots is how we arrived at 110,000 shots.

  On the balancing side, this decision meant that the requirement for each coil undergoing verification testing would be 220,000 shots. Typically, it is only possible to subject a coil to 10 trains (10,000 shots) in a day, as time is required for the coil to cool after each train. Thus, each test takes 22 days.

- All coils are tested before release from The Magstim Company. The testing includes electrical safety and safe operation for 10,000 shots (100% power, 100Hz, 10s train, 10 trains).

  A copy of the coil test procedure (which also acts as the control sheet) is appended. The description that follows is a summary of the tests that are performed.

  Prior to the operational test, the coil undergoes a visual inspection. The first set of tests (C10 - C130) check the interlocks that prevent the system form operating if one (or more) of the coil connectors is disconnected. They also check the functionality of the temperature sensor, and the shot counter. All of these features are there to ensure the safety of the system.

  The next test (E105) simulates a shorted turn, and checks that the system is able to detect it. As discussed in the response to question 3, this is a potential failure mode, so the ability to detect it enhances patient safety.

  In tests E80 - E110, the coil is subjected to single discharges, as the power is increased from 50% to 100%, and the peak magnetic field generated by the coil is recorded. This ensures that nothing is amiss, and that the coil is generating the expected field levels, prior to subjecting the coil to a 10 second pulse train.

  The inductance and resistance of the coil is measured, in tests D10 and D20. If these three parameters lie within the expected limits, it gives a high degree of confidence that the coil will perform as intended.

Next (D30) the coil is subjected to a ten second, full power, 100 pulse per second train (1,000 shots). On completion of this test, the coil temperature is monitored. If it exceeds 150°C, the coil is rejected (D35). This ensures that the coil will not exceed its maximum safe operating temperature (155°C). Subsequently, after allowing the coil to cool, it is subjected to a further 8 trains (D40), to ensure that there is no internal manufacturing defect (such as a weak joint) that could cause it to fail.

On completion of 9000 shots at full power, the inductance (D50) and resistance (D60) are re-measured. If either has changed significantly, it indicates that something has changed inside the coil, and it will be rejected.

Tests B10 and B20 are performed to ensure that the coil is still electrically safe after enduring 9000 shots. A dielectric strength test is performed between the high voltage conductors and the external surface of the coil, using a test voltage of 11,000 volts (in excess of the requirement for double insulation, called out in EN60601, Clause 20.3). A second dielectric strength test is performed between the high voltage conductors and the low voltage signal conductors, using a test voltage of 3,000 volts.

Finally, the coil is subjected to one more train of 1000 pulses at full power, to ensure that it is still fully operational, after undergoing the dielectric strength tests.

IV.A.viii. Energy Storage

The discharge capacitor included within the Magstim Theta is 185µF and has the same value as the generic Magstim Rapid^2 products. The maximum voltage applied to this capacitance (Rapid^2 products and Theta product) is 1.6kV at a power setting of 100%; this corresponds to an energy store of 252J. This capacitance is implemented within the Magstim Theta via four of the capacitor types used in Rapid^2, configured in a series parallel arrangement. The increased number of capacitors ensures system reliability at 100Hz when operating at high power levels.

IV.A.ix. Dimensions

The photograph below gives a guide to the dimensions of the coil.

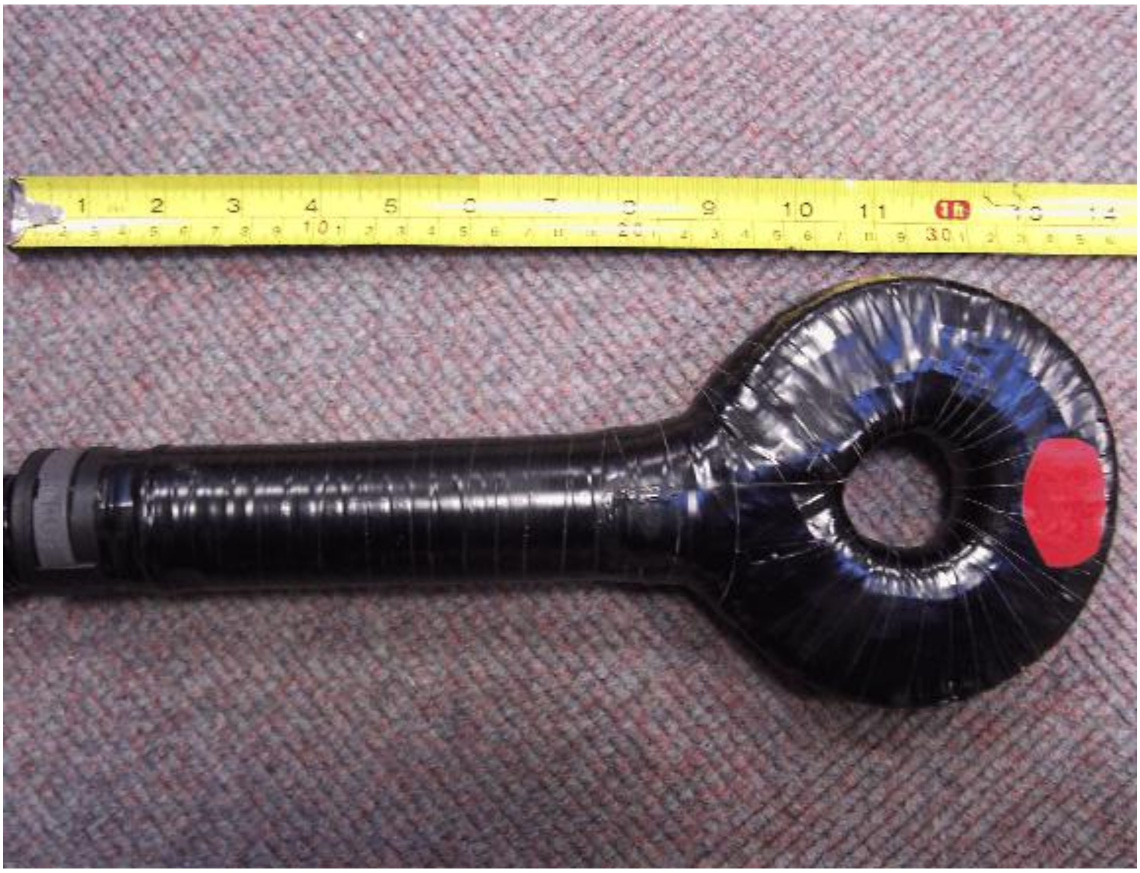

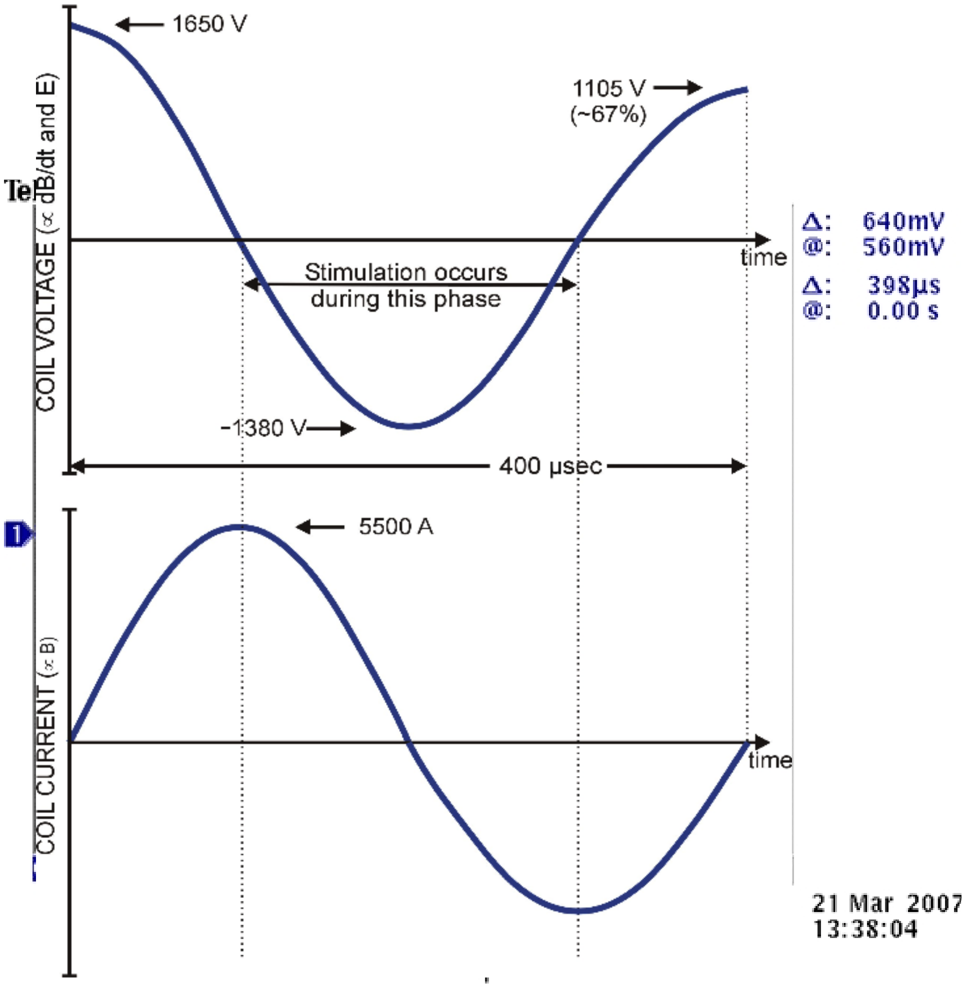

IV.A.x. Output Specifications and Magnetic Field

An oscilloscope screen capture of the 'Theta' stimulator's output waveform, as measured with a search coil, is shown in Figure 8, below:

This waveform was obtained with the stimulator set to maximum power. The search coil was positioned directly on the face of the theta coil, close to the innermost winding. This is the position at which the highest readings are obtained. The amplitude of the waveform is linearly proportional to the stimulator power setting, so at 50%, the amplitude is halved, but the shape remains unchanged.

A graphical representation of the Theta output waveforms is shown in figure 9, above. The upper waveform shows the voltage across the coil. This is proportional to the rate of change of magnetic field (dB/dt), the induced electric field (E) and hence the induced current. The lower waveform shows the current flowing in the coil, which is proportional to the magnetic field strength (B). The charge that accumulates across the nerve membrane is greatest during the middle phase of the waveform (when the coil voltage is negative), because the area under the curve is greatest. This is when stimulation occurs1

The output waveform of the 'Theta' stimulator is identical to that produced by the Magstim Super Rapid stimulator (510(k) file #: K992911) and Magstim Super Rapid2 stimulator (510(k) file #: K051864). This is because the amplitude of the waveform is dictated by the voltage to which the machine's internal pulse capacitor is charged, whilst the shape of the waveform is dictated by the capacitance of the pulse capacitor, and the inductance of the coil. All three systems utilize a maximum voltage of 1.65 kV, a pulse

discharge capacitance of 185µF, and are specified for use with coils whose inductance is in the range 15 - 22µH. Consequently, for any given coil, the output from the three systems will be indistinguishable.

	Magstim Super Rapic (Part #: 1450-00)			Magstim Super Rapic (Part #: 3005-00)			Magstim Theta		
	Pulse Capaci		Max Rep ra (sec^{-1}	Pulse Capaci		Max Rep rate (sec^{-}	Pulse Capacit		Max Rep rate (sec^{-}
Power Le	Volta (volt	Energ (joule		Voltage (volts)	Ene (jou		Volta (volts	Energ (joule	
100%	1,65	252	25	1,650	2	25	1,65	252	100
90%	1,48	204	25	1,485	2	30	1,48	204	100
80%	1,32	161	30	1,320	1	35	1,32	161	100
70%	1,15	123	30	1,155	1	40	1,15	123	100
60%	99	91	40	990	9	45	990	91	100
50%	82	63	40	825	6	50	825	63	100
40%	66	40	40	660	4	75	660	40	100
30%	49	23	50	495	2	100	495	23	100

The output specifications of the three systems are shown in the table, above. This clearly shows that the only difference between the systems is the stimulus repetition rate. The ‘Theta’ is capable of delivering 100 stimuli per second at all power settings. The Super Rapid2 system (3005-00) is only capable of delivering 100 stimuli per second at 30% power, falling to 25 stimuli per second at full power. The maximum repetition rate of the older Super Rapid (1450-00) is 50 stimuli per second, falling to 25 stimuli per second at full power.

The waveform shown in figure 10, below, represents both the region of maximum magnetic field strength (B) and the region of maximum rate of change of magnetic field strength (dB/dt), as they are the same place.

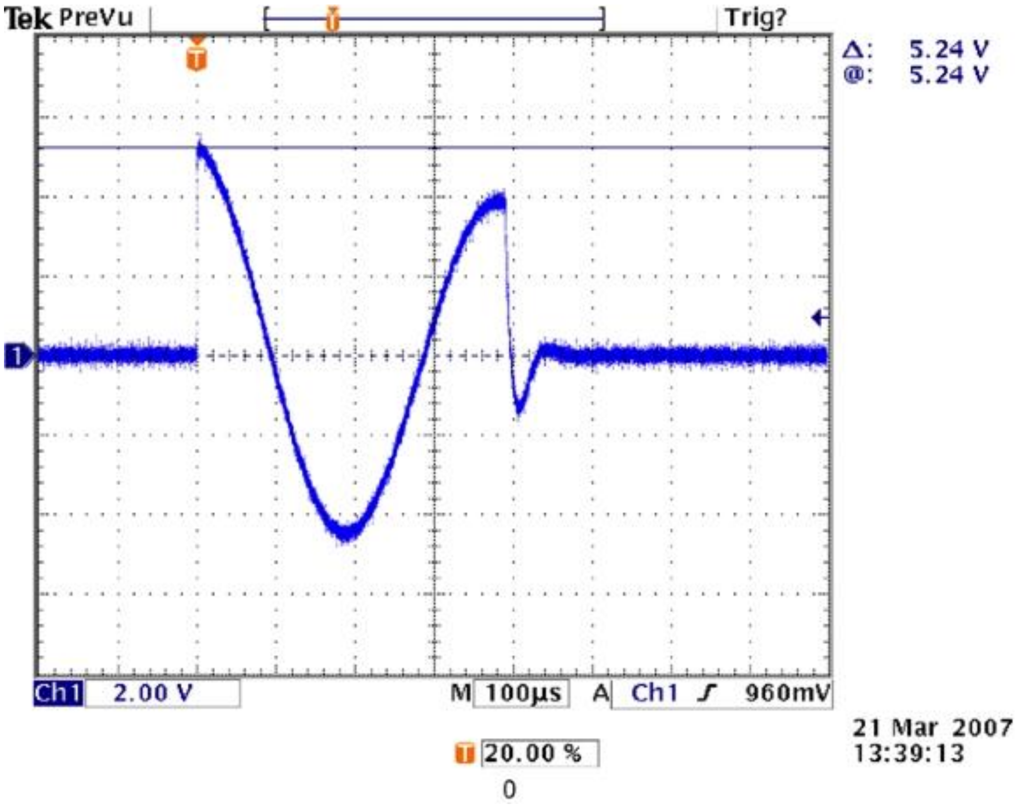

The waveform was obtained with a search coil positioned directly on the face of the theta coil, close to the innermost winding. This is the position at which the highest readings are obtained.

The voltage induced in a search coil is given by the simple formula:

$$V = N \times A \times \frac{dB}{dt}$$

where N is the number of turns, A is the area enclosed by the coil, and B is the component of the magnetic field that is perpendicular to the plane of the coil.

When a magnetic stimulator is discharged, the internal charge storage capacitor and the stimulation coil form a resonant circuit. The resonant frequency is given by:

$$f = \frac{1}{2 \times \pi \times \sqrt{C \times L}}$$

where C is the stimulator's internal capacitance and L is the inductance of

the coil. The capacitance is 185 µF and the inductance of the coil is 20 µH, which results in a resonant frequency of about 2.6 kHz.

Because of the sinusoidal nature of the discharge waveform, the equation for the voltage induced in the search coil can be rewritten as:

$$V_{(t)} = N \times A \times B_{pk} \times \omega \times \cos(\omega \times t)$$

Where $\omega = 2 \times \pi \times f$ and Bpk is the peak magnetic field.

The maximum rate of change occurs at the start of the discharge (ie: t = 0), so the equation becomes:

$$V_{(0)} = N \times A \times B_{pk} \times \omega$$

Rearranging gives:

$$B_{pk} = \frac{V_{(0)}}{N \times A \times \omega}$$

The search coil has 5 turns, and a diameter of 9mm. The peak voltage, measured at the start of the discharge is 5.24 volts. Using these values in the equation above gives a peak field or 1.008 tesla.

The plots that follow show the magnitude of the magnetic field that was measured from a theta coil, at distances from 0 to 10 cm from the face of the coil. The circular symmetry of the coil means that it is only necessary to measure the field along a single axis that bisects the coil, in order to characterize the field distribution in 3 dimensions.

The dashed line, in figure 11, represents the X-Y plane, along which measurements were taken. Theoretically, the magnetic field only has components in two directions; orthogonal to the plane of the coil winding (X), and parallel to the line that bisects the coil (Y). The field in the third (Z) direction should be zero. Figure 11, below, shows the orientation of the X, Y and Z directions relative to the coil.

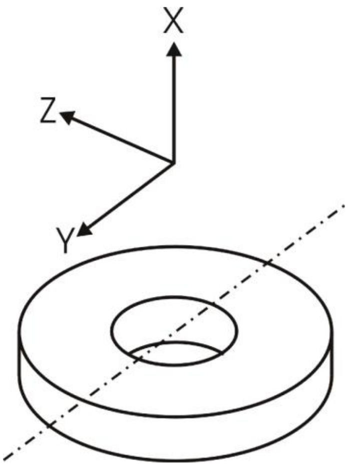

Figure 11: X,Y & Z axes

Measurements of the field in all three directions are presented in figures 12, 13 and 14. The vector sum is shown in figure 15.

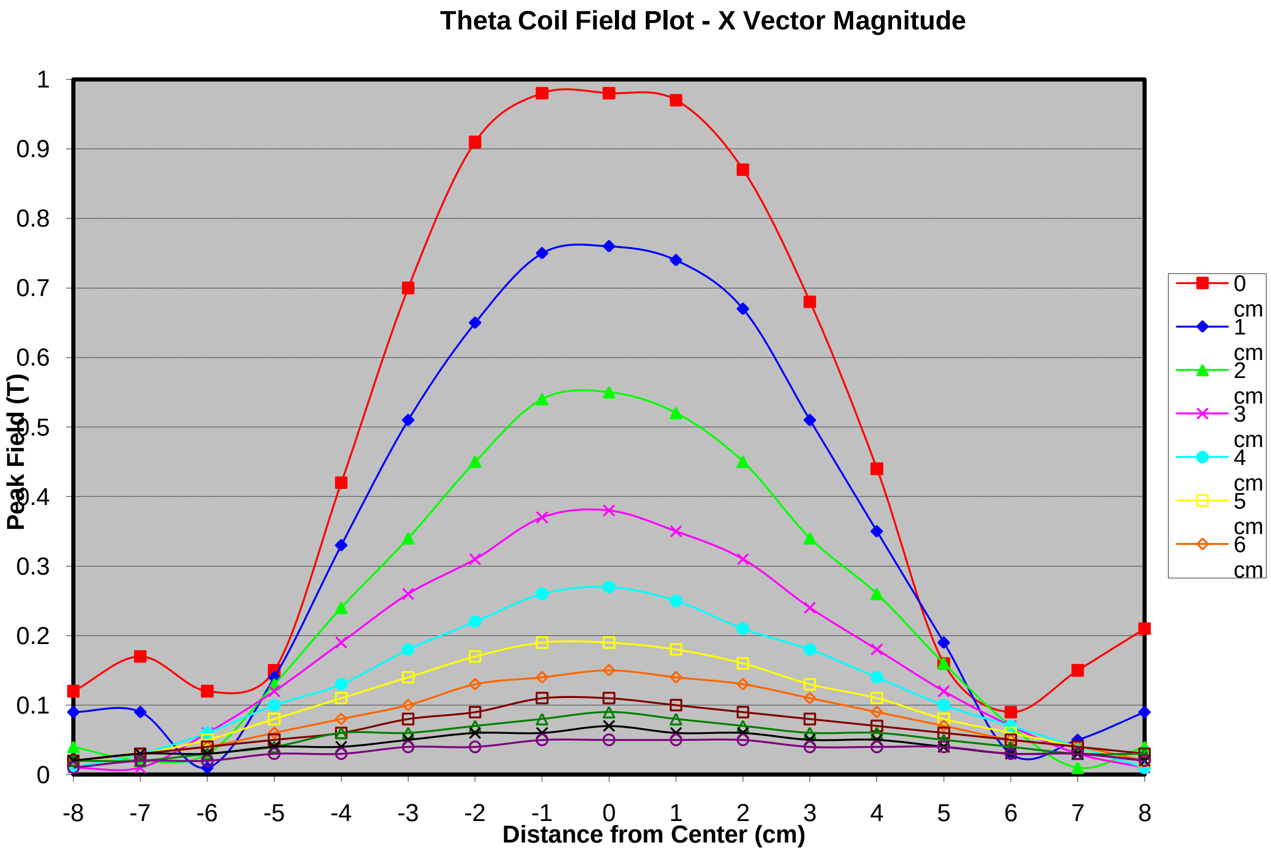

Figure 12: Field Strength in the 'X' direction

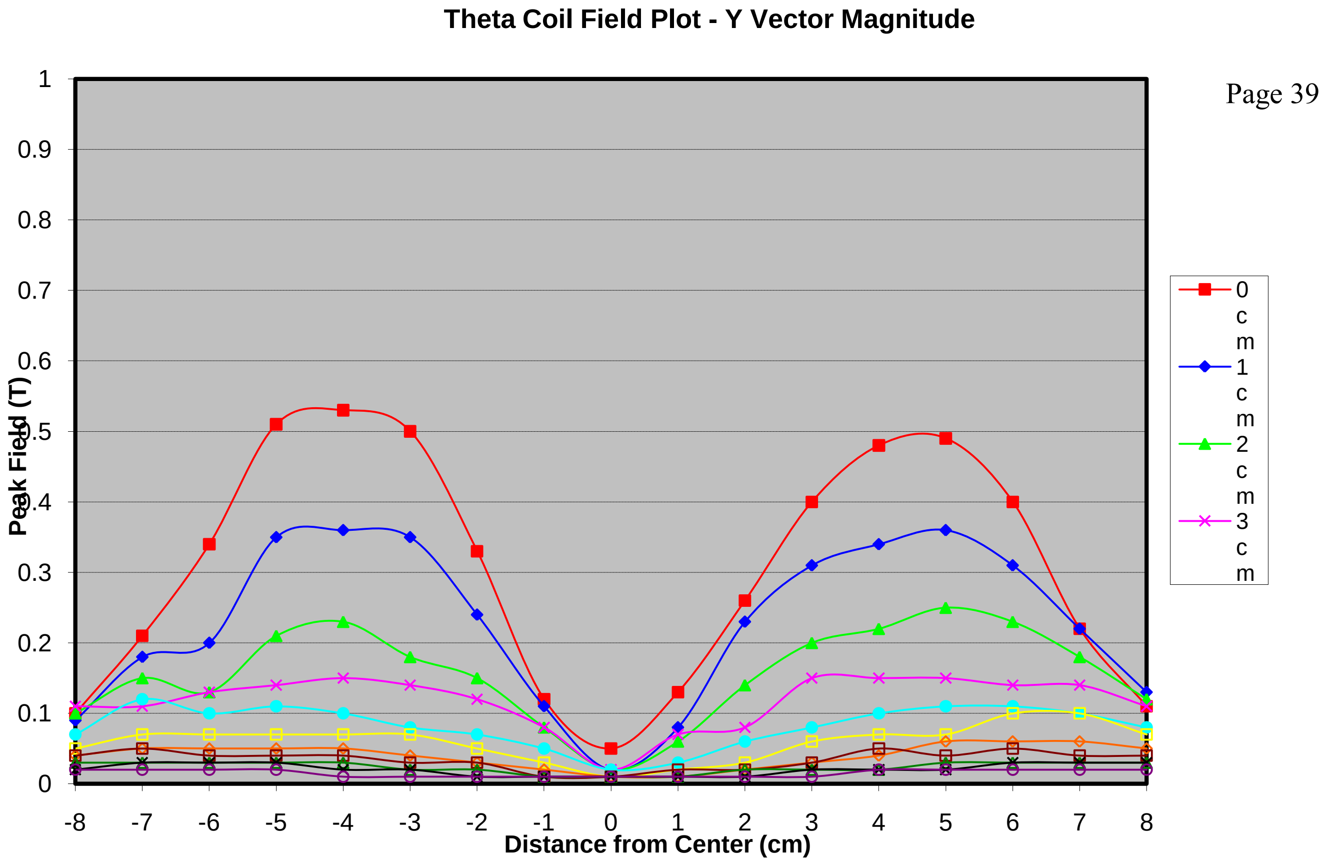

Figure 13: Field Strength in the ‘Y’ direction

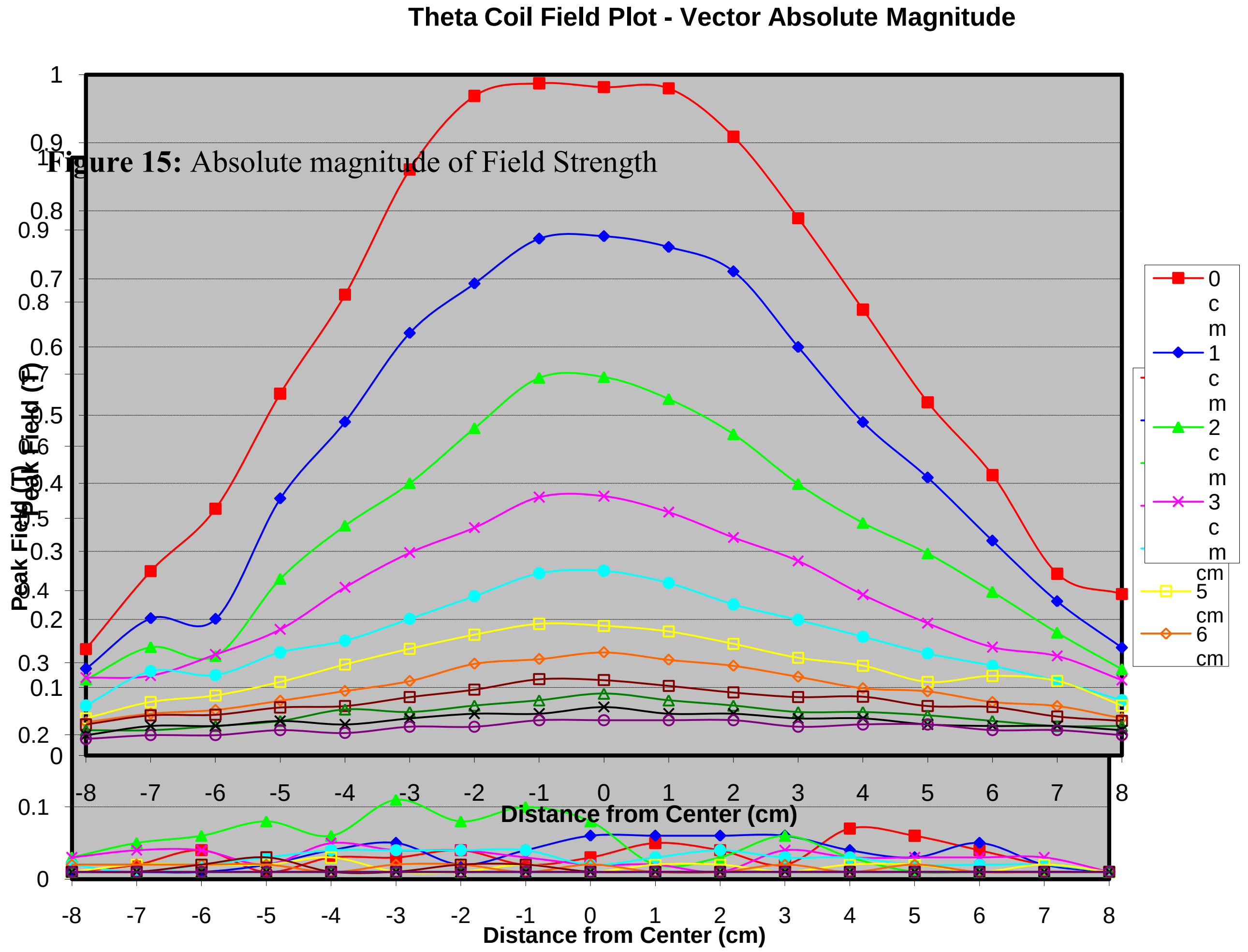

Figure 15: Absolute magnitude of Field Strength

Figure 14: Field Strength in the ‘Z’ direction

The oscilloscope traces were obtained with a 5 turn, 9mm diameter search coil and a Tektronics TDS3014 100MHz oscilloscope (Magstim asset # 116). The plots of field strength were obtained with a peak field meter (Magstim asset # 55). This device uses a 6 turn, 11mm diameter search coil, and was designed by the company specifically to take field measurements from its stimulator coils. The accuracy of both the oscilloscope and field meter are maintained under the company's calibration procedure.

The search coil was positioned manually, using a ruled scale as guidance, in order to obtain the plots of field strength.

Treatment Target Area:
The coil will be placed at the vertex of the head (location Cz). A picture of the coil placement is below:

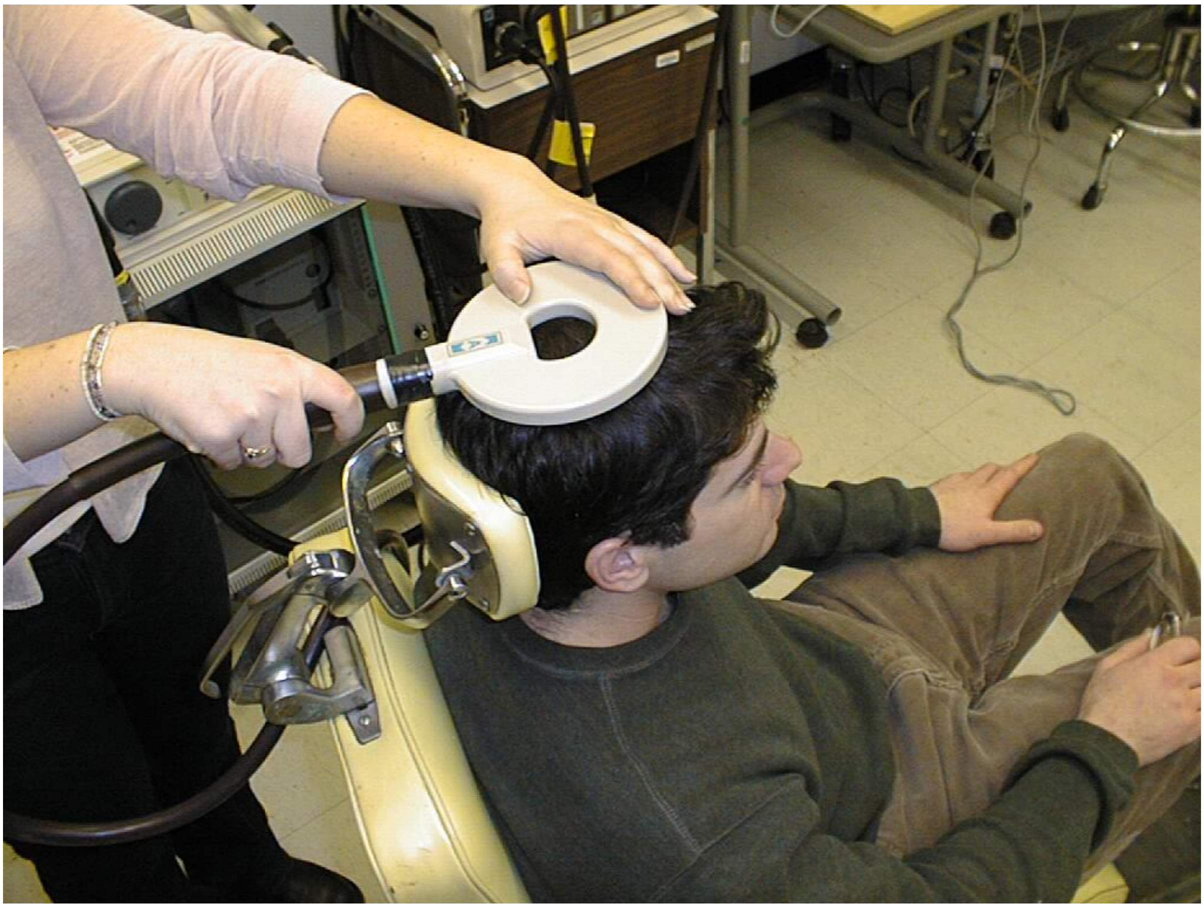

The target area will be the ring of lateral cortex tissue 2 cm deep from the coil windings, with a diameter equal to that of the coil. Acceptable ranges of therapeutic signal specifications for the signal parameters, including the magnetic field (B) and the time rate of change of the magnetic field (dB/dt) are described in the above plots, at the 2cm depth measurements.

IV.A.xi. Pulse Width

Theta coil inductance is typically 22.5μH. This value is comparable with the inductance of the 90mm circular coil specified for use with the Magstim Rapid² system. This inductance value, when combined with the discharge capacitor of 185μF and system losses, defines the discharge current waveform as a single-cycle damped sinusoid with a period of ~400μs.

IV.A.xii. Operating Frequency

The Magstim Theta is capable of delivering 100% power levels at 100Hz for 10s trains. The maximum performance of the Magstim Super Rapid² is 100% power at 25Hz for 10s trains.

IV.A.xiii. Power Requirements

- 3 Phase Mains (Powering PSUs)

  Type: 3 Live and Earth
  Voltage: 208Vac, 60Hz (Live to Live)
  Current: < 26A per phase
  Connector: 2 x Plug P+E 400V(Red) 16A, EN60309
  Cable Length: 5m (+1m, -0m)

- Single Phase Mains (Powering of Control)

  Voltage: 115Vac, 60Hz
  Current: < 2A
  Connector: Inlet IEC 320, C14

IV.B. DEVICE DESCRIPTION

IV.B.i. Introduction

This is a standard Thymatron ECT device that has clearance in the USA, FDA 510(k) reference number 945120.

IV.B.ii. Stimulus Output

Current: 0.9 amps constant current, limited to 450 volts, isolated from line current
Frequency: 10 to 70 Hz, in 10-Hz increments (to 140 Hz with LOW 0.25 Program)
Pulse width: 0.25 to 1.5 ms, in 0.25 ms increments
Duration: 0.14 to 7.99 s in increments of equal charge
Maximum output: Standard maximum output across 220 ohms in impedance, 504 mC (99.4 joules). Actual (delivered) treatment output shown on printed report in mC.
Stimulus waveform generation: bipolar, brief pulse, square wave

IV.B.iii. Recording

All four recording channels will be used:

- EEG 1 and EEG 2
  - 8 user-selectable gain positions: 10, 20, 50, 100, 200, 500, 1,000, and 2,000 μV/cm
- EMG
  - 8 user-selectable gain positions: 50, 100, 200, 500, 1,000, 2,000, 5,000, and 10,000 μV/cm
- ECG
  - 8 user-selectable gain positions: 50, 100, 200, 500, 1,000, 2,000, 5,000, and 10,000 μV/cm

IV.B.iv. Requirements

The device requires 100-130 volts (120 volts) AC, 60 Hz, single phase, 150 VA. It requires 220-240 volts, 50/60 Hz, switchable.

IV.B.v. Impedance

Static impedance test: 0 to 3,000 ohms static (+/- 100 ohms) at 800 Hz (LED and printed report)
Dynamic impedance measure: 0 to 500 ohms (printed report)

IV.B.vi. Seizure Monitoring

Channel specifications:

- Maximum gain: EEG, 10 μV/cm; EMG, 50 μV/cm; ECG, 50 μV/cm
- Common mode rejection: 80 dB
- Isolation: full, opto-electronic

- Printer paper speed: user selectable – 5 to 50 mm/s

Seizure quality measures:

- Postictal suppression index (EEG): range, 0-100%
- Average Seizure energy index (EEG)

IV.B.viii. Dimensions

- Weight: 22 lbs
- Height: 5.5”
- Width: 17.5”
- Depth: 13.0”

IV.B.ix. Applicable documents

THYMATRON SYSTEM IV OPERATING MANUAL

IV.C. DEVICE DESCRIPTION
MECTA SPECTRUM 5000Q ECT Instrument

IV.C.i. Introduction

This is a standard MECTA Spectrum 5000QECT device (U.S. Patent# 5,755,744 – U.S. Patent# 6,014,587) that has clearance in the USA, FDA 510(k) reference number K960754.

IV.C.ii. Stimulus Output

Current: 500 to 800 mA in 100 mA increments
Frequency: 20 to 120 Hz, in 10-Hz increments
Pulse width: 0.3 to 2.0 ms, in 0.10 ms increments
Duration: 0.5 to 8.0 s in increments of equal charge
Maximum output: Standard maximum output across 220 ohms in impedance, 558.3 mC (100.0 joules). Actual (delivered) treatment output shown on printed report in mC.
Stimulus waveform generation: constant current, bi-directional, square pulses, brief, ultra-brief

IV.C.iii. Recording

All four recording channels will be used:

- EEG 1
  - 8 user-selectable gain positions: .002, .004, .005, .010, .020, .025, .050, .100, mV/mm
- ECG
  - 8 user-selectable gain positions: .010, .020, .025, .050, .100, .200, .250, .500, mV/mm

IV.C.iv. Requirements

The device requires 115 volts nominally, 50/60Hz at .25 A Typical (idle) to 2.7 A max (treat), or 230 volts nominally, 50/60Hz at .13 A Typical (idle) to 1.4 A max (treat).

IV.C.v. Impedance

Static impedance test: 0 to 5,000 ohms nominally (LED and printed report)
Dynamic impedance measure: 0 to 300 ohms (printed report)

IV.C.vi. Seizure Monitoring

Channel specifications:

- EEG Channel gain: 5000 x from optional analog output (+/- 10%)
- EEG input range, AC: 2mV p-p max
- EEG input range, DC: +/- 200mV
- EEG frequency response: 1.4 to 48Hz band pass (-3dB)

- EEG common mode rejection: For 10V RMS, 50/60 Hz input having 200pF source capacitance, feeding unbalanced 51K/ .047 uF input network, resultant signal will be < 1 mV p-p R.T.I with notch filter off, and < .1 mV p-p R.T.I. with notch filter on
- Printer paper speed: 25 mm/sec (thermal printing method)

Seizure quality measures:

- Average Seizure energy index (EEG)

IV.C.viii. Dimensions

- Weight: 37 lbs
- Height: 6.9”
- Width: 20.4”
- Depth: 21.4”

IV.C.ix. Applicable documents

MECTA SPECTRUM 5000Q Operating Manual

V. ACTIVE INVESTIGATORS

Sarah H. Lisanby, M.D. Principal Investigator
Chair, Department of Psychiatry & Behavioral Sciences
Box 3950
Duke University Medical Center
Durham, NC 27710
Phone: 919-684-5616
Fax: 919-681-5489

Role on study and relevant experience: Study Principal Investigator and Study Monitor. Dr. Lisanby will operate the device, administer MST, be responsible for medical and psychiatric screening of patients, and supervise all aspects of the study. Dr. Lisanby has worked in the field of TMS since 1995. She received training in TMS through visits to the laboratory of Dr. Robert Post (National Institute of Mental Health, Bethesda, MD) in 1995 and Dr. Vahe Amassian in 1995-1996 (State University of New York, Health Sciences Center in Brooklyn, NY). She received training in the safety of TMS and in the various neurophysiological measures using TMS through visits to the laboratory of Drs. Mark Hallett and Eric Wassermann in 1995-96 (National Institute of Neurological Disorders and Stroke). She was the director for the Columbia University Department of Brain Stimulation and Therapeutic Modulation, which encompasses the ECT, MST, and TMS programs at Columbia University and the New York State Psychiatric Institute. She is now the Chair of Psychiatry and Behavioral Sciences at Duke University. She has served as principal investigator and study monitor for multiple IDEs employing TMS and MST. Dr. Lisanby originated the MST procedure, first in nonhuman primates and subsequently in human patients with depression. Dr. Lisanby is a licensed physician who is certified in emergency medical procedures (e.g., Advanced Cardiac Life Support) and is privileged to administer convulsive therapy at NYSPI.

Mustafa M. Husain, M.D. Principal Investigator, UTSW Site
Professor of Psychiatry and Internal Medicine
Director, Neurostimulation Laboratory
UT Southwestern Medical Center
5323 Harry Hines, Mail Code 8898
Dallas, Texas 75390
214-648-2806

Role on study and relevant experience: Dr. Husain will operate the device, administer MST, be responsible for recruiting, medical screening, following of participants, and will supervise all aspects of the study at the UTSW site. Dr. Husain has worked in the field of ECT since 1990. He received training in ECT at Duke University (1989-1991) and has been the Chief of ECT Services at UT Southwestern Medical Center since 1995 and has been the Director of the Neurostimulation Laboratory at UT Southwestern Medical Center since 2001. He has worked in the field of TMS since 2001. Dr. Husain received training in TMS and MST through visits to the laboratory of Dr. Sarah Lisanby (New York State Psychiatric Institute, NY) in 2000-2001 and 2005-2006. Dr. Husain is a Professor of Psychiatry and Neurology at UT Southwestern Medical Center and is the Chief of the Geriatric Psychiatry Division. He has served as principal investigator for multiple research studies and was the co-

Principal Investigator of the first MST study conducted with NYSPI. He has received training in TMS and MST procedures at NYSPI. Dr. Husain is a licensed physician and is privileged to administer convulsive therapy at UTSW.

Duke University/ DUMC Site Investigators:

Mohamed Aly, M.D. Co-Investigator
Research Associate, Senior,
Department of Psychiatry and Behavioral Science,
Duke University Medical Center,
Duke University
Durham, NC 27707
Phone: 919-613-6045

Role on study and relevant experience:
Dr. Aly is the study coordinator for this study at the DUMC site. He performs various treatment preparation procedures. His primary role will be data management, protocol compliance, and maintaining IRB documents. As an unmasked member of the research team, Dr. Aly will not complete any clinical ratings. Dr. Aly received his medical degree from El-Menia University, Egypt on 2000 and he finished his residency in psychiatry on 2005. He came to USA through a program funded by the Egyptian government to do his post doctoral research fellowship at Columbia University on 2008. Dr. Aly is appointed as associate research, senior in the department of psychiatry at Duke University starting 2010. Dr. Aly's clinical and research interests include electroconvulsive therapy (ECT), Magnetic seizure therapy (MST), transcranial magnetic stimulation (TMS) and deep brain stimulation. Dr. Aly is expert in delivering TMS in research and clinical setting and he is expert in doing different neurophysiological testing like Motor Battery and EEG.

Elisabeth Bernhardt Co-Investigator
Research Study Coordinator and Neuropsychological Rater
Department of Psychiatry and Behavioral Science,
Duke University Medical Center,
Duke University
Durham, NC 27707

Role on study and relevant experience:
Ms. Bernhardt will serve as a neuropsychological rater at the DU/DUMC site. As a masked member of the research team her primary function will be to serve as a backup neuropsychological rater, capable of performing neuropsychological ratings. Ms. Bernhardt has a BS from Mount Holyoke College, and has received considerable psychometric training involving neuropsychological testing. She has also served as a neuropsychological rater on various research studies.

Austin Harrison Co-Investigator
Research Study Coordinator and Neuropsychological Rater
Department of Psychiatry and Behavioral Science,
Duke University Medical Center,
Duke University
Durham, NC 27707

Role on study and relevant experience:
Mr. Harrison will serve as a clinical rater at the DU/DUMC site. As a masked member of the research team his primary function will be to serve as a clinical rater, capable of performing clinical ratings. Mr. Harrison has a BS in Psychology from The College of New Jersey, and has received considerable psychometric training involving clinical testing. He has also served as a clinical rater on various research studies.

Bruce Luber, Ph.D. Co-Investigator
Associate Professor of Psychiatry
Department of Psychiatry and Behavioral Science,
Duke University Medical Center,
Duke University
Durham, NC 27707

Role on study and relevant experience:
Dr. Luber will be responsible for the collection of neurophysiological data and will participate in data analysis and interpretation. Dr. Luber is an experimental psychologist who received his training at New York University in 1992. Dr. Luber has extensive experience in the design and conduct of neurophysiological studies. He received training in TMS from Dr. Lisanby in 1996 at the New York State Psychiatric Institute. Dr. Luber co-directed the Columbia University Transcranial Magnetic Stimulation Laboratory and has participated in multiple TMS and MST studies in humans and animals from 1995-present.

Moacyr Rosa, M.D. Co-Investigator
Research Associate, Senior,
Department of Psychiatry and Behavioral Science,
Duke University Medical Center,
Duke University
Durham, NC 27707

Role on study and relevant experience:
Dr. Rosa will perform various treatment preparation procedures for the MST study. Dr. Rosa completed his training in psychiatry at the University of Sao Paulo in Brazil in 2004. Dr. Rosa has extensive experience with brain stimulation techniques, including ECT, MST, VNS and Deep Brain stimulation (DBS). Dr. Rosa has an extensive experience with different animal experiments

Richard D. Weiner, MD, PhD Co- Investigator
Professor of Clinical Psychiatry.
Department of Psychiatry and Behavioral Science,
Duke University Medical Center,
Duke University
Durham, NC 27707
Office: 919 681 8742
Fax: 919 681 8744

Role on study and relevant experience:

Dr. Weiner will operate the device, administer MST as a backup to Dr. Lisanby, and participate in the medical and psychiatric screening and treatment of the patients. Dr Weiner received his medical degree and his PhD degree in physiology from Duke University on 1973 and then he finished his residency at UNC hospital on 1976. Dr. Weiner clinical interests are Affective disorders, electroconvulsive therapy, schizophrenia and electroencephalography. Dr. Weiner has a long history of research on electroconvulsive therapy. He is the director of ECT service at Duke University Medical center.

Julie Adams, MD, MPH Co- Investigator
Clinical Associate
Department of Psychiatry and Behavioral Science
Duke University Medical Center
Durham, NC 27710
Office: (919) 681-8347
Fax: (919) 684-8866

Role on study and relevant experience:
Dr. Adams will operate the device, administer MST as a backup to Dr. Lisanby, and participate in the medical and psychiatric screening and treatment of the patients. Dr. Julie Adams is the faculty mentor for psychiatry residents in the Duke Global Health Residency track. Before joining faculty she trained as a resident at Duke and traveled to Moshi, Tanzania for an extended elective rotation. She has since developed collaborations with researchers in Tanzania and across disciplines at Duke in the Department of Psychology, the Center for Health Policy, and the Duke Global Health Institute. These collaborations have lead to the rapid build-up of projects in Tanzania designed to study the intersection of mental illness and HIV infection, and to increase and improve mental health service provision in the region. As a senior research fellow in the Health Inequalities Program in the Center for Health Policy, Dr. Adams provides expertise to population-based studies conducted by a team of epidemiologists in multi-national, longitudinal studies. Her experience and collaborations provide a variety of opportunities for Global Health Residents to become involved and practice their clinical and research skills.

Mehul Mankad, MD Co- Investigator
Clinical Associate
2200 West Main Street, Suite 340
Durham, NC 27705
Office: 919 416 3439
Fax: 919 416 3437

Role on study and relevant experience:
Dr. Mankad will operate the device, administer MST as a backup to Dr. Lisanby, and participate in the medical and psychiatric screening and treatment of the patients. Dr. Mankad received his Medical degree from Northwestern University on1998 and finished his residency in psychiatry at Duke University Medical Center on 2002. He did his fellowship in Forensic Psychiatry at Rush-Presbyterian-St. Luke's Medical Center (Illinois) on 2003. His clinical interests are Medico-legal consultation, expert witness testimony, independent medical examination, general outpatient psychiatry and electroconvulsive therapy.

David C. Steffens, M.D., M.H.S. Co- Investigator
Professor of Psychiatry and Medicine
Head, Division of Geriatric Psychiatry
Duke University Medical Center, Box 3903
Durham, NC 27710
Phone (919) 684-3746
FAX (919) 681-7668

Role on study and relevant experience:

Dr. Steffens will operate the device, administer MST as a backup to Dr. Lisanby, and participate in the medical and psychiatric screening and treatment of the patients. Dr. Steffens completed his training in psychiatry at Duke University in 1992. He is certified in psychiatry on 1994 and recertified on 2005. He added another qualification on 1995 in geriatric psychiatry. Dr. Steffens has extensive experience with Electroconvulsive therapy. He has been the attending for ECT Service at Duke since 1996 till present. Dr. Steffens is a licensed physician who is privileged to administer convulsive therapy at CUMC.

Harold W. Goforth, MD Co- Investigator
Assistant Professor of Clinical Psychiatry
Box 3903 Med Center
Durham, NC 27710
919 681 8742

Role on study and relevant experience:

Dr. Goforth will operate the device, administer MST as a backup to Dr. Lisanby, and participate in the medical and psychiatric screening and treatment of the patients. He received his MD from Wright State University, Boonshoft School of Medicine (Ohio) on1998. He finished his residency in General Adult Psychiatry in Loyola University Medical Center (Illinois) on 2004. He finished his fellowship in Geriatric Psychiatry at Duke University Medical Center on 2005. Dr. Goforth's clinical interests are Consultation-liaison psychiatry, ECT, HIV-AIDS psychiatry, pain medicine and palliative medicine. His research interests are HIV/AIDS, Delirium Consultation-Liaison Psychiatry and Major Affective Disorders.

Scott Moore, M.D., Ph.D. Co- Investigator
Associate Professor of Clinical Psychiatry,
Box 3309 Medical Center
Durham, NC 27710
Office: 919 681 8742
Fax: 919 681 8744

Role on study and relevant experience

Dr. Moore will operate the device, administer MST as a backup to Dr. Lisanby, and participate in the medical and psychiatric screening and treatment of the patients. Dr. Moore got his MD, PhD degree from University of Virginia School of Medicine on 1986. He finished his residency in Duke University medical center on 1992. His clinical interest is Psychopharmacology, electroconvulsive therapy, neurobehavioral

disorders. His research interests include electroconvulsive therapy and translational research.

Personnel at Columbia site for follow up:

Stefan Rowny, M.D. Co-Investigator
College of Physicians and Surgeons of Columbia University
Postdoctoral Clinical Fellow, Department of Psychiatry
New York State Psychiatric Institute
1051 Riverside Drive, Box 21
New York, NY 10032
(212) 543-6055

Role on study and relevant experience:
Dr. Rowny will participate in the follow up of patients at the Columbia Follow up site. Dr. Rowny completed his training in psychiatry at UCLA in 2004. He received his training in convulsive therapy at Columbia University where he has been on the faculty since 2007. Dr. Rowny has extensive experience with brain stimulation techniques, including ECT, MST and rTMS. Dr. Rowny is a licensed physician who is privileged to administer convulsive therapy at NYSPI.

Nancy Turret, M.S.W. Co-Investigator
Instructor in Clinical Psychology, Department of Psychiatry
New York State Psychiatric Institute
1051 Riverside Drive, Box 21
New York, NY 10032
(212) 543-5657

Role on study and relevant experience:
Ms. Turret will serve as a clinical rater at the CU/NYSPI site. Ms. Turret performs clinical ratings and performs initial screening evaluations to determine participant eligibility for the MST study. She completed her training in social work at Fordham University in 1978. She has been involved in additional research protocols at the Division of Brain Stimulation and Therapeutic Modulation.

Anouk Allart Co-Investigator
Research Study Coordinator and Neuropsychological Rater
Division of Brain Stimulation and Therapeutic Modulation
New York State Psychiatric Institute
1051 Riverside Drive, Box 21
New York, NY 10032
(212) 543-5615

Role on study and relevant experience:
Ms. Allart will serve as a neuropsychological rater at the CU/NYSPI site. As a masked member of the research team her primary function will be to serve as a backup neuropsychological rater, capable of performing neuropsychological ratings.

UTSW Site Investigators:

Marius Commodore, M.D. Co-Investigator
Assistant Professor of Internal Medicine and Psychiatry
Department of Psychiatry
UT Southwestern Medical Center
5323 Harry Hines
Dallas, Texas 75390

Role on study and relevant experience:
Dr. Commodore will serve as a co-investigator and back up treatment provider at the UTSW site. Dr. Commodore will operate the device and administer MST treatment and assist in the recruitment of subjects. He is a licensed physician, and currently serving as an Assistant Professor of Internal Medicine and Psychiatry in the Department of Psychiatry at UT Southwestern Medical Center. He completed his undergraduate degree at the University of Michigan and medical school at Emory University school of Medicine.

Paul Croarkin, M.D. Co-Investigator
Assistant Professor, Department of Psychiatry
UT Southwestern Medical Center
5323 Harry Hines
Dallas, Texas 75390

Role on study and relevant experience:
Dr. Croarkin will serve as a co-investigator and back up treatment provider. Dr. Croarkin is a licensed physician, currently serving as an Assistant Professor in the Department of Psychiatry at UT Southwestern Medical Center/Children's Medical Center. Dr. Croarkin has been involved in multiple research protocols. Dr. Croarkin completed his undergraduate degree at Southwest Missouri State University and medical school at University of North Texas Health Science Center.

C. Munro Cullum, Ph.D. Co- Investigator
Professor of Psychiatry and Neurology
Director of Neuropsychology
UT Southwestern Medical Center
5323 Harry Hines, Mail Code 8898
Dallas, Texas 75390
214-648-3353

Role on study and relevant experience: Dr. Cullum will supervise all aspects of the collection of neuropsychological data at the UT Southwestern Medical Center site. He will participate in the statistical analyses of these data. Dr. Cullum is the Director of Neuropsychology and Professor of Psychiatry and Neurology at UT Southwestern Medical Center. He has received training in neuropsychiatry at Colorado State University (1989-1994), University of California at San Diego (1985-1986), and Fort Lyon VA Medical Center (1986-1988). He previously served as the executive director of the National Academy of Neuropsychology. He has been a co-investigator and neuropsychiatry consultant on multiple research studies and was the Chief Neuropsychologist of the UTSW site on the initial MST project. Dr. Cullum is a licensed psychologist.

Hayley Evans Co-Investigator
PhD Doctorial Student
Department of Psychiatry, Division of Psychology
UT Southwestern Medical Center
5323 Harry Hines
Dallas, Texas 75390

Role on study and relevant experience: Ms. Evans will serve as a backup study administrator for the MST study at the UTSW site. Her primary role will be to assist in any clerical or administrative tasks, which include data management and regulatory issues. Ms. Evans has a BA in Psychology from Southern Methodist University and has served as a research assistant and a clinical data specialist. Ms. Evans is currently enrolled in the PhD Clinical Psychology Graduate Program at UT Southwestern Medical Center.

Andrew Kozel, M.D. Co-Investigator
Assistant Professor, Department of Psychiatry
UT Southwestern Medical Center
5323 Harry Hines
Dallas, Texas 75390

Role on study and relevant experience:
Dr. Kozel will serve as a co-investigator and back up treatment provider. Dr. Kozel is a licensed physician, currently serving as an Assistant Professor in the Department of Psychiatry at UT Southwestern Medical Center. He has worked in various positions that have involved both neurostimulation and neuroimaging research. Dr. Kozel has also authored multiple peer review publications in the field of neurostimulation and neuroimaging. He completed his undergraduate degree at Yale University and medical school at University of Virginia School of Medicine.

Shawn M. McClintock, Ph.D. Co-Investigator
Assistant Professor of Psychiatry
Department of Psychiatry
5323 Harry Hines Blvd., Mail Code 8898
Dallas, TX 75235
(214) 648-2806

Role on study and relevant experience: Dr. McClintock will coordinate the MST study at the UTSW site and will assist with study implementation, the treatment-effects battery, and will be involved in the collection, integrity, and analysis of the neuropsychological battery. Dr. McClintock is a NIMH postdoctoral research fellow in the Department of Psychiatry at UT Southwestern Medical Center. He received his training in the Departments of Clinical Psychology and Psychiatry at UT Southwestern Medical Center (2002-2006). Dr. McClintock received training in TMS and MST through visits to the laboratory of Dr. Sarah Lisanby (New York State Psychiatric Institute, NY) in 2000-2002 and 2005-2006. He assisted with the treatment-effects battery, clinical interviews, and neuropsychological procedures, on the initial MST study at UTSW.

Najeeb Ranginwala, M.D. Co-Investigator

Research Study Coordinator
Department of Psychiatry
UT Southwestern Medical Center
5323 Harry Hines
Dallas, Texas 75390

Role on study and relevant experience:
Dr. Ranginwala will serve as a research assistant at the UTSW site. Dr. Ranginwala performs various treatment preparation procedures and performs initial screening evaluations to determine participant eligibility for the MST study. He completed his undergraduate education at Adamjee Science College in Pakistan and his medical training at Dow Medical College in Pakistan. Dr. Ranginwala has also completed additional medical training at Alton Ochner Medical Foundation in New in Louisiana, and at UT Southwestern Medical Center. He has been involved in additional research protocols involving neurostimulation research.

Ahmad Raza, M.D. Co-Investigator
Associate Professor of Psychiatry
UT Southwestern Medical Center
5323 Harry Hines, Mail Code 8898
Dallas, TX 75390
(214) 648-2806

Role on study and relevant experience: Dr. Raza will operate the device and administer MST as back-up as well as assist in the recruitment of subjects. Dr. Raza is the Medical Director of the K. Z. Altshuler Psychiatric Unit at Zale Lipshy University Hospital and is an Associate Professor of Psychiatry at UT Southwestern Medical Center. Dr. Raza has received training in psychiatry and training ECT. He has been involved in multiple research protocols. Dr. Raza is a licensed physician and is privileged to administer convulsive therapy.

Charlena Rodez
Clinical Data Specialist and Clinical Rater
Department of Psychiatry
UT Southwestern Medical Center
5323 Harry Hines
Dallas, Texas 75390

Role on study and relevant experience: Ms. Rodez will serve as the primary clinical rater for the MST study at the UTSW site. As a masked member of the research team her primary role will be to perform clinical and neuropsychological ratings. Ms. Rodez has a BA in Psychology from St. Mary's University, and has received considerable psychometric training involving both clinical and neuropsychological testing. She has also served as a clinical rater on additional depression and neurostimulation studies.

Judy Shaw
Research Study Coordinator
Department of Psychiatry, Division of Psychology
UT Southwestern Medical Center

5323 Harry Hines
Dallas, Texas 75390

Role on study and relevant experience:
Ms. Shaw will serve as a clinical rater for the MST study at the UTSW site. As a masked member of the research team her primary function will be to serve as a backup rater, capable of performing clinical and neuropsychological ratings. Ms. Shaw has a BA in Psychology from the University of Texas at Dallas. She has received considerable psychometric training involving both clinical and neuropsychological testing. Ms. Shaw has also serves as Chief Psychometrist of the Neuropsychology Division (Department of Psychiatry) at UTSW since 2003, and has been involved in training and supervision of neuropsychological testing. Ms. Shaw has been involved in multiple research protocols, and is a certified specialist in psychometry.

Louis Stool, M.D. Co-Investigator
Associate Professor of Anesthesiology and Pain Management
UT Southwestern Medical Center
5323 Harry Hines, Mail Code 9068
Dallas, TX 75390
(214) 648-7818

Role on study and relevant experience: Dr. Stool is a board certified anesthesiologist with extensive experience in administering anesthesia for ECT and MST. He will be responsible for administering the anesthesia for the MST and ECT sessions, and supervising the anesthesia protocols.

Kenneth Trevino Co-Investigator
PhD Doctorial Student
Department of Psychiatry, Division of Psychology
UT Southwestern Medical Center
5323 Harry Hines
Dallas, Texas 75390

Role on study and relevant experience: Mr. Trevino will serve as a study coordinator for the MST study at the UTSW site. His primary role will be data management, protocol compliance, and maintaining IRB documents. As an unmasked member of the research team Mr. Trevino will not complete any clinical ratings. Mr. Trevino has a BA in Psychology from the University of Texas at Arlington and has served as clinical data specialist for various clinical research studies. Currently he is enrolled in the PhD Clinical Psychology Graduate Program at UT Southwestern Medical Center.

VI. INVESTIGATOR AGREEMENT

A signed Investigator Agreement by each of the active investigators is included in Appendix C. These agreements have been signed by:

Caren Abitbol
Anouk Allart
Julie Adams, MD, MPH
Mohamed Aly, M.D.
Elisabeth Bernhardt
Peter Bulow, M.D.
Marius Commodore, M.D.
Paul Croarkin, M.D.
C. Munro Cullum, Ph.D.Hayley Evans
Harry Goforth, M.D.
Austin Harrison
Mustafa M. Husain, M.D.
Andrew Kozel, M.D.
Sarah H. Lisanby, M.D.
Bruce Luber, Ph.D.
Mehul Mankad,, MD
Shawn M. McClintock, Ph.D.
Regena Mitschke
Scott Moore, M.D., Ph.D.
Joan Prudic, M.D.
Najeeb Ranginwala, M.D.
Ahmad Raza, M.D.
Charlena Rodez
Moacyr Rosa, M.D.
Stefan Rowny, M.D.
Judy Shaw
Alexandra Sporn, M.D.
Arielle Stanford, M.D.
David Steffens, M.D.
Louis Stool, M.D.
Kenneth Trevino
Nancy Turret, M.S.W.
Richard Weiner, MD

VI.A Certification of Investigator Agreement

I certify that all personnel participating in the investigation have agreed to follow the applicable clinical investigational protocol contained in this application. I have submitted the written protocol to the Institutional Review Board. All investigators participating in this investigation understand that they are bound to adhere to it as a condition of IRB approval, when granted. I certify that the list of investigators in this application identifies all current investigators. Additional participating investigators in the investigation will be added to the investigation only when they have obtained IRB approval and have signed an investigator agreement.

____________________ 12/30/2010
Signature Date

Sarah H. Lisanby, MD
Name (print)

Investigators who have signed the agreement:

Anouk Allart
Julie Adams, MD, MPH
Mohamed Aly, M.D.
Elisabeth Bernhardt
Marius Commodore, M.D.
Paul Croarkin, M.D.
C. Munro Cullum, Ph.D.Hayley Evans
Harry Goforth, M.D.
Austin Harrison
Mustafa M. Husain, M.D.
Andrew Kozel, M.D.
Sarah H. Lisanby, M.D.
Bruce Luber, Ph.D.
Mehul Mankad,, MD
Shawn M. McClintock, Ph.D.
Scott Moore, M.D., Ph.D.
Najeeb Ranginwala, M.D.
Ahmad Raza, M.D.
Charlena Rodez
Moacyr Rosa, M.D.
Stefan Rowny, M.D.
Judy Shaw
David Steffens, M.D.
Louis Stool, M.D.
Kenneth Trevino
Nancy Turret, M.S.W.
Richard Weiner, MD

VII. CURRICULUM VITAE

Curriculum vitae are provided in Appendix C for the following investigators:

Anouk Allart
Julie Adams, MD, MPH
Mohamed Aly, M.D.
Elisabeth Bernhardt
Marius Commodore, M.D.
Paul Croarkin, M.D.
C. Munro Cullum, Ph.D.Hayley Evans
Harry Goforth, M.D.
Austin Harrison
Mustafa M. Husain, M.D.
Andrew Kozel, M.D.
Sarah H. Lisanby, M.D.
Bruce Luber, Ph.D.
Mehul Mankad,, MD
Shawn M. McClintock, Ph.D.
Regena Mitschke
Scott Moore, M.D., Ph.D.
Najeeb Ranginwala, M.D.
Ahmad Raza, M.D.
Charlena Rodez
Moacyr Rosa, M.D.
Stefan Rowny, M.D.
Judy Shaw
David Steffens, M.D.
Louis Stool, M.D.
Kenneth Trevino
Nancy Turret, M.S.W.
Richard Weiner, MD

VIII. ADMINISTRATIVE INFORMATION

VIII.A SPONSOR

Department of Psychiatry & Behavioral Sciences
Box 3950
Duke University Medical Center
Durham, NC 27710
Phone: 919-684-5616
Fax: 919-681-5489.

VIII.B APPLICANT, CONTACT, AND CORRESPONDENCE ADDRESS

Sarah H. Lisanby, MD
Chair, Department of Psychiatry & Behavioral Sciences
Box 3950
Duke University Medical Center
Durham, NC 27710
Phone: 919-684-5616
Fax: 919-681-5489

VIII.C IRB Information

IRB STATUS – The IRBs are currently reviewing this protocol

DUHS IRB Office<br>Hock Plaza, Suite 405	Institutional Review Board<br>UT Southwestern Medical Center<br>5323 Harry Hines, Mail Code 8843
2424 Erwin Road, Campus Box 2712<br>Durham, NC 27705	Dallas, Texas 75390
RB Chairperson:<br>John Falletta, MD<br>Phone: (919) 668-5111<br>Fax: (919) 668-5125	IRB Chairperson:<br>David R. Karp, M.D.<br>Phone: 214-648-3060

VIII.D DEVICE LOCATIONS

One MST device will be used at each of two sites listed below.

Department of Psychiatry &Behavioral Sciences,<br>Duke University Medical Center,<br>Durham, NC 27710	Department of Psychiatry,<br>University of Texas Southwestern Medical Center<br>5323 Harry Hines, Mail Code 8898<br>Dallas, Texas 75390

VIII.E OTHER INSTITUTIONS

The devices will be used exclusively at the above listed institutions.

VIII.F Sales Information and Labeling

The Magstim Theta Repetitive Magnetic Stimulator in use at the New York State Psychiatric Institute was purchased by the Research Foundation for Mental Hygiene, Inc. at the New York State Psychiatric Institute from the Magstim Company Limited. The device in use at UTSW was purchased by the UTSW Department of Psychiatry from the Magstim Company Limited. The devices were purchased for research purposes and will not be sold. The devices and their manuals are labeled, "CAUTION—Investigational Device. Limited by Federal (or United States) law to investigational use." Research subjects will not be charged for the costs of the device. There are no subject payments to receive the treatment.

VIII.G Device Indications

The Magstim Theta Repetitive Magnetic Stimulator is indicated for investigational use only with local IRB approval of research protocols. All subjects must meet selection criteria for the IRB approved protocol, and must give written informed consent to participate in the research study. Use of the device is restricted to the active investigators listed in this application. The device will not be promoted or in any way represented as safe and effective for the use for which it is being investigated.

IX. RISK ANALYSIS

IX.A Possible Benefits

Convulsive therapy is an approved and effective treatment for major depression, but the therapeutic effects of using MST to perform convulsive therapy are not yet known.

IX.B Possible Risks

The potential risks of the study concern the use of MST, ECT, TMS (for MT determination and for MST), and those related to the evaluation procedures.

MST and ECT: It is possible that having had a seizure will have social ramifications for a patient. In order to mitigate these potential ramifications, study investigators will provide patients with a letter upon completion of treatment documenting that the seizure(s) were experimentally produced.

We acknowledge that, as evidenced by the literature [40-48], it is possible for subconvulsive TMS to induce mania in patients with bipolar disorder. However, our protocol does not use subconvulsive TMS. Instead, we are investigating an entirely different procedure entitled Magnetic Seizure Therapy (MST) which induces seizures. Because MST induces a seizure, it is more appropriate to extrapolate the risks of MST from studies of conventional electroconvulsive therapy (ECT). The literature does indicate that ECT can induce mania in bipolar patients [49-51]. However, the literature also indicates that if patients experiencing mania continue receive ECT treatment, the ECT acts as a mood stabilizer and effectively treats mania [52, 53]. In fact, patients with bipolar disorder tend to respond to ECT faster than unipolar patients, requiring fewer treatments to reach remission than those with unipolar depression[54]. Patients with bipolar disorder need more effective treatment options for their refractory depressions, therefore investigating MST as a treatment for this population addresses an important public health need. A magnetic form of convulsive therapy may indeed be uniquely suited for bipolar patients precisely because they are at increased risk of a mood switch into mania with medications and subconvulsive rTMS. ECT, in contrast to other treatments, has the unique capability of being able to effectively treat both the depressed and manic phases of bipolar disorder, and the risk of an acute mood switch is mitigated by the rapid anti-manic properties of ECT. Therefore, including patients with bipolar disorder who are not rapid cycling is indeed justified in this study of a novel form of convulsive therapy.

MST: We expect that the side effects of MST should be equal to or less than the side effects of ECT (discussed in detail below). Both treatments entail the induction of electricity in the brain and the initiation of a seizure. Magnetic fields do not penetrate as deeply as electric fields, suggesting that the cognitive side effects resulting from the stimulation of deeper brain structures should be less with MST than ECT. In the first case in which MST was conducted in humans, the only reported side effect was headache. In the first trial of 10 patients conducted at NYSPI, we found that MST had fewer acute cognitive side effects than ECT. In our second trial of MST (10 at NYSPI and 10 at UTSW), we found MST was well tolerated with substantially fewer cognitive side effects than patients receiving ECT in other studies simultaneously conducted. Subsequently a series of 10 patients have been treated with MST in Bern, Switzerland, with comparable results. More recently, a series of 6 patients have been treated with the Magstim Theta at maximal stimulator output, again with similar results. In none of these cases has there been a significant or unexpected adverse event. The cases of inadvertent seizure induction with rTMS in unanesthetized subjects have indicated no evidence of any long-term adverse medical or neurological consequences of rTMS-elicited seizures. Since MST is investigational, MST may not be effective, thus receiving MST may delay effective treatment. Delay in treatment of depression may result in

worsening of the clinical condition (e.g., intensified symptoms, suicidal ideation). As with all antidepressant treatments, including ECT, there is a theoretical risk of inducing mania.

There is a risk of decreased auditory threshold in people who receive MST for four or more weeks. In order to monitor any changes that occur, pure tone audiometry will be performed at baseline, after the sixth MST treatment, and after the last MST treatment. If a decrease in auditory threshold is detected at any point, patients will be re-tested periodically until either the threshold returns to baseline or until investigators determine that no further improvement will occur.

There has also been one documentation in the literature of dental pain occurring during and after a treatment course of TMS. Because of the similarities between MST and TMS, it is possible that patients undergoing MST could experience dental pain that might last past the end of the treatment course.

ECT: Immediately following ECT, patients are typically disoriented or confused. Following ECT sessions, some patients report headache, muscle soreness, or nausea. These side effects usually respond to simple treatment. Serious medical complications are rare. Dislocations, bone fractures, and dental complications have been reported with ECT, but have not been observed at NYSPI for the last 20 years. Cardiac complications are more common and constitute the leading cause of morbidity and mortality with ECT. The likelihood of these complications is reduced by (1) careful medical workup and the use of cardiology consultants in patients with significant preexisting cardiac disease; (2) careful monitoring of cardiac status during ECT; (3) modification of anesthetic procedures for prophylactic purposes (e.g., use of pharmacological agents to block hemodynamic changes). The availability of senior neuroanesthesiologists, who average several years of experience in conducting anesthesia for ECT, limits the development or sequelae of cardiac complications. Fatality associated with ECT is estimated to occur in 1/10,000 patients, and to our knowledge has never occurred at NYSPI.

ECT commonly results in memory deficits. These memory deficits are of two types: anterograde and retrograde amnesia. The anterograde amnesia involves a deficit in the capacity to retain newly formed information over delays (i.e. rapid forgetting). It is most intense immediately following a treatment, and displays rapid recovery. There is little objective evidence that anterograde amnestic deficits persist more than two weeks following an ECT course. In group data, studies at NYSPI have failed to observe persistent anterograde deficits. Retrograde amnesia pertains to a retrieval (and in some cases recognition) deficit for memory of events that occurred before and during the ECT course. This deficit also appears to be most marked immediately following a treatment. It usually pertains to recent memories, with more remote memories spared. While subject to rapid recovery, this deficit may never totally recede. Objective testing and subjective patient reports suggest that some patients will have permanent spottiness in memory for events that occurred close in time to the ECT course.

The magnitude of both memory deficits is sensitive to the parameters used in the conduct of ECT. In general, these deficits are more severe with bilateral versus right unilateral electrode placement, sine wave versus brief pulse stimulation, higher versus lower electrical intensity, closer spacing of the treatments, and larger number of treatments. Precautions that are taken in this protocol include the use of brief pulse, constant current stimulation and the titration of electrical dosage relative to seizure threshold. Further, the number of treatments is limited to that necessary to achieve clinical response or to ensure an adequate ECT trial. During the treatment course, there is careful monitoring of acute (post-itcal) and short-term (inter-ictal) cognitive side effects. Spacing of treatments may be increased or the treatment course terminated in the context of unacceptable side effects.

Evaluation Procedures: The medical evaluations present no risks beyond what is expected for routine clinical care of a patient undergoing a course of ECT. The procedures involve collection of blood through venipuncture for the initial screening blood work-up (25 ml).

Neuropsychological Testing: There may be a risk of fatigue from participation in the neuropsychological testing, but there are no other known risks from these procedures.

Medication Washout: Withdrawing antidepressant medication carries a risk of worsening the underlying depression. For this reason, we have shortened the period the patient must be off of antidepressant medications to 3 days (our previous ECT protocols required 5 days). Washout also carries a risk of withdrawal side effects, depending upon the specific medications.

Motor Cortex Excitability Studies/EEG: The methods used to evaluate motor threshold and other measures of motor cortex excitability involve the administration of single or paired pulse TMS given at long inter-pulse intervals. The most serious known risk of low frequency TMS is seizure. Low-frequency TMS has not been associated with seizure in appropriately screened individuals. If a patient has a seizure, he/she may require admission to a medical service and follow-up neurological evaluation. Having had a seizure may adversely affect medical insurability, future employment, and ability to drive. It is not known whether having had one seizure will make a person more prone to have future seizures. If a seizure occurs, the patient will be given a letter documenting that it was experimentally induced. The most commonly reported side effect of low frequency TMS is a "muscle-tension" type headache. About 17 out of every 100 people will experience a mild headache with this type of TMS. If a headache occurs, it usually starts during or immediately after the TMS and lasts from minutes to hours later. The headache usually goes away with standard over the counter pain medications (aspirin or acetaminophen). Neck pain may also occur, and it is also usually managed easily with standard over the counter painkillers. There may also be scalp discomfort due to contraction of scalp muscles. The clicking noise produced during the stimulation may temporarily affect hearing. The earplugs will reduce this risk.

Blood tests: Occasionally, redness or a bruise may develop at the site where blood was drawn. These side effects usually disappear in a few days.

IX.C Device and Site Safety

The following procedures will be in place to enhance safety at the site for the use of the device.

IX.C.1 Controlled Access Area

At each site, the safety measures recommended by the manufacturer regarding not operating the device in the context of cardiac pacemakers, metallic implants, and loose paramagnetic objects (Magstim operators manual) will be followed. These exclusions prevent the risk of pacemaker malfunction and the torque that may be exerted on metallic implants by the magnetic field. The device is placed more than 1 meter away from walls that are contiguous with passageways and thoroughfares. This protects the safety of people close to the perimeter of the room. Metallic objects are removed from the vicinity of the device (≤ 10 cm). This includes removing jewelry, scissors, needles, and other paramagnetic objects. Magnetic sensitive materials (e.g., watches, credit cards, computer disks) are also removed to avoid the possibility of damage. When not in use, the Magstim Theta Repetitive Magnetic Stimulator is disabled and the room housing it is locked.

IX.C.2 Screening of Subjects Prior to Stimulation

As described above, the screening assessment of potential subjects will identify and exclude from participation individuals with current or past medical conditions that may place them at increased risk for adverse effects from magnetic stimulation. Screening will include assessment of medical history, neurological history (seizure, stroke, brain lesion), head trauma, pregnancy, metallic implants, and implanted devices. Screening will be performed by physician evaluation, physical examination, and blood work.

IX.C.3 Protection From Excessive Noise

Earplugs will be worn by staff and patients prior to any magnetic stimulation to prevent exposure to excessive noise.

IX.C.4 Protection From Excessive Temperatures

The stimulation coil is insulated to retard heating. The coil contains 2 temperature sensors, providing continuous readouts. The controller software disables the device if coil temperature exceeds safe levels. Plastic or slotted EEG electrodes that have been shown on testing to avoid electrode heating with MST will be used in this study.

IX.C.5 Supervision of Subjects During Stimulation Sessions

MST sessions will be conducted by a physician with the participation of trained personnel. These personnel will be in visual and auditory contact with the patients at all times. Health status will be monitored continuously as described in the protocol.

IX.C.6 Emergency Power Interruption

In the event of an emergency, stimulation will be discontinued immediately by removing the coil from the head and then disabling the device.

IX.C.7 Controller Unit Safety Features

The dedicated computer controller software for the Magstim Theta Stimulator has internal limits on stimulation parameters. These limits prevent the device from being programmed to deliver pulses that exceed predetermined limits. The software logs all pulses administered, as well as parameter values.

IX.C.8 Emergency Medical Procedures

If at any moment during the stimulation session there is a clinical suspicion of a possible serious adverse health effect, the coil will be immediately removed from the patient’s head and the stimulator will be disabled. Each ECT suite is staffed by medical personnel with extensive experience in ECT and has access to emergency equipment (oxygen, iv medications) to handle potential adverse events.

IX.C.9 Fire Precautions

Fire extinguishers are available and easily accessible in both ECT suites. Fire precautions are in accordance with local hospital regulations.

IX.C.10 Environmental Impact Claim for Exclusion

We claim categorical exclusion for this study as provided for in 21 CFR section 25.24 (e) 7. The devices shipped under the Investigational Device Exemption are intended to be used for clinical studies in which waste will be controlled or the amount of waste expected to enter the environment may reasonably be expected to be nontoxic.

IX.D RISK/BENEFIT ANALYSIS

As outlined above, extensive precautions will be taken to ensure of the safety of study participants and investigators. All patients participating in this study will receive treatment with a full course of convulsive therapy. This study has the potential of contributing to the development of a treatment method (MST) for major depressive disorder that may have a superior risk/benefit ratio than conventional convulsive therapy. Consequently, the potential benefits of this study substantially outweigh the risks.

X. DURATION OF THE STUDY

The expected duration of the study is approximately three years.

XI. REFERENCES

1. Kessler RC, McGonagle KA, Zhao S, et al. Lifetime and 12-month prevalence of DSM-III-R psychiatric disorders in the United States. Results from the National Comorbidity Survey. Arch Gen Psychiatry. Jan 1994;51(1):8-19.
2. Evans DL, Charney DS. Mood disorders and medical illness: a major public health problem. Biol Psychiatry. Aug 1 2003;54(3):177-180.
3. Michaud CM, Murray CJ, Bloom BR. Burden of disease--implications for future research. Jama. Feb 7 2001;285(5):535-539.
4. Panel DG. Depression in Primary Care, Vol.2 Treatment of Major Depression. Clinical Practice Guideline, Number 5. Vol AHCPR Publication No. 93-0551. Rockville, MD: US Department of Health and Human Services; 1999.
5. Trivedi MH, Rush AJ, Wisniewski SR, et al. Evaluation of outcomes with citalopram for depression using measurement-based care in STAR*D: implications for clinical practice.[see comment]. American Journal of Psychiatry. 2006;163(1):28-40.
6. Rush AJ, Trivedi MH, Wisniewski SR, et al. Acute and longer-term outcomes in depressed outpatients requiring one or several treatment steps: a STAR*D report. American Journal of Psychiatry. 2006;163:1905-1917.
7. American Psychiatric Association. Task Force on Electroconvulsive Therapy. The Practive of electroconvulsive Therapy: Recommendations for Treatment, Training, and Privileging. Washington DC: American Psychiatric Association; 2001.
8. Lisanby SH, Maddox JH, Prudic J, Devanand DP, Sackeim HA. The effects of electroconvulsive therapy on memory of autobiographical and public events.[see comment]. Archives of General Psychiatry. 2000;57(6):581-590.
9. Sackeim H, Prudic J, Fuller R, Keilp J, Lavori P, Olfson M. The cognitive effects of electroconvulsive therapy in community settings. Neuropsychopharmacology. 2007;32:244-254.
10. Mulsant BH, Rosen J, Thornton JE, Zubenko GS. A prospective naturalistic study of electroconvulsive therapy in late-life depression. Journal of Geriatric Psychiatry & Neurology. 1991;4(1):3-13.
11. Lisanby SH, Luber B, Schlaepfer TE, Sackeim HA. Safety and feasibility of magnetic seizure therapy (MST) in major depression: randomized within-subject comparison with electroconvulsive therapy. Neuropsychopharmacology. 2003;28(10):1852-1865.
12. Lisanby S, Husain M, Morales O, et al. Controlled clinical trial of the antidepressant efficacy of magnetic seizure therapy in the treatment of major depression. ACNP Annual Meeting Abstracts. 2003:166.
13. Lisanby SH, Schlaepfer TE, Fisch HU, Sackeim HA. Magnetic seizure therapy of major depression. Archives of General Psychiatry. 2001;58(3):303-305.
14. Lisanby SH. Update on magnetic seizure therapy: a novel form of convulsive therapy. Journal of ECT. 2002;18(4):182-188.
15. Kosel M, Frick C, Lisanby SH, Fisch HU, Schlaepfer TE. Magnetic seizure therapy improves mood in refractory major depression. Neuropsychopharmacology. 2003;28(11):2045-2048.
16. Lisanby SH, Morales O, Payne N, et al. New developments in electroconvulsive therapy and magnetic seizure therapy. Cns Spectrums. 2003;8(7):529-536.
17. Lisanby SH, Moscrip T, Morales O, Luber B, Schroeder C, Sackeim HA. Neurophysiological characterization of magnetic seizure therapy (MST) in non-human primates. Supplements to Clinical Neurophysiology. 2003;56:81-99.

18. Dwork AJ, Arango V, Underwood M, et al. Absence of histological lesions in primate models of ECT and magnetic seizure therapy.[see comment]. American Journal of Psychiatry. 2004;161(3):576-578.
19. Moscrip TD, Terrace HS, Sackeim HA, Lisanby SH. Randomized controlled trial of the cognitive side-effects of magnetic seizure therapy (MST) and electroconvulsive shock (ECS). International Journal of Neuropsychopharmacology. 2006;9(1):1-11.
20. White PF, Amos Q, Zhang Y, et al. Anesthetic considerations for magnetic seizure therapy: a novel therapy for severe depression. Anesthesia & Analgesia. 2006;103(1):76-80.
21. Lisanby SH. Magnetic Seizure Therapy: Development of a Novel Convulsive Technique. In: LIsanby SH, ed. Brain Stimulation in Psychiatric Treatment. Vol 23. Washington, DC: American Psychiatric Publishing, Inc; 2004.
22. Lisanby SH, Luber B, Finck AD, Schroeder C, Sackeim HA. Deliberate seizure induction with repetitive transcranial magnetic stimulation in nonhuman primates.[erratum appears in Arch Gen Psychiatry 2001 May;58(5):515]. Archives of General Psychiatry. 2001;58(2):199-200.
23. Geddes LA, Baker LE. The specific resistance of biological material--a compendium of data for the biomedical engineer and physiologist. Medical & Biological Engineering. 1967;5(3):271-293.
24. Sackeim HA, Long J, Luber B, et al. Physical properties and quantification of the ECT stimulus: I. Basic principles.[see comment]. Convulsive Therapy. 1994;10(2):93-123.
25. Sackeim HA, Luber B, Katzman GP, et al. The effects of electroconvulsive therapy on quantitative electroencephalograms. Relationship to clinical outcome.[see comment]. Archives of General Psychiatry. 1996;53(9):814-824.
26. Sackeim HA, Luber B, Moeller JR, Prudic J, Devanand DP, Nobler MS. Electrophysiological correlates of the adverse cognitive effects of electroconvulsive therapy. Journal of ECT. 2000;16(2):110-120.
27. Nobler MS, Luber B, Moeller JR, et al. Quantitative EEG during seizures induced by electroconvulsive therapy: relations to treatment modality and clinical features. I. Global analyses.[see comment]. Journal of ECT. 2000;16(3):211-228.
28. Luber B, Nobler MS, Moeller JR, et al. Quantitative EEG during seizures induced by electroconvulsive therapy: relations to treatment modality and clinical features. II. Topographic analyses. Journal of ECT. 2000;16(3):229-243.
29. Nobler MS, Oquendo MA, Kegeles LS, et al. Decreased regional brain metabolism after ect. American Journal of Psychiatry. 2001;158(2):305-308.
30. Barker AT, Jalinous R, Freeston IL. Non-invasive magnetic stimulation of human motor cortex. Lancet. 1985;1(8437):1106-1107.
31. Davey K, Epstein CM, George MS, Bohning DE. Modeling the effects of electrical conductivity of the head on the induced electric field in the brain during magnetic stimulation. Clinical Neurophysiology. 2003;114(11):2204-2209.
32. Bohning DE, Pecheny AP, Epstein CM, et al. Mapping transcranial magnetic stimulation (TMS) fields in vivo with MRI. Neuroreport. 1997;8(11):2535-2538.
33. Bohning DE, He L, George MS, Epstein CM. Deconvolution of transcranial magnetic stimulation (TMS) maps. Journal of Neural Transmission. 2001;108(1):35-52.
34. Epstein CM, Schwartzberg DG, Davey KR, Sudderth DB. Localizing the site of magnetic brain stimulation in humans.[see comment]. Neurology. 1990;40(4):666-670.
35. Sackeim HA. The anticonvulsant hypothesis of the mechanisms of action of ECT: current status. Journal of ECT. 1999;15(1):5-26.

36. Prohovnik I, Sackeim HA, Decina P, Malitz S. Acute reductions of regional cerebral blood flow following electroconvulsive therapy. Interactions with modality and time. Annals of the New York Academy of Sciences. 1986;462:249-262.
37. Scalia J, Lisanby S, Underwood M, et al. The spatial distribution of mossy fiber sprouting in a non-human primate model for electroconvulsive therapy and magnetic seizure therapy. Biological Psychiatry. 2004;55:207S.
38. Scalia J, Lisanby S, Underwood M, et al. Structural changes in the primate hippocampus following convulsive intervention: Potential links with cognitive effects. J ECT. 2004;20:65.
39. Peterchev AV, Kirov G, Ebmeier K, Scott A, Husain M, Lisanby SH. Frontiers in TMS technology development: Controllable pulse shape TMS (cTMS) and Magnetic Seizure Therapy (MST) at 100 Hz. Biol Psychiatry. 2007;In Press:Abstract.
40. Dolberg OT, Schreiber S, Grunhaus L. Transcranial magnetic stimulation-induced switch into mania: a report of two cases. Biol Psychiatry. Mar 1 2001;49(5):468-470.
41. Ella R, Zwanzger P, Stampfer R, et al. Switch to mania after slow rTMS of the right prefrontal cortex. J Clin Psychiatry. Mar 2002;63(3):249.
42. Garcia-Toro M. Acute manic symptomatology during repetitive transcranial magnetic stimulation in a patient with bipolar depression. Br J Psychiatry. Nov 1999;175:491.
43. Hausmann A, Kramer-Reinstadler K, Lechner-Schoner T, et al. Can bilateral prefrontal repetitive transcranial magnetic stimulation (rTMS) induce mania? A case report. J Clin Psychiatry. Nov 2004;65(11):1575-1576.
44. Hausmann A, Pascual-Leone A, Kemmler G, et al. No deterioration of cognitive performance in an aggressive unilateral and bilateral antidepressant rTMS add-on trial. J Clin Psychiatry. Jun 2004;65(6):772-782.
45. Huang CC, Su TP, Shan IK. A case report of repetitive transcranial magnetic stimulation-induced mania. Bipolar Disord. Oct 2004;6(5):444-445.
46. Nahas Z, Kozel FA, Li X, Anderson B, George MS. Left prefrontal transcranial magnetic stimulation (TMS) treatment of depression in bipolar affective disorder: a pilot study of acute safety and efficacy. Bipolar Disord. Feb 2003;5(1):40-47.
47. Sakkas P, Mihalopoulou P, Mourtzouhou P, et al. Induction of mania by rTMS: report of two cases. Eur Psychiatry. Jun 2003;18(4):196-198.
48. Su TP, Huang CC, Wei IH. Add-on rTMS for medication-resistant depression: a randomized, double-blind, sham-controlled trial in Chinese patients. J Clin Psychiatry. Jul 2005;66(7):930-937.
49. Andrade C GB, Swaminath G, et al. Mania as a side effect of electroconvulsive therapy. Convulsive Therapy. 1988;4:81-83.
50. Andrade C GB, Channabasavanna SM. Further characterization of mania as a side effect of ECT. Convulsive Therapy. 1990;6:318-319.
51. Devanand DP, Sackeim HA, Decina P, Prudic J. The development of mania and organic euphoria during ECT. J Clin Psychiatry. Feb 1988;49(2):69-71.
52. Volpe FM, Tavares A. Manic patients receiving ECT in a Brazilian sample. J Affect Disord. Apr 2004;79(1-3):201-208.
53. Kusumakar V, Yatham LN, Haslam DR, et al. Treatment of mania, mixed state, and rapid cycling. Can J Psychiatry. Aug 1997;42 Suppl 2:79S-86S.
54. Daly JJ, Prudic J, Devanand DP, et al. ECT in bipolar and unipolar depression: differences in speed of response. Bipolar Disord. Apr 2001;3(2):95-104.

Statistical Analysis Plan

Background and hypothesis

The purpose of this between-subject study will be to determine whether seizure induction with magnetic seizure therapy (MST) is effective in treating severe mood disorder, and to compare MST with conventional electroconvulsive therapy (ECT) in terms of efficacy and side effect profile. We hypothesized that MST and ECT will have similar antidepressant efficacy.

Study design

This is a 3-center randomized controlled trial. Phase 1 is a double-masked, randomized controlled trial that contrasts the efficacy and safety of MST (high-dose, circular coil vertex placement) and ECT (six times seizure threshold, ultrabrief pulse width, right unilateral electrode placement). Eligible patients are randomized 1:1. The blind will be maintained until the last patient has completed the study. Phase 2, the follow up, provided a descriptive characterization of longer-term outcomes, i.e., long-term durability of benefit and/or persistence of side effects.

Outcome Measures

The primary outcome measure is the 24-item Hamilton Rating Scale for Depression ($HDRS_{24}$) in the intent-to-treat sample. Response and remission status will provide secondary outcome measures to evaluate clinical significance. Additional secondary outcome measures include the Inventory of Depressive Symptomatology – Clinician-Rated ($IDS\text{-}C_{30}$) and Self Report ($IDS\text{-}SR_{30}$), Global Assessment of Functioning (GAF), and Clinical and Patient Global Impression – Improvement (CGI-I, PGI-I). This study also aims to contrast the cognitive side effects of MST and ECT. We hypothesize that ECT and MST will differ in the extent of post-treatment amnesia as reflected in primary measures of anterograde and retrograde amnesia following the acute treatment phase.

Ratings are conducted at baseline, within 24 to 48 hours of each MST/ECT session, 24–72 hours after the last MST/ECT session, and at 2 and 6 month follow up. Consistent with the standard practice in ECT, there is no predetermined minimum or maximum number of treatments. The aim is to continue treatment until the patient achieves maximal improvement. If there is no significant clinical improvement by the 8th session ($\geq$ 25% drop in $HDRS_{24}$ from baseline at any point within the first 8 treatments), treatment will be stopped. Patients will be dropped from the study will be given routine clinical care with a treatment of known efficacy as clinically indicated by the study team. Otherwise, treatment will be continued until there has been a plateau or the subject achieves remission, whichever comes first. A plateau is defined as a decrease of 3 points or fewer in $HDRS_{24}$ score between a given treatment and the two successive treatments; this determination is only made starting with treatment 8. Clinical response is defined as $\geq$ 50% drop in $HDRS_{24}$. Remission is defined as $\geq$ 60% drop in $HDRS_{24}$ and $\leq$ 8.

Statistical analysis

An intent-to-treat (ITT) analysis was performed. Effects of the treatment session on $HDRS_{24}$ and time to orientation were analyzed using repeated measures analysis. A repeated effect of treatment session was used to model the covariance of the residuals with a compound symmetry structure. Patients are specified as a random factor. In addition to the main effects of treatment group (ECT vs MST) and treatment session, we also assessed the group-by-session interaction. Other continuous demographic and outcome variables were analyzed with t-tests; categorical variables were analyzed with χ^2 analyses. For all analyses, statistical significance is defined as a two-sided p-value of less than .05. Finally, Kaplan–Meier analysis is used to compare time to remission between the ECT and MST groups.
