# Supplementary material for: Clinical Outcomes of Magnetic Seizure Therapy vs Electroconvulsive Therapy for Major Depressive Episode: A Randomized Clinical Trial
Source: JAMA Psychiatry. 2023 Dec 6;81(3):240–9. doi: 10.1001/jamapsychiatry.2023.4599 (PMC10701670; doi:10.1001/jamapsychiatry.2023.4599)
Supplement: Supplement 2. — eFigure 1. Inventory of Depressive Symptomatology (IDS) eFigure 2. Seizure Duration and Time to Orientation [file jamapsychiatry-e234599-s002.pdf]

Supplemental Online Content

Deng ZD, Luber B, McClintock SM, Weiner RD, Husain MM, Lisanby SH. Clinical outcomes of magnetic seizure therapy vs electroconvulsive therapy for major depressive episode: a randomized clinical trial. *JAMA Psychiatry*. Published online December 6, 2023. doi:10.1001/jamapsychiatry.2023.4599

eFigure 1. Inventory of Depressive Symptomatology (IDS)
eFigure 2. Seizure Duration and Time to Orientation

This supplemental material has been provided by the authors to give readers additional information about their work.

© 2023 Deng ZD. *JAMA Psychiatry.*

eFigure 1. Inventory of Depressive Symptomatology (IDS)

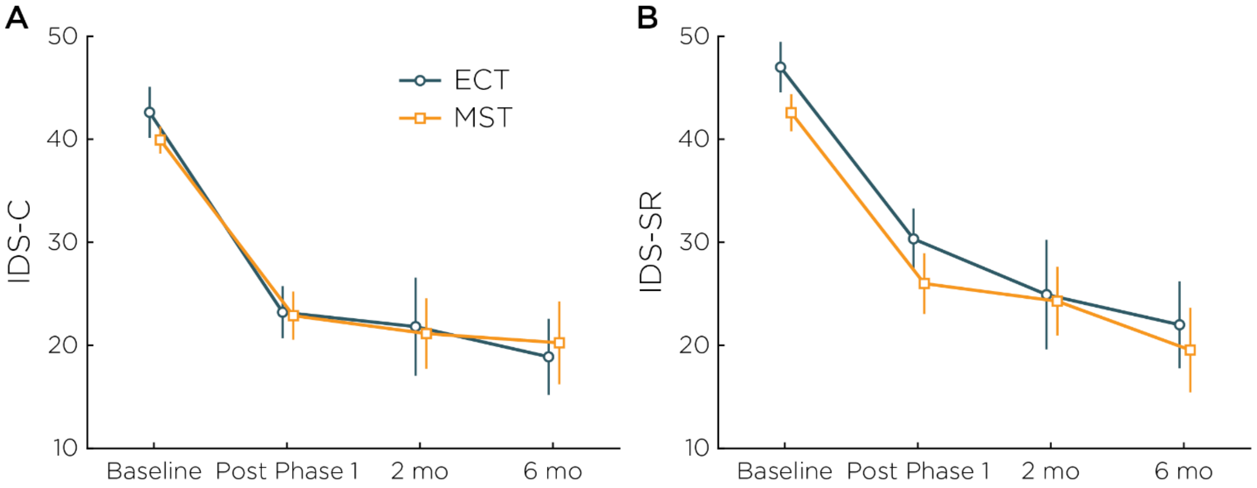

A, Clinician-rated (IDS-C). B, self-report (IDS-SR). The 2 × 2 mixed-model ANOVA (between-group factor: MST vs ECT; repeated-measures factor: baseline vs end of acute treatment course) using the IDS-C scores showed a significant main effect of time (F = 98.0, P < .001) but no significant effect of group (F = 0.1, P = .77) or group-by-session interaction (F = 0.1, P = .81). IDS-SR scores showed a significant main effect of time (F = 54.8, P < .001) but no effect of group (F = 1.6, P = .22) or group-by-session interaction (F = 0.0, P > .99).

© 2023 Deng ZD. *JAMA Psychiatry.*

eFigure 2. Seizure Duration and Time to Orientation

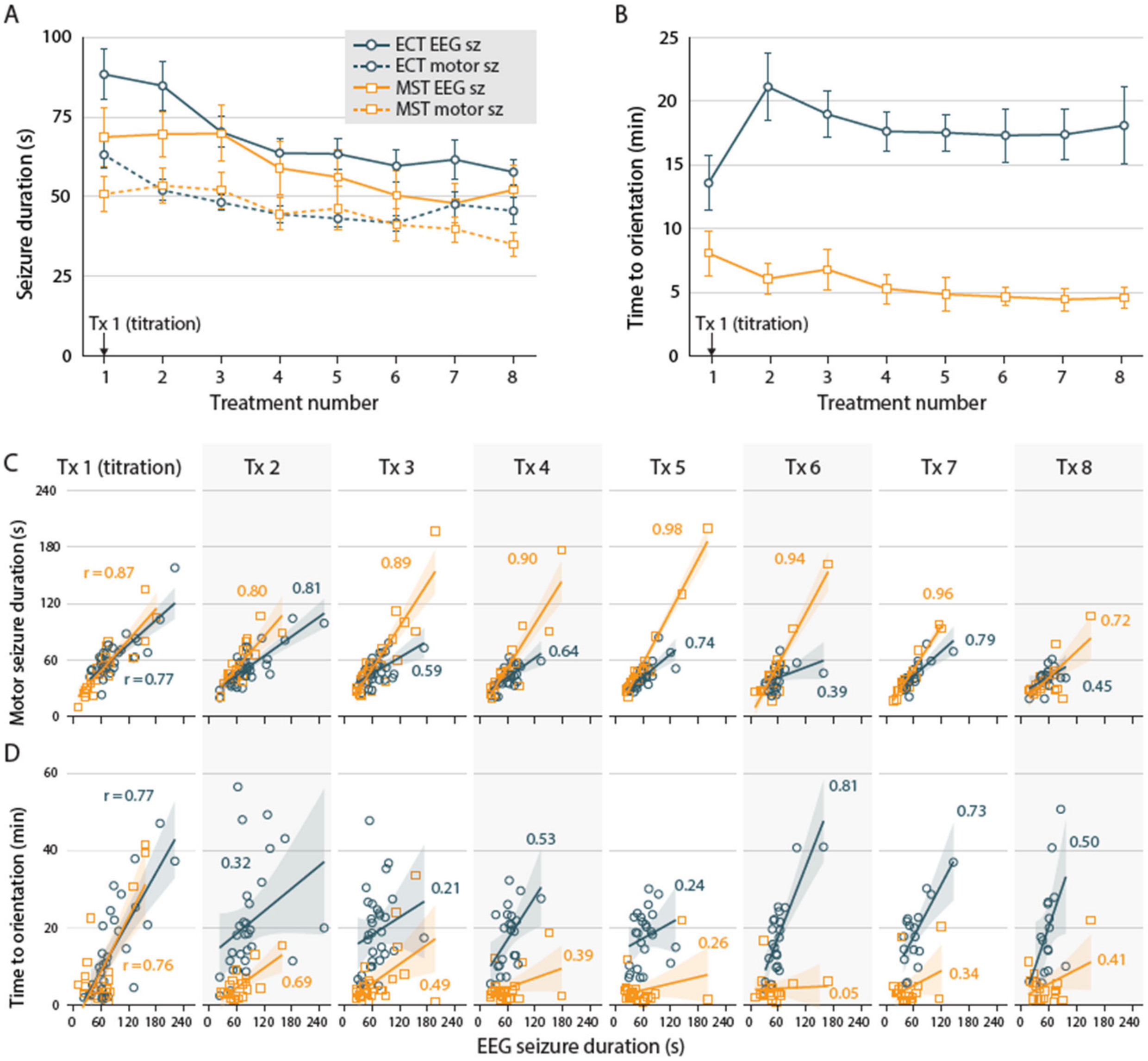

Data are presented for the first 8 treatments. A, Electroencephalographic (EEG) and motor seizure duration. The mixed-effects model showed that the mean motor seizure duration decreased as a function of treatment number (up to treatment 8; $t = -6.38$, $P < .001$) and did not differ between electroconvulsive therapy (ECT) and magnetic seizure therapy (MST) ($t = -0.46$, $P = .65$). The mean frontal EEG seizure duration also decreased as a function of treatment number ($t = -6.72$, $P < .001$) but did not differ between ECT and MST ($t = -1.31$, $P = .20$). B, Time to orientation. Participants regained orientation faster following MST than ECT at both threshold ($F = 10.0$, $P = .003$) and suprathreshold ($F = 62.9$, $P < .001$) levels. C, Session-by-session correlation between EEG seizure duration and motor seizure duration. The ratio of EEG to motor seizure duration for ECT was larger than that for MST ($t = -3.23$, $P = .002$) and did not change with treatment number ($t = 0.027$, $P = .98$), suggesting that the seizure spread beyond the motor cortex in the case of ECT but was more focal for MST. D, Session-by-session correlation between EEG seizure duration and time to orientation. Seizure duration was associated with time to orientation for both ECT and MST.

© 2023 Deng ZD. *JAMA Psychiatry.*
