# Supplementary material for: Clinical Outcomes of Magnetic Seizure Therapy vs Electroconvulsive Therapy for Major Depressive Episode: A Randomized Clinical Trial
Source: JAMA Psychiatry. 2023 Dec 6;81(3):240–9. doi: 10.1001/jamapsychiatry.2023.4599 (PMC10701670; doi:10.1001/jamapsychiatry.2023.4599)
Supplement: Supplement 3. — Data Sharing Statement [file jamapsychiatry-e234599-s003.pdf]

Data Sharing Statement

Deng. Clinical Outcomes of Magnetic Seizure Therapy vs Electroconvulsive Therapy for Major Depressive Episode. *JAMA Psychiatry*. Published December 06, 2023.
doi:10.1001/jamapsychiatry.2023.4599

Data
Data available: Yes
Data types: Deidentified participant data, Data dictionary
How to access data: The data that support the findings of this study are available on request.
When available: With publication

Supporting Documents
Document types: Statistical/analytic code
How to access documents: The data that support the findings of this study are available on request.
When available: With publication

Additional Information
Who can access the data: Researchers whose proposed use of the data has been approved.
Types of analyses: For any purpose
Mechanisms of data availability: with investigator support
